# Red Light Absorption of [Re^I^(CO)_3_(α-diimine)Cl] Complexes through Extension of the 4,4′-Bipyrimidine Ligand’s π-System

**DOI:** 10.3390/molecules28041905

**Published:** 2023-02-16

**Authors:** Nicolas Meitinger, Subrata Mandal, Dieter Sorsche, Andrea Pannwitz, Sven Rau

**Affiliations:** Institute for Inorganic Chemistry I, Ulm University, Albert-Einstein-Allee 11, 89081 Ulm, Germany

**Keywords:** Rhenium(I) complexes, NN chelate ligands, α-diimine, π-extension, azaperylene, spectroscopy, electrochemistry, UV–Vis NIR spectroelectrochemistry, photochemistry, X-ray crystal structures

## Abstract

Rhenium(I) complexes of type [Re(CO)_3_(NN)Cl] (NN = α-diimine) with MLCT absorption in the orange-red region of the visible spectrum have been synthesized and fully characterized, including single crystal X-ray diffraction on two complexes. The strong bathochromic shift of MLCT absorption was achieved through extension of the π-system of the electron-poor bidiazine ligand 4,4′-bipyrimidine by the addition of fused phenyl rings, resulting in 4,4′-biquinazoline. Furthermore, upon anionic cyclization of the twisted bidiazine, a new 4N-doped perylene ligand, namely, 1,3,10,12-tetraazaperylene, was obtained. Electrochemical characterization revealed a significant stabilization of the LUMO in this series, with the first reduction of the azaperylene found at $E_{1/2}^{\left(0/-\right)}$ = −1.131 V vs. Fc^+^/Fc, which is the most anodic half-wave potential observed for N-doped perylene derivatives so far. The low LUMO energies were directly correlated to the photophysical properties of the respective complexes, resulting in a strongly red-shifted MLCT absorption band in chloroform with a λ_max_ = 586 nm and high extinction coefficients (ε_586nm_ > 5000 M^−1^ cm^−1^) ranging above 700 nm in the case of the tetraazaperylene complex. Such low-energy MLCT absorption is highly unusual for Re(I) α-diimine complexes, for which these bands are typically found in the near UV. The reported 1,3,10,12-tetraazaperylene complex displayed the [Re(CO)_3_(α-diimine)Cl] complex with the strongest MLCT red shift ever reported. UV–Vis NIR spectroelectrochemical investigations gave further insights into the nature and stability of the reduced states. The electron-poor ligands explored herein open up a new path for designing metal complexes with strongly red-shifted absorption, thus enabling photocatalysis and photomedical applications with low-energy, tissue-penetrating red light in future.

## 1. Introduction

In the context of light energy conversion, e.g., in light-driven catalysis and photodynamic therapy, it is desirable to develop chromophores that absorb strongly in the low energy region of the visible spectrum of light, as this provides energy efficiency during the use of artificial light sources, as well as enables applications under physiological conditions and with deeper tissue penetration [1,2,3,4,5,6]. A promising class of compounds in this regard are complexes of the type [Re(CO)_3_(NN)Cl] (NN = α-diimine) [7,8,9,10], as they can be functionalized for various applications [11,12,13], including light-driven catalysis [14,15,16,17] and biomedical applications [18,19,20]. Previous reports have highlighted that their photophysical properties can be bathochromically shifted by introducing electron-poor, aromatic diimine ligands. In particular, metal-to-ligand charge transfer (MLCT) energies could be shifted towards lower energies via either increased π-conjugation in the α-diimine acceptor ligand [21,22,23], the introduction of electron-withdrawing substituents on the α-diimine ligands [24,25,26,27], or the use of electron-poor ligands, e.g., bidiazines [28,29]. The reported maxima of MLCT transitions in such Re(I) complexes range by up to 529 nm [30,31]. However, biomedical applications in living tissue, such as photodynamic therapy [32] or light-triggered ligand release [33], require significant absorbances within the so-called therapeutic window of 600 to 800 nm [34].

**Scheme 1:**
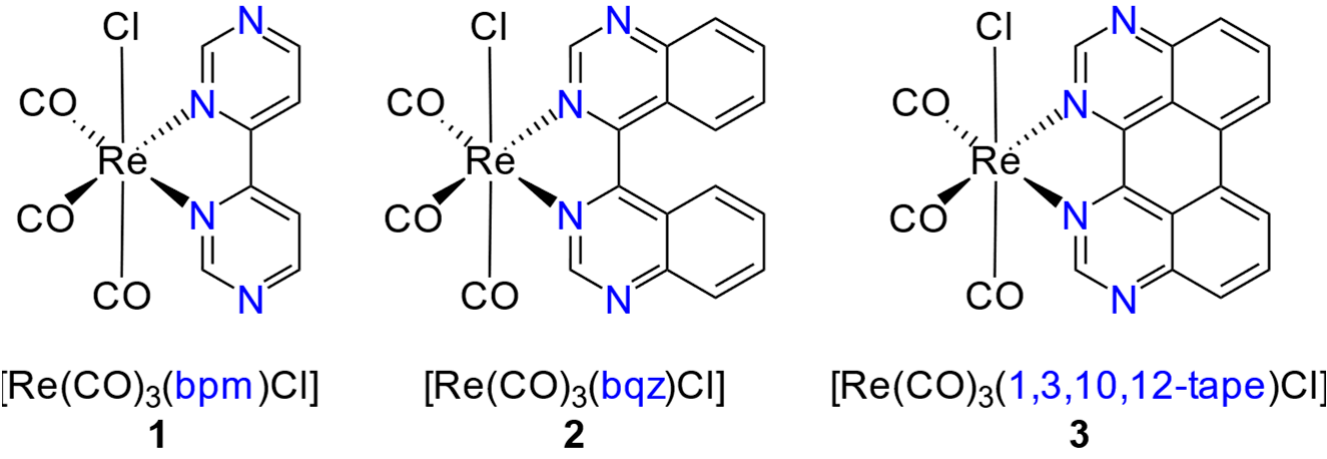
[Re(CO)_3_(NN)Cl] complexes studied herein with the bidentate ligands NN (**bpm** = 4,4′-bipyrimidine, **bqz** = 4,4′-biquinazoline, and **1,3,10,12-tape** = 1,3,10,12-tetraazaperylene).

When comparing several [Re(CO)_3_(NN)Cl] complexes with different isomers of the bidentate bidiazine ligands, the 4,4′-bipyrimidine (**bpm**) ligand has the lowest-lying π* energy levels [28], making it a very promising ligand for red-shifted MLCT energies (see compound **1** in Scheme 1). The properties of [Re(CO)_3_(**bpm**)Cl] (**1**) have been reported [29,35,36], and we took it as a starting point to further extend the π-system of the **bpm** ligand. The introduction of some typical aromatic substituents into the 6,6′-position of the **bpm** (dpb = 6,6′-diphenyl-, dmpb = 6,6′-*p-*methoxyphenyl-, dnb = 6,6′-(1-naphythyl)-4,4′-bipyrimdine) was reported to yield only minor effects on the photophysical properties [35]; thus, we decided to introduce *fused* rings to the **bpm** ligand to yield 4,4′-biquinazoline (**bqz**, compound **2** in Scheme 1), as this ligand was reported to significantly red-shift the MLCT absorption of respective Ru(II) complexes [37,38].

In a second step, we increased the fused aromatic ring system even further by cyclizing the **bqz** ligand to yield the previously unknown organic molecule 1,3,10,12-tetraazaperylene (**1,3,10,12-tape**) and its respective Re(I) complex (compound **3** in Scheme 1). We herein report on the synthesis, the electrochemical and spectroscopic properties of long-wavelength absorbing, and the π-extended complexes of type [Re(CO)_3_(NN)Cl] of these ligands in different solvent environments. UV–Vis NIR spectroelectrochemical measurements were performed to gain further insight into the nature of their reduced states.

## 2. Results

### 2.1. Syntheses and Structural Characterization

**Scheme 2:**
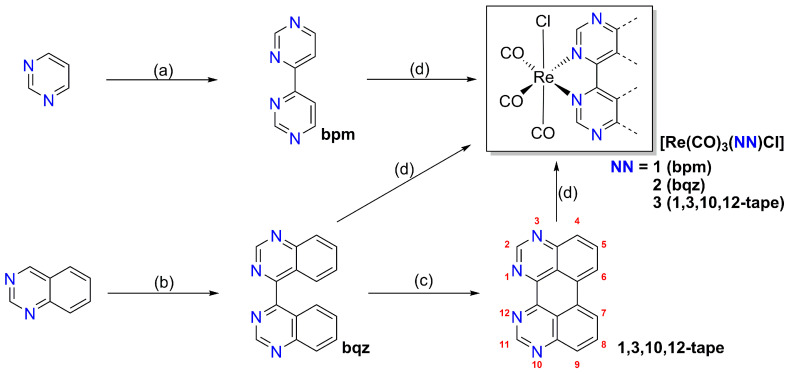
Overview of synthetic procedures to obtain [Re(CO)_3_(NN)Cl] complexes based on the 4,4′-bipyrimidine coordination sphere. (**a**) (1) Na (3 eq.), THF, RT, Ar, 18 h; (2) EtOH, TEA, O_2_, 2 h, 63%; (**b**) (1) TMPH, *n*-BuLi, THF, −10 °C, Ar, 0.5 h; (2) quinazoline, −78 °C, Ar, 3.5 h; (3) RT, O_2_, 15 h, 56%; (**c**) (1) K (12 eq.), DME, RT, Ar, 750 rpm, 70 h; (2) THF, O_2_, RT, 2 h, 73%; (**d**) [Re(CO)_5_Cl], toluene, 110 °C, Ar, 18 h; **1**: 61%, **2**: 66%, **3** 77%. Nomenclature for positions of perylene given in red.

[Re(CO)_3_(NN)Cl] complexes **1**–**3** were obtained in good yields by the thermal replacement of CO from the carbonylmetal chloride precursor [Re(CO)_5_Cl] with equimolar amounts of the respective bidentate ligand NN in toluene under inert conditions, as depicted in Scheme 2.

The **bpm** ligand of complex **1** was synthesized via a reported radical anion coupling of pyrimidine that was mediated by sodium in THF [39]. The **bqz** ligand of complex **2** was synthesized following a modified reported procedure [40] upon lithiation of quinazoline with in situ generated LiTMP (TMPH = 2,2,6,6-tetramethylpiperidine) and subsequent oxidation by molecular oxygen. The previously unknown 4N-doped azaperylene ligand **1,3,10,12-tape** was prepared from **bqz** via an anionic cyclization reaction at room temperature that was induced by excess potassium in 1,2-dimethoxyethane (DME) solution under an argon atmosphere (Ar), in a similar manner to the syntheses of other azaperylene derivatives [41,42,43], and the cyclized ligand was obtained upon subsequent re-oxidation by gently bubbling oxygen through the solution.

The structural characterization of all ligands and complexes, including the new electron-deficient ligand **1,3,10,12-tape**, was carried out using ^1^H- and ^13^C- as well as *C,H*-heterodinuclear NMR experiments. Based on the symmetry observed in NMR experiments, complexes **1**–**3** selectively formed in *fac* configuration. No concentration dependence was observed in chemical shifts at concentrations ranging from 1 mM to 33 mM, which indicated that there were no intermolecular interactions under these conditions (Figures S18, S21 and S23). HR-ESI-MS data were in accordance with the expected ^m^/_z_ values. In case of the complexes, acetonitrile adducts formed upon halide ligand exchange during ionization, resulting in singly positively charged ions. Solid-state ATR-FTIR spectra of the complexes showed the expected signals of carbonyl stretching bands in the range of 2200–1800 cm^−1^. All data and detailed procedures are reported in the Experimental section, and spectra are shown in the Supplementary Materials.

### 2.2. Crystallography

Single crystals suitable for XRD analyses were obtained of compounds **1** and **3** through the slow vapor diffusion of *n*-hexane into a concentrated chloroform solution of the respective complex. Both solid-state structures confirmed the constitution of these complexes with the respective ligands binding to a chlorido tris (carbonyl) rhenium(I) fragment and a *fac* conformation of the CO ligands. Bond lengths between the metal ion and chlorido, as well as carbonyl ligands, were within the typical range (Tables S1 and S2). While we were unable to obtain single crystals of **2**, we point out that a single crystal XRD structure of the related chlorido tris (carbonyl) rhenium biquinoline complex has previously been communicated to the CSD by Holdt et al., with CSD deposition number 631826 [44]. The reported structure (Figure S33) shows a significant twist between the two quinoline moieties, which can be reasoned to be a consequence of steric repulsion between the two quinoline units, as was reasoned previously for [Ru(bpy)_2_(**bqz**)]^2+^ [38].

#### 2.2.1. Details about **1**

**Figure 1:**
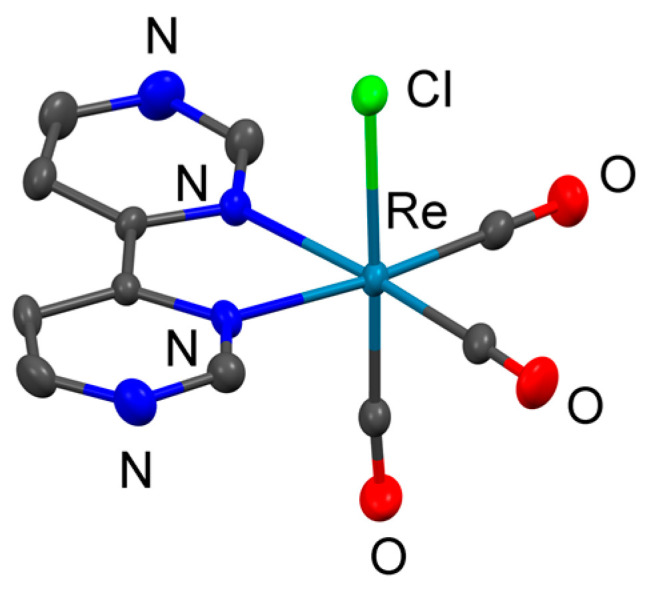
Molecular structure of **1** as determined by scXRD analysis; ellipsoid depiction created with Mercury 2020.2.0 (Build 290188); ellipsoids at 50% probability; hydrogen atoms omitted for clarity.

Compound **1** was crystallized in the triclinic space group P$\bar{1}$, and the asymmetric unit contains two symmetrically inequivalent complexes (Figure S34). Figure 1 shows the representative molecular structure of one of the two complexes found in the unit cell, as there were no notable differences in bond angles and distances between the two units. Interestingly, the **bpm** ligand appeared slightly distorted at the 2 and 2′ positions, namely between the two heteroatoms. The angle ∢(NCN) was slightly larger than the expected 120°, i.e., at 125.75° on average (standard deviation 0.33°). Concomitantly, the angles around the nitrogen atoms were slightly smaller than 120°, i.e., 116.68° on average (standard deviation 0.4875°). A summary of all angles can be found in the Supplementary Materials (Table S1). This geometry gives the N-C-N corner a flattened appearance. This distortion has also been found in related bipyrimidine ligands and their metal complexes [35,38,45].

#### 2.2.2. Details about **3**

**Figure 2:**
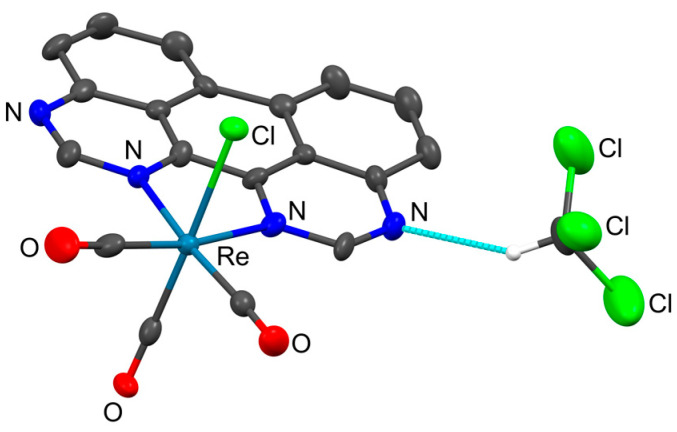
Molecular structure of **3**·CHCl_3_ as determined by scXRD analysis showing the asymmetric unit including one molecule of chloroform; blue dashed line indicating a small contact between the 1,3,10,12-tape ligand and chloroform; created with Mercury 2020.2.0 (Build 290188); ellipsoids at 50% probability; hydrogens except the one of chloroform omitted for clarity.

Compound **3** was crystallized in the monoclinic space group P2_1_/c. The asymmetric unit is depicted in Figure 2 and shows one molecule of the complex, for which one molecule of chloroform with a short contact interaction to one of the terminal nitrogen atoms of the **1,3,10,12-tape** ligand was observed. This C-H···N distance was 3.41 Å between the non-H-atoms, which is within only 0.2 Å below the sum of van der Waals radii and, therefore, indicates a weak short contact interaction. Nonetheless, this observation is indicative of a general susceptibility of the **1,3,10,12-tape** ligand to hydrogen bond donating solvents. 

**Figure 3:**
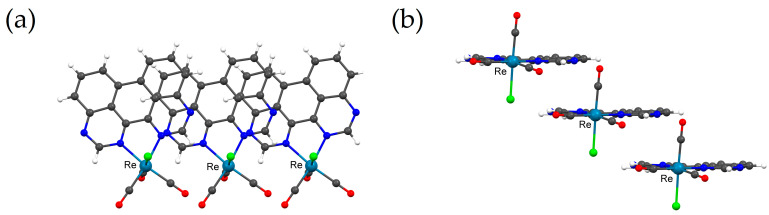
Packing of **3**·CHCl_3_ in the solid state as determined by scXRD analysis; ball-and-stick depictions showcasing minor intermolecular stacking with a coplanar overlap of 1,3,10,12-tetraazaperylene ligands’ π-surfaces in a staircase-like fashion. (**a**) top view; (**b**) side view; solvent molecules (CHCl_3_) omitted for clarity; created with Mercury 2020.2.0 (Build 290188).

The packing within the solid-state structure reveals partial intermolecular stacking with a coplanar overlap of the aromatic ligands’ π-surfaces and an intermolecular distance of 3.404 Å between the ligand planes (Figure 3). The **1,3,10,12-tape** ligand was slightly distorted from planarity to evade steric repulsion between the two protons on C6 and C7. This twist was characterized by an average deviation of atoms from the mean plane 0.0669 Å, and maximum deviations of 0.1597 Å and 0.1294 Å at C5 and C7 (compare Figure S35). The ligand plane in this complex was tilted by 11.56° compared to the coordination plane defined by rhenium and the two carbonyl ligands in *trans* position to the **1,3,10,12-tape** ligand (see Figure S36), which is not unusual and may be attributed to packing effects [35].

### 2.3. Electrochemistry

**Figure 4:**
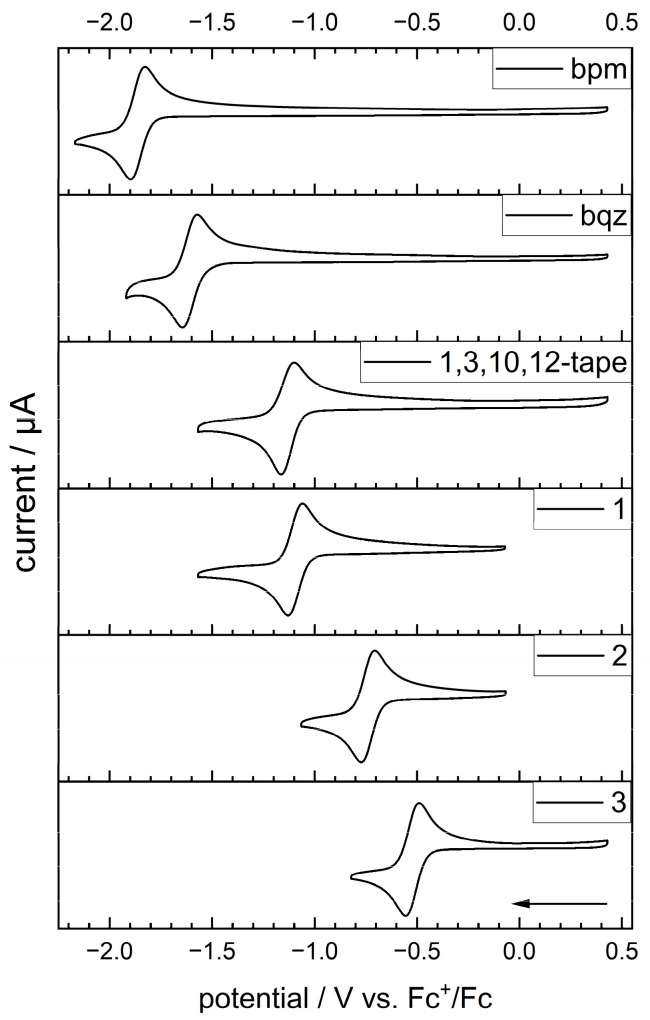
Cyclic voltammograms of ligands and complexes showing the first reduction. DMF, 0.1 M *n*Bu_4_NPF_6_ supporting electrolyte, 1 mM analyte, 100 mV s^−1^, GC-WE, Pt-wire CE, non-aqueous AgNO_3_/Ag-RE (0.1 M nBu_4_NPF_6_/0.01 mM AgNO_3_ in MeCN), referenced against Fc^+^/Fc.

The cyclic voltammetry in DMF with 0.1 M *n*Bu_4_NPF_6_ supporting electrolyte at 100 mV s^−1^ scan rate gave reversible, one-electron reduction waves for the complexes as well as their bidentate ligands, which corresponded to the formation of radical anionic species localized on the diimine ligand-based π* orbitals, as has been shown by Kaim via EPR spectroscopy for complex **1** [46], as well as for other metal complexes of the **bpm** ligand [37,47]. The results are summarized in Table 1, and cyclovoltammograms for the reversible first reductions are shown in Figure 4 (see also Figure S39 for CVs, including further irreversible reductions). The electrochemical data for **bpm** [48] and complex **1** [46] were in accordance with published results.

In a series of isomeric bidiazine ligands compared to 2,2′-bipyridine (**bpy)** and based on Hückel molecular orbital perturbation calculations, the **bpm** ligand was found to exhibit the strongest stabilization of LUMO energy due to the nitrogen centers in the 4,4′-position of a perturbed biphenyl π-system [28]. Extending the π-system of the **bpm** by introducing two additional phenyl rings in **bqz** anodically shifted the electrochemical potential of the first reduction by 254 mV ($E_{1/2}^{\left(0/-\right)}$) and the second, irreversible reduction by 511 mV ($E_{pc}^{\left(-/2-\right)}$) compared to **bpm**. Remarkably, cyclization of the **bqz** to the azaperylene **1,3,10,12-tape** yielded an even stronger anodic shift of 732 mV ($E_{1/2}^{\left(0/-\right)}$) and 783 mV ($E_{pc}^{\left(-/2-\right)}$) for the first and second reduction, respectively, which highlights the lowering of LUMO energies with an increase in π-conjugation. Due to the positioning of nitrogen centers in the 4,4′-position, a similar stabilization effect of LUMO energy as for the **bpm** was observed for **1,3,10,12-tape**, which was about 150 mV more easily reduced than the reported **1,6,7,12-tape** bridging ligand (ca. −1.28 V vs. Fc^+^/Fc measured in CH_2_Cl_2_) [42] and, thus, was reported as the azaperylene with the lowest LUMO energy published so far.

Compared to the free ligands, coordination to the [Re(CO)_3_Cl]^+^ fragment led to additional strong anodic shifts by 768 mV (**1**), 871 mV (**2**) and 608 mV (**3**) for the half-wave potentials of the reversible first reductions, respectively. These shifts are due to the polarizing effect of the metal fragment, which are generally observed for coordination to [Re(CO)_3_Cl]^+^ as well as [Ru(bpy)_2_]^2+^ fragments [29,37,38]. Directly increasing the π-system of the **bpm**, rather than introducing aryl substituents into the 6,6′-positions of the ligand [35], led to significantly lower-lying LUMO levels, thereby making complexes **2** and **3** much more readily reduced.

The second electron uptake was found to be irreversible in all cases (Figure S39), irrespective of the applied scan rate, which resulted in a low peak current during the anodic scan at the re-oxidation potential of ca. −1.1 V vs. Fc^+^/Fc and a shoulder towards less negative potential for **3** (Figure S40). This indicates that a twofold reduction results in chemical changes of the complexes. However, the fact that two consecutive reduction bands were observed indicates that reduction in dry DMF does not lead to full protonation of the ligands, as proton-coupled electron transfer would yield a single two-electron, two-proton reduction wave, as was observed for **bpm** in aqueous media at neutral pH by Zoric et al. [49]. The differences in redox potential between the first and second reduction of the respective ligand compared to their complexes were similar among each other, with a maximum difference for **2** of +111 mV, presumably due to forcing the biheterocycle into a more coplanar arrangement with steric repulsion of the protons, whereas for **3**, the potential difference exactly matched the one for free **1,3,10,12-tape**.

Notably, for complex **3**, even a third irreversible reduction wave was found close to the end of the solvent potential window at $E_{pc}^{\left(2-/3-\right)}$ −2.072 V vs. Fc^+^/Fc.

We did not observe oxidation of the Re^I^ center within the potential window of DMF; hence, an oxidation of the complexes could be expected at $E_{pa}^{\left(+1/0\right)}$ > 1.13 V vs. Fc^+^/Fc. According to Klein et al., an irreversible oxidation is expected at higher potentials, resulting in labile 17 electron Re(II) species that quickly undergo displacement of carbonyl ligands by solvent molecules [46]. As was in line with our results, Ioachim et al. observed that, in their series of [Re(CO)_3_(6,6′-diaryl-4,4′-bpm)X] complexes, an increase in π-acceptor properties of the diimine ligand led to a stabilization of Re(I), thus anodically shifting the potentials for irreversible abstraction of an electron from the metal center. The oxidation potential can, however, be cathodically shifted by replacing the axial chlorido ligand with neutral σ-donors [35].

### 2.4. Electronic Absorption

**Figure 5:**
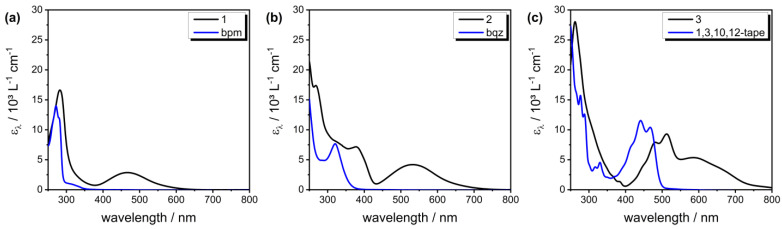
UV–Vis absorption spectra (molar absorption coefficient vs. wavelength) of complex (**a**) **1**, (**b**) **2**, and (**c**) **3** in CHCl_3_ along with their respective ligands (**NN**). Blue line and black line denote the ligand and complex, respectively.

The UV–Vis absorption spectra of complexes **1**–**3,** as well as their respective bidentate ligands in CHCl_3_, are shown in Figure 5, and the absorption maxima and corresponding extinction coefficients are summarized in Table 2. Whereas the ligands **bpm** and **bqz** showed typical π–π* and n–π* bands in the UV, the planarized oligoaromatic ligand **1,3,10,12-tape** showed major absorption in the visible region of the spectrum, with two maxima at 440 nm and 467 nm, and a shoulder at 414 nm (Figure 5). These features were attributed to a vibrational fine-structure corresponding to S_0_→S_n_ transitions, as it has been observed for perylene and other azaperylene derivatives [43] and has been assigned based on a TD-DFT approach [50]. Such vibrational fine-structures are also seen for oligoaromatic perylene diimides [51]. Absorption of the **1,3,10,12-tape** in chloroform and acetonitrile followed a Beer–Lambert behavior in a concentration range of ~20 nM–25 µM, and no shifts were observed, which indicated the absence of intermolecular supramolecular interactions, such as π–π stacking. (Figures S41 and S42).

Upon coordination of the ligands to the [Re(CO)_3_Cl]^+^ fragment, the ligand-based absorption features were strongly red-shifted in the cases of complexes **2** and **3,** which was probably due to the strong Lewis acid character of the Re center and its influence on the ligands’ orbital energies related to this electronic transition. A similar red-shift was previously observed in azaperylenes upon coordination of protons and can be attributed to the n–π* character of the electronic transition [43]. Absorption of the complexes followed a Beer–Lambert behavior in a concentration range of ~20–100 µM. (Figures S43 and S44).

In addition to the ligand-based absorption bands, the complexes displayed a prominent absorption band at a relatively longer wavelength, which was assigned to the metal-to-ligand charge transfer (MLCT) absorption [29,35]. Notably, within the series of complexes **1**–**3** and with increasing degrees of π-delocalization, the MLCT absorption bands bathochromically shifted, and extinction coefficients increased. The MLCT maximum of **1** was found at 467 nm (ε_467_ = 2910 M^−1^ cm^−1^), of **2** at 532 nm (ε_532_ = 4350 M^−1^ cm^−1^), and of **3** at 586 nm (ε_586_ = 5170 M^−1^ cm^−1^) in chloroform, respectively. Thus, these electronic transitions, in the cases of complexes **2** and **3,** also covered absorption in the yellow-red region of the visible spectrum, thus resulting in (dark) purple complexes and extending well into the therapeutic window for **3**. These findings are rather surprising, as MLCT maxima of the literature-known [Re(CO)_3_(NN)Cl] complexes are typically found in the near UV (for NN = bpy), up to about 400–500 nm for more electron-deficient NN ligands such as 3,3′-bipyridazine (**bpdz**) [29] or 6,6′-diaryl-4,4′-**bpm** [35] (see Table 2 for more examples). 

The trend of a red-shifted electronic transition with increased π-system and increased planarity is typical for complexes having extended aromatic systems [22,38,46,53] and is attributed to a combination of lowering the energy of the acceptors’ π* orbitals, as well as steric restraints from the ligands’ rigidity lowering the symmetry of the coordination geometry to thereby weaken the ligand field [38]. The influence of steric effects of the flexible vs. rigid ligand backbone can be seen in the reported single crystal structures, where complex **3** exhibits two tilted ligand planes of the Re(NN) plane with respect to the opposite Re(CO)_2_ plane that are co-planar in complex **1** and tilted by 11.56° in complex **3**. In summary, a bathochromic shift in absorption and an increase in molar extinction coefficients for the MLCT maxima were observed upon extending the π-system in the series of **1** < **2** < **3**. The opposite trend in extinction coefficients is observed when the **bpm** ligand’s π-system is increased by 6,6′-diarylation (Table 2) [35].

#### Solvatochromism

**Figure 6:**
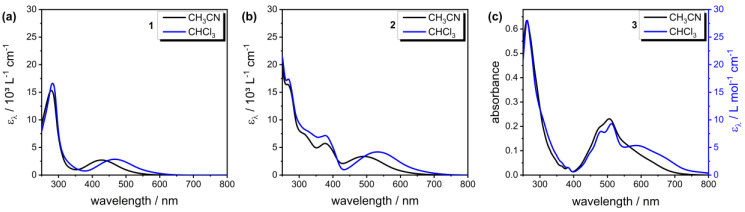
UV–Vis absorption spectra (molar absorption coefficient vs. wavelength) of complex (**a**) **1**, (**b**) **2** and (**c**) **3** in CHCl_3_ (blue line) and CH_3_CN (black line). Complex **3** is poorly soluble in CH_3_CN, thus the absorbance is plotted against wavelength for comparison.

The UV–Vis absorption spectra of complexes **1**–**3** were also recorded in the more polar solvent CH_3_CN, as are shown in Figure 6 and Table 2**.** Unfortunately, complex **3** was poorly soluble in CH_3_CN, and only qualitative absorption spectra were recorded. While the effect on the ligand-based transition was minor (0–6 nm), comparing the MLCT bands for all the complexes in CHCl_3_ and CH_3_CN showed sensitivity towards solvent polarity and hypsochromic shifts of around 40 nm when changing to the more polar solvent. This behavior, referred to as ‘negative solvatochromism’, is also found for MLCT-based transitions of various Mo(0) W(0), Mn(I) and Re(I) based carbonyl complexes [52,54,55,56,57,58] and is explained by a reduced excited-state electric dipole moment in such complexes and a more polar ground state, which is stabilized in more polar solvents, thus increasing the HOMO-LUMO gap to result in a hypsochromic shift [58]. Additionally, the weak hydrogen bonding interactions of the CHCl_3_ solvent observed in the single crystal structure of **3** might have resulted in the additional stabilization of the ligand-based π* orbital.

### 2.5. UV–Vis NIR Spectroelectrochemistry

**Figure 7:**
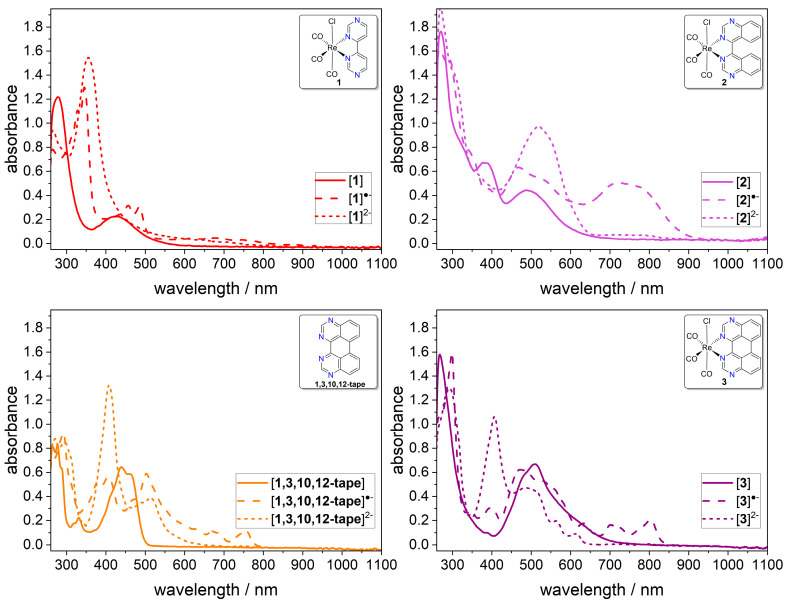
In situ UV–Vis NIR absorption spectra of the neutral (solid line), one-electron reduced (dashed line) and two-electron reduced species (dotted line) generated in a spectroelectrochemical cell (Pt-WE, Pt-wire CE, AgCl/Ag pseudo-RE, potentials referenced against Fc^+^/Fc). Deaerated DMF containing 0.1 M *n*Bu_4_NPF_6_ and 0.5 mM analyte, scan rate 100 mV s^−1^.

Reduced forms of the complexes were generated in a UV–Vis NIR spectroelectrochemical cell (see Experimental part for details) in order to obtain further information on the nature and stability of these species. Electronic absorption spectra were measured in deaerated DMF with 0.1 M *n*Bu_4_NPF_6_ supporting electrolyte upon equilibrating the 0.5 mM analyte solutions at adequate potentials for 90–180 s (Figures S50–S57). The absorption maxima that were obtained are summarized in Table 3. A spectroscopic study of the reduced states of the **bpm** ligand in DMF, along with theoretical calculations, has been published by Krejčík et al. [59]. Neutral **bpm** showed intense π–π* and weak n–π* transitions at 272 and 318 nm, while, in its reduced state (**bpm**^●−^), it showed an intense π–π* electronic transition into the now singly occupied molecular orbital (SOMO) at 362 nm, as well as several π–π* transitions in the Vis-NIR region. These longer-wavelength absorptions according to the theoretical calculations originate from electronic transitions from the anion radical’s SOMO into energetically higher-lying π* orbitals (Table 3) [59]. Compared to the neutral ligands (L), reduced ligands [**bpm**]^●−^, [**bqz**]^●−^ and [**1,3,10,12-tape**]^●−^ are expected to display weaker π-acceptors, but stronger σ-donors. The π–acceptor interactions between d_π_(Re) and the π*(L) orbitals are expected to decrease, leading to a hypsochromic shift of the related MLCT transition with lower intensity, as the π*-SOMO is partially populated. Furthermore, new intraligand (IL) transitions are anticipated due to the electron now available in the π* orbital. Bathochromically shifted IL transitions have also been observed in structurally related Ru(II) complexes of 1,12-diazaperylene and the bridging ligand **1,6,7,12-tape** [42]. Figure 7 shows the spectral changes upon reduction of the ligand **1,3,10,12-tape** and complexes **1**–**3**.

The reduction of complex **1** to [**1**]^●−^ led to very similar spectral changes as those discussed for the ligand **bpm**, with an intense absorption at 345 nm that was tentatively assigned to π–π* and n–π* transitions into the SOMO, two maxima at 456 and 486 nm due to π–π* transitions from the SOMO, and several broad, weak transitions in the longer wavelength region from 530–950 nm, which were potentially due to symmetry-forbidden π–π* transitions [59]. A stable isosbestic point was observed at 307 nm, and the spectra obtained prior to electrolysis and after reversed electrolysis matched; thus, we judge the one-electron reduction to be reversible (Figure S50). In contrast, the second reduction proved to be irreversible, as going from [**1**]^●−^ to [**1**]^2−^ did not result in isosbestic points (Figure S51). The loss of Vis absorption for [**1**]^2−^ confirmed the assignment of these transitions to be related to the SOMO that was now fully occupied. For [**1**]^2−^, an intense band at 355 nm was observed, again similar to the absorbance of **bpm**^2−^ with an absorption maximum at 363 nm [59]. We therefore concluded that a metal-to-ligand charge transfer was not possible in [**1**]^2−^.

For complex **2**, reduction to [**2**]^●−^ led to a strong increase in absorption, with maxima at 468 and 718 nm, and stable isosbestic points could be observed at 284, 362, and 420 nm, which proved the first reduction to be reversible (Figure S52). Surprisingly, even for [**2**]^2−^, an intense Vis absorption of up to 650 nm was observed, and although no isosbestic points were seen going from [**2**]^●−^ to [**2**]^2−^, the original spectrum of **2** was regained after reversed electrolysis (Figure S53). More detailed studies on the ligand **bqz**, as well as theoretical calculations, will be necessary to assign related transitions in this tilted ligand.

Changes in the electronic absorbances of the ligand **1,3,10,12-tape** and its complex **3** upon reduction are shown in Figure 7. As was already discussed when comparing the spectra of reduced **bpm** and complex **1**, the shape of the absorption spectra obtained upon reduction of **1,3,10,12-tape** and **3** was very similar in their respective reduced states (for a direct comparison see Figure S58). The π–π* transitions found around 278 nm and the n–π* transitions around 331 nm for **1,3,10,12-tape** bathochromically shifted to 292 and 380 nm upon reduction to [**1,3,10,12-tape**]^●−^, which was accompanied by an increase in intensity with isosbestic points at 399 and 476 nm (Figure S54). Presumably, these transitions were again related to electronic excitations into the SOMO, whereas transitions triggered by visible light in the range of 450–800 nm were related to π–π* excitations originating from the SOMO, based on the assignments made for **bpm** [59]. Upon further reduction to [**1,3,10,12-tape**]^2−^, the broad absorption around 407 nm increased in intensity, whereas around 513 nm, a decrease was observed, and transitions above 650 nm diminished. Isosbestic points were found at 326, 377, and 456 nm, and the initial spectrum of the **1,3,10,12-tape** was regained upon reversed electrolysis, such that, from a spectroscopic point of view, the second reduction was also found to be reversible (Figure S55).

The reduction of **3** to [**3**]^●−^ gave stable isosbestic points at 284, 318, 331, 486, 533, 593, and 624 nm, and the initial spectrum was regained upon reversed electrolysis (Figure S56). The π–π* transitions of **3** at 268 nm shifted to 298 nm, and new bands appeared in the 350–420 nm range due to excitation into the SOMO. The broad band of **3** from 420–665 nm, with some MLCT character, broadened on both sides of the initial maximum at 509 nm. Several new transitions appeared from 665–850 nm that might be related to π–π* transitions originating from the SOMO again, as they were also observed for **1,3,10,12-tape**, although they shifted to higher energy. As for the ligand, from a spectroscopic point of view, the second reduction was also reversible in the case of **3**, with isosbestic points during the conversion of [**3**]^●−^ to [**3**]^2−^ existing at 306, 337, 343, 349, and 445 nm, as well as a regained initial spectrum after reversed electrolysis (Figure S57). For [**3**]^2−^, a much more intense absorption centered at 407 nm was observed, while, in the range of 445–640 nm, the intensity decreased and was lost above 640 nm.

### 2.6. Steady-State Fluorescence

In contrast to published results [29], where a very weak emission of **1** centered at 662 nm was found, none of the complexes **1**, **2** or **3** showed any emission upon excitation at 436 nm (Figure S49) with our detector system in neither chloroform (aerated/inert) nor THF solution (inert).

Within the series of the ligands, and upon excitation into the π–π* transitions, only **1,3,10,12-tape** was found to be emissive, both in chloroform and acetonitrile solution (Figures S45–S48). Compared to perylene, the emission of the azaperylene was considerably broadened, which is a trend that has been shown for a series of other azaperylenes recently [43]. Whereas the sharp, highly structured signals for perylene were found at 438, 466, and 496 nm respectively, the structureless emission of the **1,3,10,12-tape** ranging from 450–650 nm showed a maximum intensity at 512 nm in acetonitrile solution, whereas in chloroform, the emission maximum was found at 501 nm with a shoulder at 527 nm. The shape of the **1,3,10,12-tape** emission showed even less vibrational fine-structure than that of the reported bridging ligand **1,6,7,12-tape** [43]. Emission studies were carried out at concentrations ranging from 20 nM to 25 µM, where the shape and position of the luminescence spectra remained constant. Furthermore, the emission intensity linearly correlated to the sample concentration in both solvents, which suggested no significant intermolecular interactions or excimer formation in solution which would have been anticipated for large aromatic systems such as perylene [60].

### 2.7. Transient Absorption

No long-lived excited state species was observed on the ns–time scale using transient absorption spectroscopy (Figures S59–S61).

## 3. Conclusions and Outlook

In summary, we synthesized and characterized a new 4N-doped perylene ligand, 1,3,10,12-tetraazaperylene, along with two new molecular Re(I) complexes of type [Re(CO)_3_(NN)Cl], where NN represents an electron-poor bidentate ligand based on 4,4′-bipyrimidine with different extensions of π-conjugations. The single crystal structures of complexes **1** and **3** confirmed their constitution in *fac* configuration and revealed a strong influence of the ligand on the coordination geometry at the Re center, with a tilt of the octahedral environment in **3**. As confirmed by electrochemical analyzes, the LUMO energy of the smallest analogous ligand, 4,4′-bipyrimidine, was further stabilized in going from 4,4′-biquinazoline to the cyclized 1,3,10,12-tetrazaperylene upon increasing π delocalization. This resulted in a bathochromic shift of MLCT absorption in the series of Re complexes, thus confirming that increasing the π-system of the ligand results in a favorable broadened absorption of visible light, which, to the best of our knowledge, led to the most red-shifted MLCT absorption of structurally related [Re(CO)_3_(NN)Cl] complexes observed so far. For **3**, this absorption extended well into the therapeutic window for applications in PDT and biology. The maxima of the charge–transfer bands are sensitive to variations in solvent polarity (negative solvatochromism). In addition, relatively high MLCT extinction coefficients were observed compared to other, structurally related [Re(CO)_3_(NN)Cl] complexes, which were increasing in the row **1** < **2** < **3**. UV–Vis NIR spectroelectrochemical investigations gave further insight into the nature and stability of the reduced states, where a twofold reduction of the 1,3,10,12-tape ligand and its complex **3** were found to be reversible from a spectroscopic point of view. Theoretical calculations might give insights into the unusual observed trend in extinction coefficients and the transitions in the single and double reduced states.

In this study, in the absence of oxygen, no long-lived excited states were observed. However, upon substitution of the axial chlorido ligand with neutral σ-donor ligands, longer-lived excited states might become accessible. It is worth noting that other non-luminescent [Re(CO)_3_(NN)X] complexes with weak red-light absorption have already been successfully utilized in electrocatalytic CO_2_ reduction, as well as in photodynamic therapy [61,62]. Theoretical calculations might give insights into excited state deactivation pathways and provide further guidelines for ligand design to yield long-lived excited states that are able to engage in photo-redox activity or photodynamic therapy. 

## 4. Materials and Methods

If not otherwise stated, all reactions were carried out using Schlenk techniques with dry solvents under an argon atmosphere. Commercially available reagents were purchased in highest available purity from Sigma-Aldrich Chemie GmbH (Taufkirchen, Germany), or Thermo Fisher Scientific GmbH (Schwerte, Germany) and used without further purification. Thin-layer chromatography was performed on aluminum plates, precoated with silica gel, of Merck Si_60_ F_254_. Preparative column chromatography was carried out on glass columns packed with silica gel, which was Supelco silica gel (technical grade, pore size 60 Å, 230–400 mesh particle size, 40–63 µm particle size).

**NMR** spectra were recorded on a Bruker (Karlsruhe, Germany) Avance Neo 400 or 600 at 293 K (400/600 MHz for ^1^H-NMR, 101/151 MHz for ^13^C-NMR) and processed with MestReNova software (Version 14.2.0). Chemical shift values δ are given in parts per million (ppm) and were referenced using residual non-deuterated solvent peaks as an internal standard. The splitting patterns are labeled as follows: s = singlet, d = doublet, t = triplet, q = quartet, m = multiplet and dd = doublet of doublets. Coupling constants J are presented as absolute values in Hz. HR-ESI-**MS** spectra were recorded using a Bruker (Karlsruhe, Germany) solariX. Data were processed with Bruker Daltonik Compass DataAnalysisViewer software (Version 5.0). Values are given in fractions ^m^/_z_. Solid-state ATR-**FTIR** spectra were measured using a Bruker (Karlsruhe, Germany) ALPHA II FT-IR spectrometer equipped with platinum ATR accessory mounting samples in between monolithic diamond crystals. Air was used as a background in a range of 4000–400 cm^−1^, with 24 averaged scans and a resolution of 4 cm^−1^. Crystals suitable for single crystal **X-ray** crystallography were mounted using a MicroLoop (MiTeGen, Ithaca, New York, USA) and perfluoropolyalkyl ether (viscosity 1800 cSt; Sigma Aldrich Chemie GmbH (Taufkirchen, Germany)). X-ray diffraction intensity data were measured at 150 K on a Bruker (Karlsruhe, Germany) D8 Quest single crystal diffractometer with a PHOTON II detector using Mo K_α_ radiation (wavelength λ = 0.71073 Å). Structure solution and refinement were carried out using the SHELXL package via Olex2 [63,64]. Corrections for incident and diffracted beam absorption effects were applied using multi-scan refinements. Structures were solved by direct methods and refined against F2 by using the full-matrix least-squares technique. The hydrogen atoms were included at calculated positions with fixed thermal parameters. All non-hydrogen atoms were refined anisotropically unless otherwise mentioned. Mercury 2020.2.0 (Build 290188) was used for structural representations [65]. **Cyclic voltammetry** experiments were carried using DMF (Acros Organics, 99.8%, extra dry over molecular sieve, Thermo Fisher Scientific GmbH, Schwerte, Germany) with 0.1 M *n*Bu_4_NPF_6_ as supporting electrolyte, in 0.1 mM analyte concentration. Measurements were performed using a CH Instruments (Austin, Texas, USA) Electrochemical Analyzer potentiostat CHI730E in three-electrode configuration. A glassy carbon disc with a 3 mm diameter stick was applied as a working electrode, a Pt wire as a counter electrode, and a non-aqueous AgNO_3_/Ag electrode (0.1 M *n*Bu_4_NPF_6_, 0.01 M AgNO_3_ in MeCN) as a reference electrode. All voltammograms were referenced with respect to the Fc^+^/Fc couple upon addition thereof after each measurement; thus, potentials are given versus Fc^+^/Fc. Various scan rates were applied, and, unless otherwise stated, 100 mV/s were used. CVs were automatically iR-compensated. **UV–Vis** absorption spectra were recorded in Starna GmbH (Pfungstadt, Germany) quartz cuvettes with 10 mm pathlength using JASCO Deutschland GmbH (Pfungstadt, Germany) Photometers V-670 and V-760 and were baseline corrected. **UV–Vis NIR spectroelectrochemical** measurements were performed on a Pine Research (Durham, North Carolina, USA) WaveDriver200 potentiostat connected to an Avantes (Apeldoorn, The Netherlands; Mountain Photonics GmbH, Landsberg am Lech, Germany) AvaLight-DHC full-range compact light source and an Avantes (Apeldoorn, The Netherlands; Mountain Photonics GmbH, Landsberg am Lech, Germany) AvaSpec-UL S2048 UV–Vis fiber-optic spectrometer. The spectroelectrochemical cell consisted of a special quartz glass cuvette with an optical path length of 1.7 mm, using a Pine Research (Durham, North Carolina, USA) honeycomb screen-printed platinum electrode as a working and counter electrode and a silver-wire pseudo-reference electrode (silver wire in glass frit containing electrolyte solution, DMF/0.1 M *n*Bu_4_NPF_6_). Potentials are given relative to the Fc^+^/Fc internal standard. Experiments were performed in extra dry DMF (Acros Organics, 99.8%, extra dry over molecular sieve, Thermo Fisher Scientific GmbH, Schwerte, Germany) containing 0.1 M *n*Bu_4_NPF_6_ as supporting electrolyte. Background spectra were recorded using pure electrolyte solution. Analyte solutions (0.5 mM) were deaerated by gently purging with argon and kept under an argon atmosphere while performing the experiments. A scan rate of 100 mV s^−1^ was utilized, and the potential was equilibrated for 90–180 s after each step (100–200 mV) before taking an absorption spectrum. Measurements started at a potential where only the neutral species were in solution, scanned cathodic first until the vertex potential was reached, then anodic for reoxidation of the negatively charged species. **Fluorescence** spectra were recorded on a JASCO Deutschland GmbH (Pfungstadt, Germany) FP-8500. Experiments were performed at room temperature under aerobic or argon-saturated conditions. Complex emission was probed using a Horiba Jobin-Yvon GmbH (Bensheim, Germany) FluoroMax Plus-C automated benchtop spectrofluorometer equipped with a 150 W Xe arc excitation lamp (horizontal, continuous wave), a R13456 photon-counting PMT detector (190–930 nm; multialkali photodiode) and Czerny–Turner monochromators with 1200 groove/mm gratings blazed at 330 nm (excitation) and 500 nm (emission). Emission spectra were corrected by multiplying with wavelength-specific factors given by the manufacturer to compensate for the detector response in the different spectral regions. Data were processed using the Origin-based software FluorEssence (v. 3.9.0.1 - hotfix #6.0, Origin 8.6001). UV/IR grade spectroscopic solvents were purchased from Carl Roth GmbH (Karlsruhe, Germany). Analyte concentrations were kept below 10^−4^ M following the Beer–Lambert behavior. **Nanosecond transient absorption** experiments were performed on an LP980-K spectrometer from Edinburgh Instruments (Livingston, Scotland) equipped with an iCCD detector from Oxford Instruments Andor Technology Ltd (Belfast, Northern Ireland) (DH320T-25F-03-812), a monochromator (STGM325-MA), and a photomultiplier (PMT-LP R928P). The excitation source was a Nd:YAG/YVO4 laser from Ekspla (Vilnius, Lithunia) (NT342B-10-AW) equipped with a tunable OPO (410–2600 nm). Deaerated samples were prepared in the glove box and sealed in quartz cuvettes with air-tight screw caps.

The *4,4′-bipyrimidine* (**bpm**) [66] was synthesized by radical anion coupling following a modified literature procedure optimized for 6,6′-diaryl-substituted analogues by Ioachim et al. [39]. In a typical batch, sodium (517 mg, 22.5 mmol, 6 eq.) was washed in inert *n-*heptane, cut into small pieces and added to a solution of pyrimidine (600 mg, 7.5 mmol, 2 eq.) in dry THF (15 mL) under an argon atmosphere. The surface of the metal chunks turned dark red at once. The suspension was vigorously stirred at room temperature and subjected to ultrasonication several times after half an hour of stirring, resulting in a dark purple solution that was further stirred overnight. The reaction was quenched with absolute ethanol (12 mL) and triethylamine (0.6 mL), and pressurized air was bubbled through the solution for 2 h, upon which it turned yellow. The reaction mixture was extracted into methylene chloride and washed with water three times. The aqueous phase lost its color over time and was thus extracted into methylene chloride again. The combined organic extracts were dried over MgSO_4_ and concentrated under reduced pressure, upon which colorless needles formed. Recrystallization from methanol afforded fine colorless needles (374 mg, 2.4 mmol) in 63% yield. The ^1^H-NMR (400 MHz, CDCl_3_, 25 °C) δ = 9.34 (d, *J* = 1.4 Hz, 2H, H^2^), 8.95 (d, *J* = 5.2 Hz, 2H, H^6^), 8.42 (dd, *J* = 5.2/1.4 Hz, 2H, H^5^) ppm. The ^13^C-NMR (101 MHz, CDCl_3_, 25 °C) δ = 160.78, 159.11, 158.86, 117.96 ppm.

The *4,4′-biquinazoline* (**bqz**) [67] was synthesized following a modified literature procedure by Ucar et al. [40]. In a vacuum-dried 250 mL two-necked Schlenk flask with dropping funnel and cooler, 2,2,6,6-tetramethylpiperidine (TMPH, 2.5 mL, 2.09 g, 15 mmol) was degassed by three freeze-pump-thaw cycles (−78 °C, acetone/ CO_2(s)_). An amount of *n*-BuLi (2.5 M in *n*-hexane, 5.5 mL, 14 mmol) was added via cannula at −10 °C (ice/NaCl), dry THF (40 mL) was added, and the mixture cooled to −78 °C (acetone/CO_2(s)_). Upon stirring for another 15 min, quinazoline (1.50 g, 11.5 mmol) was dissolved into THF (25 mL) inside the dropping funnel and slowly added (90 min) to the in situ generated LiTMP under vigorous stirring (−78 °C), which resulted in a purple color at once. Upon complete addition, the reaction mixture was stirred at −78 °C for another 2 h before slowly being allowed to reach room temperature overnight. The dark red suspension formed was filtered through a glass fritted funnel (Por. 4), which was accompanied by a color change to yellow. Some of the filter cake soluble in methylene chloride was combined with the THF solution, and the solvents were removed under reduced pressure. The crude was subjected to silica column chromatography (CHCl_3_:MeOH 97:3) and recrystallized from EtOAc, which affording a colorless solid (831 mg, 3.2 mmol) in 56% yield. The ^1^H-NMR (400 MHz, CDCl_3_, 25 °C) δ = 9.52 (s, 2H), 8.20 (d, *J* = 8.6 Hz, 2H), 7.97 (ddd, *J* = 8.6/6.9/1.4 Hz, 2H), 7.90 (dd, *J* = 8.4/1.4 Hz, 2H), 7.61 (ddd, *J* = 8.4/6.9/1.4 Hz, 2H) ppm. The ^13^C-NMR (101 MHz, CDCl_3_, 25 °C) δ = 163.63, 154.17, 151.49, 134.72, 129.17, 128.74, 126.53, 123.58 ppm.

The *1,3,10,12-tetraazaperylene* (**1,3,10,12-tape**) was obtained by radical anion coupling [41] of 4,4′-biquinazoline. Potassium chunks in mineral oil were cut into smaller cubes, washed in inert *n*-heptane under argon, and transferred into a vacuum-dried Schlenk flask for storage in the glovebox. In a typical batch, **bqz** (130 mg, 0.50 mmol) was weighed into a capped glass vial and introduced into the glovebox (Ar atmosphere), where 1,2-dimethoxyethane (DME, 4 mL) was added. Upon vigorously stirring the slurry using a glass-coated magnetic stirring bar, potassium (239 mg, 6.0 mmol, 12 eq.) was cut into fine pieces and added. The mixture turned purple at once and was stirred (750 rpm) at room temperature for 70 h. A dark red suspension formed, which was separated from leftover potassium via canula using THF (30 mL) and transferred into a 50 mL Schlenk flask. Oxygen was bubbled through the solution for 1 h, upon which the color of the suspension got darker. It was filtered through a celite plug, where an orange solution was obtained, while a blue solid was left on top of the celite. Chloroform (180 mL) was added to dissolve most of the crude product and the solvents of the combined organic solutions removed under reduced pressure. The celite was washed with water, where the blue solid dissolved and with chloroform (150 mL) again; the organic solution added to the crude product and washed with water (60 mL) three times in a separating funnel. Upon drying over MgSO_4_, the solvent was removed again and the obtained an orange-red solid subjected to silica column chromatography (chloroform:methanol 99:1–97:3). The azaperylene was obtained as yellow-orange needles, typically isolated in 73% yield (94 mg, 0.37 mmol). The ^1^H-NMR (600 MHz, CDCl_3_, 25 °C) δ = 9.62 (s, 2H^1^), 8.30 (dd, *J* = 7.4/1.1 Hz, 2H^4^), 8.06 (dd, *J* = 8.5/1.1 Hz, 2H^2^), 7.99 (dd, *J* = 8.5/7.4 Hz, 2H^3^) ppm. The ^13^C-NMR (151 MHz, CDCl_3_, 25 °C) δ = 157.20 (C^1^), 156.77 (C^5^), 152.12 (C^8^), 134.88 (C^3^), 129.73 (C^2^), 129.60 (C^6^), 122.97 (C^4^), 121.62 (C^7^) ppm. HR-ESI-MS (CHCl_3_) [M] = [C_16_H_8_N_4_]; *theor. calc. for* [M + H^+^]^+^ *^m^*/*_z_* = 257.08217; *exp. found ^m^*/*_z_* = 257.08220.

*[Re(CO)_3_(bpm)Cl]* (**1**): Under an argon atmosphere, a solution of 39.5 mg (0.25 mmol, 1 eq.) **bpm** and 90.4 mg (0.25 mmol, 1 eq.) Re(CO)_5_Cl in 15 mL toluene was heated for 16 h under reflux. The precipitate formed upon cooling in an ice bath was filtered and washed with diethyl ether and *n*-hexane. A concentrated solution of the light red solid in acetonitrile was filtered through a glass pipette with cotton and carefully layered with diethyl ether for precipitation. Complex 1 was obtained in 70.8 mg (0.15 mmol, 61%) yield. Crystals suitable for scXRD analysis were obtained by slow diffusion of *n*-hexane into a concentrated chloroform solution. The ^1^H-NMR (600 MHz, CDCl_3_, 25 °C) δ = 9.79 (d, 1.2 Hz, H^2^, 2H), 9.23 (d, 5.1 Hz, H^5^, 2H), 8.22 (dd, 5.1/1.2 Hz, H^6^, 2H) ppm. ^1^H-NMR (600 MHz, CD_3_CN, 25 °C) δ = 9.70 (d, 1.3 Hz, H^2^, 2H), 9.25 (d, 5.3 Hz, H^5^, 2H), 8.50 (dd, 5.3/1.3 Hz, H^6^, 2H) ppm. The ^13^C-NMR (151 MHz, CD_3_CN, RT, UDEFT) δ = 197.61, 189.13, 162.18, 161.98, 161.95, 121.53 ppm. HR-MALDI-MS (DCTB) [M] = [C_11_H_6_ClN_4_O_3_Re]; *theor. calc. for* [M+e^-^]^-^ *^m^*/*_z_* = 463.96859; *exp. found ^m^*/*_z_* = 463.96946. HR-ESI-MS (CH_3_CN) [M] = [C_11_H_6_ClN_4_O_3_Re]; *theor. calc. for* [M-Cl^−^+CH_3_CN]^+^ *^m^*/*_z_* = 470.02626; *exp. found ^m^*/*_z_* = 470.02526.

*[Re(CO)_3_(bqz)Cl]* (**2**): Under an argon atmosphere, a solution of 31.4 mg (0.12 mmol, 1 eq.) **bqz** and 44.0 mg (0.12 mmol, 1 eq.) Re(CO)_5_Cl in 10 mL toluene was heated for 16 h under reflux. Upon cooling to RT, the solvent was removed under reduced pressure, and the residue successively dispersed in diethyl ether and *n*-hexane was collected by filtration. The purple solid was redissolved into warm chloroform and carefully layered with *n*-hexane for precipitation. Complex **2** was obtained in 44.6 mg (0.08 mmol, 66%) yield. The ^1^H-NMR (600 MHz, CD_3_CN, 25 °C) δ = 9.79 (s, 1H), 9.76 (s, 1H), 8.39 (d, 8.6 Hz, 1H), 8.36 (d, 8.5 Hz, 1H), 8.17 (m, 2H), 8.02 (m, 2H), 7.78 (m, 2H) ppm. The ^13^C-NMR (151 MHz, CD_3_CN, RT, UDEFT) δ = 196.23, 195.80, 187.16, 163.76, 163.23, 155.56, 155.32, 152.71, 151.96, 137.10, 136.88, 130.23, 130.13, 129.97, 126.92, 124.62, 124.03 ppm. HR-ESI-MS (CH_3_CN) [M] = [C_19_H_10_ClN_4_O_3_Re]; *theor. calc. for* [M-Cl^−^+CH_3_CN]^+^ *^m^*/*_z_* = 570.05759; *exp. found ^m^*/*_z_* = 570.05800.

*[Re(CO)_3_(1,3,10,12-tape)Cl]* (**3**): Under an argon atmosphere, a solution of 30.8 mg (0.12 mmol, 1 eq.) **1,3,10,12-tape** and 43.4 mg (0.12 mmol, 1 eq.) Re(CO)_5_Cl in 12 mL toluene was heated for 16 h under reflux. Upon cooling to RT, the solvent was removed under reduced pressure; the residue successively dispersed in diethyl ether and *n*-hexane was collected by filtration. The dark purple solid was redissolved into warm chloroform and carefully layered with *n*-hexane. Complex **3** was obtained as fine needles in 52.0 mg (0.09 mmol, 77%) yield. Crystals suitable for scXRD analysis were obtained by slow diffusion of *n*-hexane into a concentrated chloroform solution. The ^1^H-NMR (600 MHz, CD_3_CN, 25 °C) δ = 9.72 (s, 2H^1^), 8.56 (d, 7.3 Hz, 2H^4^), 8.27 (d, 8.4 Hz, 2H^2^), 8.24 (dd, 8.4/7.3 Hz, 2H^3^) ppm. The ^13^C-NMR (151 MHz, CD_3_CN, RT) δ = 195.85, 159.26, 156.26, 151.07, 137.58, 130.08, 129.46, 125.78, 120.89, 116.50 ppm. HR-ESI-MS (CH_3_CN) [M] = [C_19_H_8_ClN_4_O_3_Re]; *theor. calc. for* [M-Cl^−^+CH_3_CN]^+^ *^m^*/*_z_* = 568.04194; *exp. found ^m^*/*_z_* = 568.04503.

## Figures and Tables

**Table 1:** Cyclic voltammetry data for reduction of the complexes [Re(CO)_3_(NN)Cl] (**1**–**3**) and the free bidentate diimine ligands ^a^.

Compound	$E_{1/2}^{\left(0/-\right)}$/V vs. FC^+^/Fc (ΔE/mV)	$E_{pc}^{\left(-/2-\right)}$/V vs. FC^+^/Fc	$E_{pc}^{\left(2-/3-\right)}$/V vs. FC^+^/Fc
**bpm** ^b^	−1.863 (70)	−2.554	-
**bqz**	−1.609 (72)	−2.043	-
**1,3,10,12-tape**	−1.131 (64)	−1.771	-
[Re(CO)_3_(**bpm**)Cl] (**1**) ^c^	−1.095 (70)	−1.695	-
[Re(CO)_3_(**bqz**)Cl] (**2**)	−0.738 (66)	−1.283	-
[Re(CO)_3_(**1,3,10,12-tape**)Cl] (**3**)	−0.523 (66)	−1.164	−2.702

^a^ Halfwave potentials ($E_{1/2}^{\left(0/-\right)}$) and peak potentials ($E_{pc}^{\left(-/2-\right)},\;E_{pc}^{\left(2-/3-\right)}$) are given vs. Fc^+^/Fc. Potentials were measured vs. a non-aqueous AgNO_3_/Ag reference electrode (0.1 M *n*Bu_4_NPF_6_, 0.01 M AgNO_3_ in MeCN) and referenced to the Fc^+^/Fc couple; 0.1 M *n*Bu_4_NPF_6_, DMF as electrolyte solution, 1 mM analyte concentration, 100 mV s^−1^; glassy carbon working electrode; platinum wire counter electrode. ΔE peak separation for reversible first reduction; a peak separation of 64–84 mV was found for Fc^+^/Fc; E_pc_ cathodic peak potential for irreversible process at 100 mV s^−1^ scan rate. For ^b^, see also [48]. For ^c^, see also [46].

**Table 2:** Photophysical data for the Re(CO)_3_(NN)Cl complexes in CHCl_3_ and CH_3_CN along with some literature-reported Re(I)-based analogous complexes.

Compound	CHCl_3_		CH_3_CN	
	$\lambda_{\mathrm{MLCT}}$/nm (ε_λ_/M^−1^ cm^−1^)	$\lambda_{\pi -\pi *}$/nm (ε_λ_/M^−1^ cm^−1^)	$\lambda_{\mathrm{MLCT}}$/nm (ε_λ_/M^−1^ cm^−1^)	$\lambda_{\pi -\pi *}$/nm (ε_λ_/M^−1^ cm^−1^)
**bpm**	-	271 (13,995)	-	-
**bqz**	-	320 (7660)	-	-
**1,3,10,12-tape**	-	414 (sh) 440 (11,525) 467 (10,400)	-	-
**1**	467 (2910)	283 (16,610)	427 (2700)	279 (15,325)
**2**	532 (4160)	378 (7155)	491 (3320)	378 (5730)
**3 ^a^**	586 (5450)	447 (sh) 480 (7900) 512 (9290)	~550 ^b^	444 (sh) 476 506
[Re(CO)_3_(2,2′-bpy)Cl] ^c^	384	-	370 (3420)	-
[Re(CO)_3_(1,10-phen)Cl] ^c^	374	-	364 (3350)	-
[Re(CO)_3_(3,3′-bipyridazine)Cl] ^d^	463 (3649)	363	-	-
[Re(**dpb**)(CO)_3_Cl] ^e^	464 (2380)	-	-	-
[Re(**dmpb**)(CO)_3_Cl] ^e^	460 (790)	-	-	-
[Re(**dnb**)(CO)_3_Cl] ^e^	431 (2790)	-	-	-

^a^ **3** is poorly soluble in CH_3_CN, qualitative data is represented here, ^b^ due to overlap of MLCT band with the ILCT band in CH_3_CN, the absorption maximum could not be measured, ^c^ from ref. [52], ^d^ from ref. [29], ^e^ from ref. [35], **dpb** = 6,6′-diphenyl-, **dmpb** = 6,6′-*p-*methoxyphenyl-, **dnb** = 6,6′-(1-naphythyl)-4,4′-bipyrimidine.

**Table 3:** Electronic absorption maxima of ligands (L) **bpm**, **1,3,10,12-tape**, and Re(CO)_3_(NN)Cl complexes **1**–**3** and of their electrochemically reduced forms from UV–Vis NIR spectroelectrochemistry in DMF/0.1 M *n*Bu_4_NPF_6_ ^a^.

Compound	L	L^●−^	L^2−^
	$\lambda_{\max}$/nm	$\lambda_{\max}$/nm	$\lambda_{\max}$/nm
**bpm** ^b^	272, 318 (br)	350 (sh), 362, 445 (sh), 468 (sh), 496, 530, 603, 660, 840, 950, 1100	363
**1,3,10,12-tape**	278, 319 (sh), 331, 439, 458 (sh)	292, 311 (sh), 380, 406, 470 (sh), 502, 626, 670, 683 (sh), 740 (sh), 755	271, 296, 342 (sh), 407, 490 (sh), 513
**1**	278, 424 (br)	328, 345, 435 (sh), 456, 486, 610, 680, 755	355
**2**	271, 328 (sh), 378, 388, 489	268, 293, 348 (sh), 392, 468, 526 (sh), 718, 768 (sh)	271, 305 (sh), 365 (sh), 518
**3**	268, 388, 482 (sh), 509, 555 (sh), 620 (br)	298, 336, 354, 400, 472, 530 (sh), 560 (sh), 633, 703, 722, (sh), 780 (sh), 803	294, 345, 407, 484, 506 (sh), 563, 612

^a^ Most intense components are underlined. ^b^ From reference [59].

## Data Availability

CCDC 2225447 and 2225446 contain the supplementary crystallographic data for this paper. These data can be obtained free of charge from The Cambridge Crystallographic Data Centre via www.ccdc.cam.ac.uk/data_request/cif (accessed on 22 December 2022).

## Supplementary Materials

The following supporting information can be downloaded at: https://www.mdpi.com/article/10.3390/molecules28041905/s1, Figures S1–S24: NMR characterization of compounds; Figures S25–S29: HR-MS characterization of compounds; Figures S30–S32: ATR-IR characterization of compounds; Figures S33–S38: Crystallographic details on compounds; Figure S39: Cyclic voltammograms showing further reduction events; Figure S40: Cyclic voltammograms of compound **3** at variable scan rate; Figures S41–S49: Photophysical characterization of compounds; Figures S50–S58: UV–Vis NIR spectroelectrochemical characterization of compounds; Figures S59–S61: Nanosecond transient absorption spectra of the complexes; Tables S1–S4: Crystallographic details. CCDC 2225447 and 2225446 contain the supplementary crystallographic data for this paper.

## Sample Availability

Samples of the compounds 4,4’-bipyrimdine (bpm), 4,4’-biquinazoline (bqz), 1,3,10,12-tetraazaperylene (1,3,10,12-tape), [Re(CO)_3_(bpm)Cl], [Re(CO)_3_(bqz)Cl] and Re(CO)_3_(1,3,10,12-tape)Cl] are available from the authors upon request.

## References

[1] Esswein A.J., Nocera D.G. (2007). Hydrogen Production by Molecular Photocatalysis. Chem. Rev..

[2] Balzani V., Credi A., Venturi M. (2008). Photochemical Conversion of Solar Energy. ChemSusChem.

[3] Teets T.S., Nocera D.G. (2011). Photocatalytic Hydrogen Production. Chem. Commun..

[4] Ceroni P., Balzani V., Ceroni P. (2012). Photoinduced Energy and Electron Transfer Processes. The Exploration of Supramolecular Systems and Nanostructures by Photochemical Techniques.

[5] Monro S., Colón K.L., Yin H., Roque J., Konda P., Gujar S., Thummel R.P., Lilge L., Cameron C.G., McFarland S.A. (2019). Transition Metal Complexes and Photodynamic Therapy from a Tumor-Centered Approach: Challenges, Opportunities, and Highlights from the Development of TLD1433. Chem. Rev..

[6] McFarland S.A., Mandel A., Dumoulin-White R., Gasser G. (2020). Metal-Based Photosensitizers for Photodynamic Therapy: The Future of Multimodal Oncology?. Curr. Opin. Chem. Biol..

[7] Lo K.K.-W. (2015). Luminescent Rhenium(I) and Iridium(III) Polypyridine Complexes as Biological Probes, Imaging Reagents, and Photocytotoxic Agents. Acc. Chem. Res..

[8] Lee L.C.-C., Leung K.-K., Lo K.K.-W. (2017). Recent Development of Luminescent Rhenium(I) Tricarbonyl Polypyridine Complexes as Cellular Imaging Reagents, Anticancer Drugs, and Antibacterial Agents. Dalton Trans..

[9] Takeda H., Ishitani O. (2010). Development of Efficient Photocatalytic Systems for CO_2_ Reduction Using Mononuclear and Multinuclear Metal Complexes Based on Mechanistic Studies. Coord. Chem. Rev..

[10] Takeda H., Koike K., Morimoto T., Inumaru H., Ishitani O. (2011). Photochemistry and Photocatalysis of Rhenium(I) Diimine Complexes. Advances in Inorganic Chemistry.

[11] Rau S., Görls H. (2004). Solid State Structure of a Rhenium Bibenzimidazole Complex. J. Coord. Chem..

[12] Gottschaldt M., Koth D., Müller D., Klette I., Rau S., Görls H., Schäfer B., Baum R.P., Yano S. (2007). Synthesis and Structure of Novel Sugar-Substituted Bipyridine Complexes of Rhenium and 99m-Technetium. Chem. Eur. J..

[13] Braumüller M., Schulz M., Staniszewska M., Sorsche D., Wunderlin M., Popp J., Guthmuller J., Dietzek B., Rau S. (2016). Synthesis and Characterization of Ruthenium and Rhenium Dyes with Phosphonate Anchoring Groups. Dalton Trans..

[14] Probst B., Kolano C., Hamm P., Alberto R. (2009). An Efficient Homogeneous Intermolecular Rhenium-Based Photocatalytic System for the Production of H_2_. Inorg. Chem..

[15] Probst B., Rodenberg A., Guttentag M., Hamm P., Alberto R. (2010). A Highly Stable Rhenium−Cobalt System for Photocatalytic H_2_ Production: Unraveling the Performance-Limiting Steps. Inorg. Chem..

[16] Probst B., Guttentag M., Rodenberg A., Hamm P., Alberto R. (2011). Photocatalytic H_2_ Production from Water with Rhenium and Cobalt Complexes. Inorg. Chem..

[17] Guttentag M., Rodenberg A., Kopelent R., Probst B., Buchwalder C., Brandstätter M., Hamm P., Alberto R. (2012). Photocatalytic H_2_ Production with a Rhenium/Cobalt System in Water under Acidic Conditions. Eur. J. Inorg. Chem..

[18] Abram U., Alberto R. (2006). Technetium and Rhenium: Coordination Chemistry and Nuclear Medical Applications. J. Braz. Chem. Soc..

[19] Alberto R., Meola G., Valdés D.H. (2019). Technetium and Rhenium Complexes with Aromatic Hydrocarbons as Ligands. Advances in Bioorganometallic Chemistry.

[20] Alberto R., Braband H., Nadeem Q. (2020). Bioorganometallic Technetium and Rhenium Chemistry: Fundamentals for Applications. Chimia.

[21] Howell S.L., Scott S.M., Flood A.H., Gordon K.C. (2005). The Effect of Reduction on Rhenium(I) Complexes with Binaphthyridine and Biquinoline Ligands: A Spectroscopic and Computational Study. J. Phys. Chem. A.

[22] Yi X., Zhao J., Wu W., Huang D., Ji S., Sun J. (2012). Rhenium(I) Tricarbonyl Polypyridine Complexes Showing Strong Absorption of Visible Light and Long-Lived Triplet Excited States as a Triplet Photosensitizer for Triplet–Triplet Annihilation Upconversion. Dalton Trans..

[23] Qiao X., Li Q., Schaugaard R.N., Noffke B.W., Liu Y., Li D., Liu L., Raghavachari K., Li L. (2017). Well-Defined Nanographene–Rhenium Complex as an Efficient Electrocatalyst and Photocatalyst for Selective CO_2_ Reduction. J. Am. Chem. Soc..

[24] Hino J.K., Della Ciana L., Dressick W.J., Sullivan B.P. (1992). Substituent Constant Correlations as Predictors of Spectroscopic, Electrochemical, and Photophysical Properties in Ring-Substituted 2,2′-Bipyridine Complexes of Rhenium(I). Inorg. Chem..

[25] Wrighton M., Morse D.L. (1974). Nature of the Lowest Excited State in Tricarbonylchloro-1,10-Phenanthrolinerhenium(I) and Related Complexes. J. Am. Chem. Soc..

[26] Komreddy V., Ensz K., Nguyen H., Paul Rillema D. (2020). Synthesis and Characterization of Rhenium(I) 4,4′-Dicarboxy-2,2′-Bipyridine Tricarbonyl Complexes for Solar Energy Conversion. Inorg. Chim. Acta.

[27] Artem’ev A.V., Petyuk M.Y., Berezin A.S., Gushchin A.L., Sokolov M.N., Bagryanskaya I.Y. (2021). Synthesis and Study of Re(I) Tricarbonyl Complexes Based on Octachloro-1,10-Phenanthroline: Towards Deep Red-to-NIR Emitters. Polyhedron.

[28] Ernst S., Kaim W. (1986). Coordination Characteristics of Four Isomeric .Alpha.-Diimine Ligands. .Pi. Molecular Orbital Perturbation Calculations for the Bidiazines and Their Correlation with the Properties of Group 6 Metal Carbonyl Complexes. J. Am. Chem. Soc..

[29] Kaim W., Kramer H.E.A., Vogler C., Rieker J. (1989). Synthesis, Electrochemistry and Emission Spectroscopy in Fluid Solution of Four Isomeric (α-Diimine)Re(CO)_3_Hal Complexes. J. Organomet. Chem..

[30] Knör G., Leirer M., Keyes T.E., Vos J.G. (2000). Non-Luminescent 1,2-Diiminetricarbonylrhenium(I) Chloride Complexes–Synthesis, Electrochemical and Spectroscopic Properties of Re(DIAN)(CO)_3_Cl with DIAN =p-Substituted Bis(Arylimino)Acenaphthene. Eur. J. Inorg. Chem..

[31] Schnierle M., Winkler M., Filippou V., Slageren J., Ringenberg M.R. (2022). (Spectro)Electrochemistry of 3-(Pyrid-2-yl)-s-Tetrazine- and 1,2- (Dihydro)Pyridazine Tricarbonylrhenium(I)Chloride. Euro. J. Inorg. Chem..

[32] Chakrabortty S., Agrawalla B.K., Stumper A., Vegi N.M., Fischer S., Reichardt C., Kögler M., Dietzek B., Feuring-Buske M., Buske C. (2017). Mitochondria Targeted Protein-Ruthenium Photosensitizer for Efficient Photodynamic Applications. J. Am. Chem. Soc..

[33] Loftus L.M., Al-Afyouni K.F., Turro C. (2018). New Ru^II^ Scaffold for Photoinduced Ligand Release with Red Light in the Photodynamic Therapy (PDT) Window. Chem. Eur. J..

[34] Chen Q., Dan H., Tang F., Wang J., Li X., Cheng J., Zhao H., Zeng X. (2019). Photodynamic Therapy Guidelines for the Management of Oral Leucoplakia. Int. J. Oral Sci..

[35] Ioachim E., Medlycott E.A., Hanan G.S. (2006). Synthesis and Properties of Re(I) Tricarbonyl Complexes of 6,6′-Disubstituted-4,4′-Bipyrimidines with High Energy Excited States Suitable for Incorporation into Polynuclear Arrays. Inorg. Chim. Acta.

[36] Mosberger M., Probst B., Spingler B., Alberto R. (2019). Influence of Hetero-Biaryl Ligands on the Photo-Electrochemical Properties of [Re^I^NCS(N^∩^N)(CO)_3_]-Type Photosensitizers. Eur. J. Inorg. Chem..

[37] Ernst S., Kaim W. (1985). D6-Metal Complexes of 4,4?-Bipyrimidine, an Ambident Ligand with High?-Acceptor Ability. Angew. Chem. Int. Ed. Engl..

[38] Ademi L., Constable E.C., Housecroft C.E., Neuburger M., Schaffner S. (2003). Spontaneous Resolution of a Diastereomeric Ruthenium(II) Complex with an Atropisomeric 4,4′-Biquinazoline Ligand. Dalton Trans..

[39] Ioachim E., Medlycott E.A., Polson M.I.J., Hanan G.S. (2005). Synthesis of a Novel Series of 6,6′-Disubstituted 4,4′-Bipyrimidines by Radical Anion Coupling: New π-Accepting Ligands for Coordination Chemistry. Eur. J. Org. Chem..

[40] Ucar S., Dastan A. (2020). One-Pot Homo- and Cross-Coupling of Diazanaphthalenes via C-H Substitution: Synthesis of Bis- and Tris-Diazanaphthalenes. J. Heterocycl. Chem..

[41] Schmelz O., Mews A., Basché T., Herrmann A., Müllen K. (2001). Supramolecular Complexes from CdSe Nanocrystals and Organic Fluorophors. Langmuir.

[42] Brietzke T., Mickler W., Kelling A., Holdt H.-J. (2012). Mono- and Dinuclear Ruthenium(Ii) 1,6,7,12-Tetraazaperylene Complexes. Dalton Trans..

[43] Hirono A., Sakai H., Kochi S., Sato T., Hasobe T. (2020). Electrochemical Properties and Excited-State Dynamics of Azaperylene Derivatives. J. Phys. Chem. B.

[44] Grunwald N., Schilde U., Kelling A., Holdt H.-J. CCDC 631826: Experimental Crystal Structure Determination Cambridge Crystallographic Data Centre: Cambridge, England, 2016.

[45] Fitchett C.M., Richardson C., Steel P.J. (2005). Solid State Conformations of Symmetrical Aromatic Biheterocycles: An X-Ray Crystallographic Investigation. Org. Biomol. Chem..

[46] Klein A., Vogler C., Kaim W. (1996). The δ in 18 + δ Electron Complexes: Importance of the Metal/Ligand Interface for the Substitutional Reactivity of “Re(0)” Complexes (α-Diimine^-^)Re^I^(CO)_3_(X). Organometallics.

[47] Kaim W., Ernst S. (1986). ESR and ENDOR Study of Three Isomeric Bidiazine Anion Radicals and of Their Group 6 Metal Carbonyl Complexes. Coordinative Effects on the Spin Distribution. J. Phys. Chem..

[48] Ladwig M., Kaim W. (1991). Spectroscopic and Electrochemical Properties of the Isomeric Bidiazine Complexes [(C_5_Me_5_)ClRh(Bdz)]+ and (C_5_Me_5_)Rh(Bdz) and Their Relevance to the Catalysis of the 2 H^+^ → H_2_ Reaction by 2,2′-Bipyridine Analogues. J. Organomet. Chem..

[49] Zoric M.R., Askins E.J., Qiao X., Glusac K.D. (2021). Strong Electronic Coupling of Graphene Nanoribbons onto Basal Plane of a Glassy Carbon Electrode. ACS Appl. Electron. Mater..

[50] Xiong T., Włodarczyk R., Saalfrank P. (2018). Vibrationally Resolved Absorption and Fluorescence Spectra of Perylene and N-Substituted Derivatives from Autocorrelation Function Approaches. Chem. Phys..

[51] Jacobi R., Hernández-Castillo D., Sinambela N., Bösking J., Pannwitz A., González L. (2022). Computation of Förster Resonance Energy Transfer in Lipid Bilayer Membranes. J. Phys. Chem. A.

[52] Kalyanasundaram K. (1986). Luminescence and Redox Reactions of the Metal-to-Ligand Charge-Transfer Excited State of Tricarbonylchloro-(Polypyridyl)Rhenium(I) Complexes. J. Chem. Soc. Faraday Trans. 2.

[53] Juris A., Balzani V., Barigelletti F., Campagna S., Belser P., von Zelewsky A. (1988). Ru(II) Polypyridine Complexes: Photophysics, Photochemistry, Eletrochemistry, and Chemiluminescence. Coord. Chem. Rev..

[54] Saldías M., Guzmán N., Palominos F., Sandoval-Altamirano C., Günther G., Pizarro N., Vega A. (2019). Electronic and Photophysical Properties of Re^I^(CO)_3_Br Complexes Modulated by Pyrazolyl–Pyridazine Ligands. ACS Omega.

[55] Manuta D.M., Lees A.J. (1983). Solvent and Substituent Effects on the Lowest Energy Excited States of M(CO)_4_(Diimine) (M = Cr, Mo, W) Complexes. Inorg. Chem..

[56] Hori H., Ishihara J., Koike K., Takeuchi K., Ibusuki T., Ishitani O. (1999). Photocatalytic Reduction of Carbon Dioxide Using [Fac-Re(Bpy)(CO)_3_(4-Xpy)]^+^ (Xpy=pyridine Derivatives). J. Photochem. Photobiol. A Chem..

[57] Rodríguez L., Ferrer M., Rossell O., Duarte F.J.S., Gil Santos A., Lima J.C. (2009). Solvent Effects on the Absorption and Emission of [Re(R_2_Bpy)(CO)_3_X] Complexes and Their Sensitivity to CO_2_ in Solution. J. Photochem. Photobiol. A Chem..

[58] Manuta D.M., Lees A.J. (1986). Solvatochromism of the Metal to Ligand Charge-Transfer Transitions of Zero-Valent Tungsten Carbonyl Complexes. Inorg. Chem..

[59] Krejčík M., Záliš S., Ladwig M., Matheis W., Kaim W. (1992). A Study of the Reduced States of Four Isomeric Bidiazines (Diaza-2,2′-Bipyridines) by UV–VIS/NIR-Spectroelectrochemistry. J. Chem. Soc. Perkin Trans. 2.

[60] Ferguson J. (1966). Absorption and Emission Spectra of the Perylene Dimer. J. Chem. Phys..

[61] Portenkirchner E., Kianfar E., Sariciftci N.S., Knör G. (2014). Two-Electron Carbon Dioxide Reduction Catalyzed by Rhenium(I) Bis(Imino)Acenaphthene Carbonyl Complexes. ChemSusChem.

[62] Wähler K., Ludewig A., Szabo P., Harms K., Meggers E. (2014). Rhenium Complexes with Red-Light-Induced Anticancer Activity. Eur. J. Inorg. Chem..

[63] Sheldrick G.M. (2008). A Short History of *SHELX*. Acta Crystallogr. A Found Crystallogr..

[64] Sheldrick G.M. (2015). Crystal Structure Refinement with *SHELXL*. Acta Crystallogr. C Struct. Chem..

[65] Macrae C.F., Edgington P.R., McCabe P., Pidcock E., Shields G.P., Taylor R., Towler M., van de Streek J. (2006). *Mercury*: Visualization and Analysis of Crystal Structures. J. Appl. Crystallogr..

[66] Effenberger F. (1965). Enoläther, II: Synthese und Reaktionen von 1.4-Bis-äthoxymethylen-butandion-(2.3). Chem. Ber..

[67] Armarego W.L.F., Willette R.E. (1965). 220. Quinazolines. Part VI. 2,2′-And 4,4′-Biquinazolinyls. J. Chem. Soc..

