# Supplementary material for: Red Light Absorption of [ReI(CO)3(α-diimine)Cl] Complexes through Extension of the 4,4′-Bipyrimidine Ligand’s π-System
Source: Molecules. 2023 Feb 16;28(4):1905. doi: 10.3390/molecules28041905 (PMC9964139; doi:10.3390/molecules28041905)
Supplement: Supplementary file 1 [file molecules-28-01905-s001.zip › molecules-2149216-supplementary.pdf]

molecules

Supplementary Materials

Red-light absorption of [Re^{I}(CO)$_3$(α-diimine)Cl] complexes through extension of the 4,4′-bipyrimidine ligand's π-system

Nicolas Meitinger [1], Subrata Mandal [1], Dieter Sorsche [1], Andrea Pannwitz [1*] and Sven Rau [1,*]

1 Ulm University, Institute for Inorganic Chemistry I, Albert-Einstein-Allee 11, 89081 Ulm, Germany
* Correspondence: A. P. andrea.pannwitz@uni-ulm.de; S. R. sven.rau@uni-ulm.de

Table of contents

1. **^{1}H-, ^{13}C- and (hetero)dinuclear 2D NMR spectra……………………………………………………………3**
2. **High resolution mass spectra……………………………………………………………………………..15**
3. **ATR-IR transmission spectra……………………………………………………………………………..20**
4. **Crystallographic information……………………………………………………………………………..23**
5. **Electrochemical characterization………………………………………………………………………….30**
6. **UV/Vis-NIR absorption and emission spectra…………………………………………………………….31**
7. **UV-Vis-NIR spectroelectrochemical measurements…………………………………………………...36**
8. **Nanosecond transient absorption spectra………………………………………………………………..41**

1. ^{1}H-, ^{13}C- and (hetero)dinuclear 2D NMR spectra

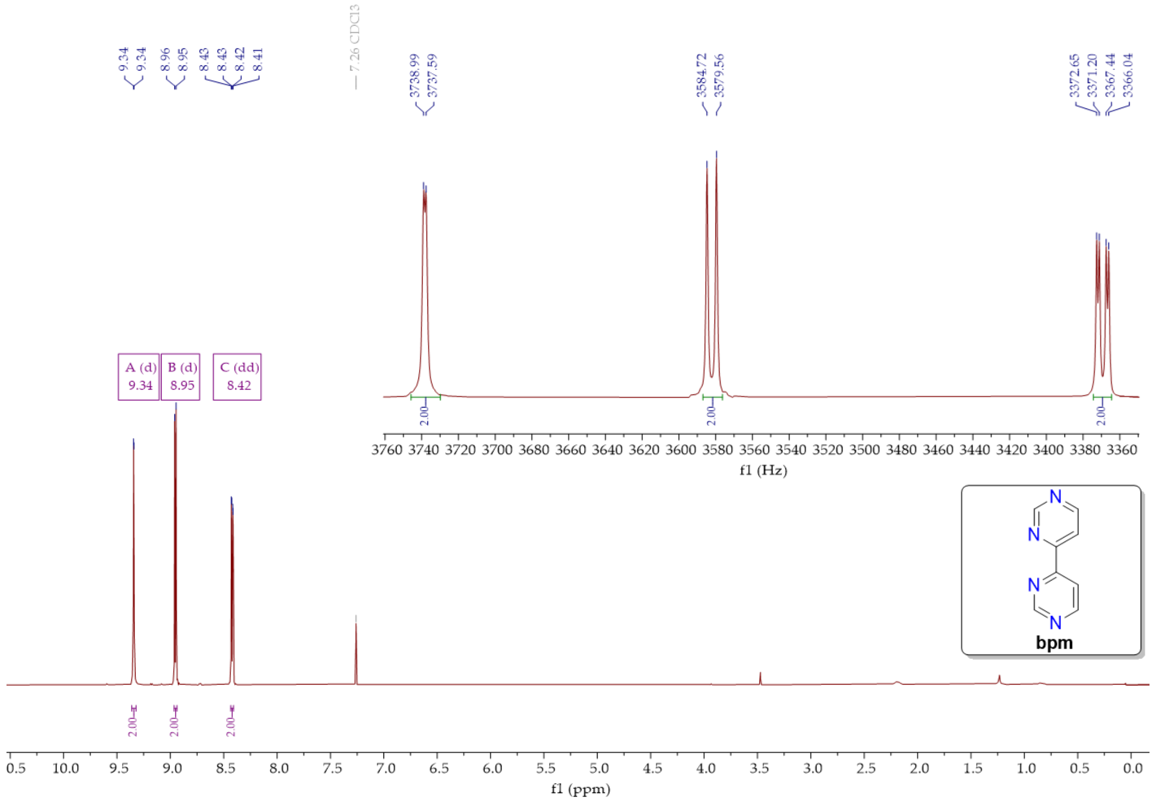

Figure S1: ^{1}H-NMR spectrum (400 MHz, RT, $CDCl_3$) of 4,4′-bipyrimidine (**bpm**).

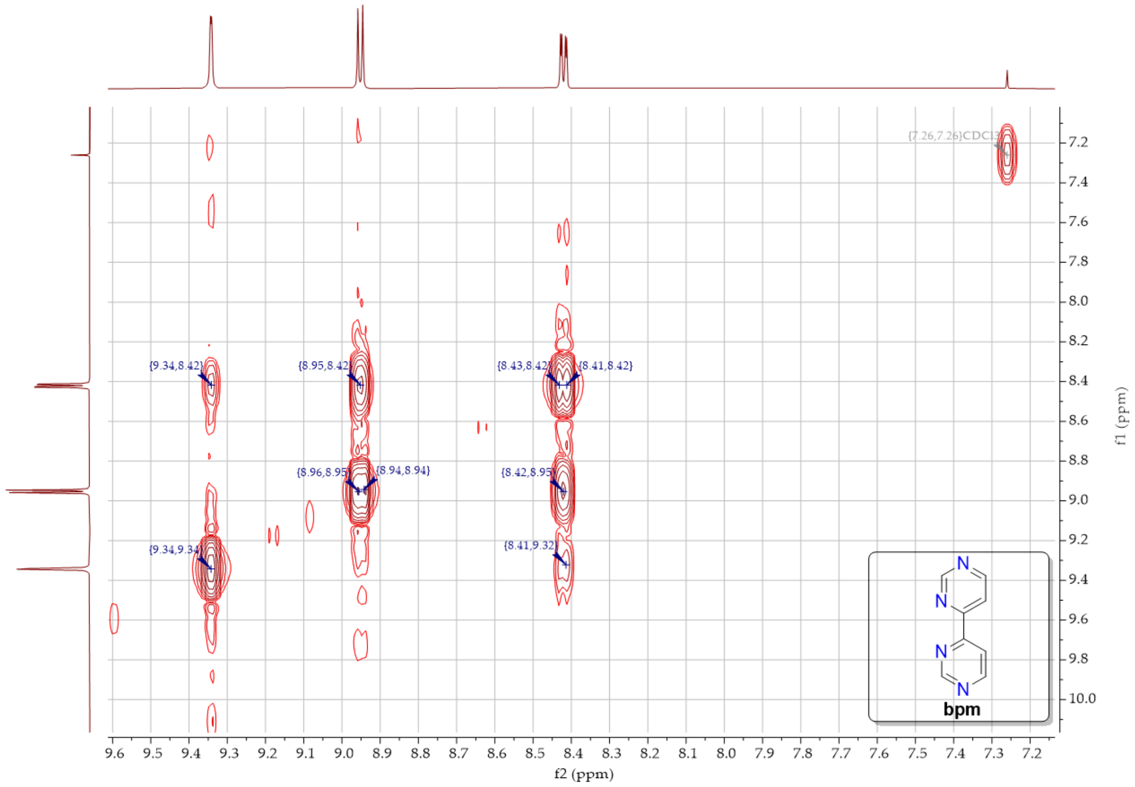

Figure S2: H,H-COSY45 NMR spectrum (400 MHz, RT, $CDCl_3$) of 4,4′-bipyrimidine (**bpm**).

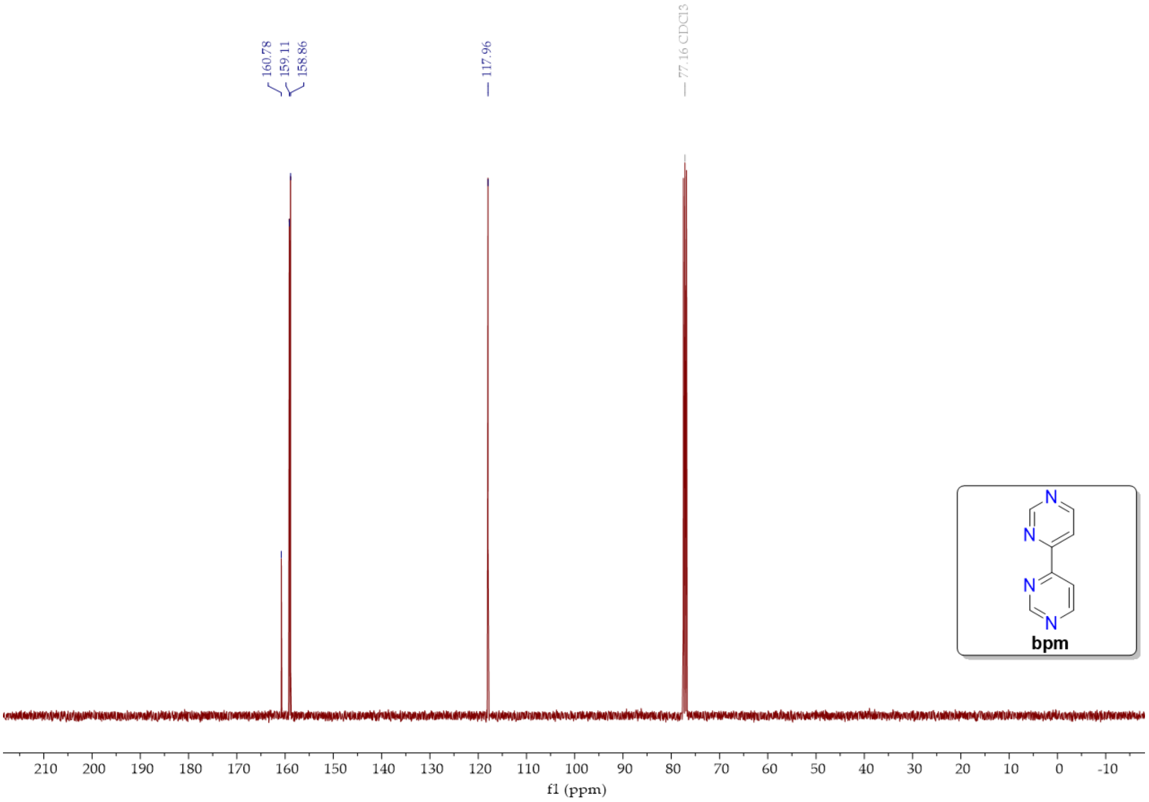

Figure S3: ^{13}C-NMR spectrum (101 MHz, RT, $CDCl_3$) of 4,4′-bipyrimidine (**bpm**).

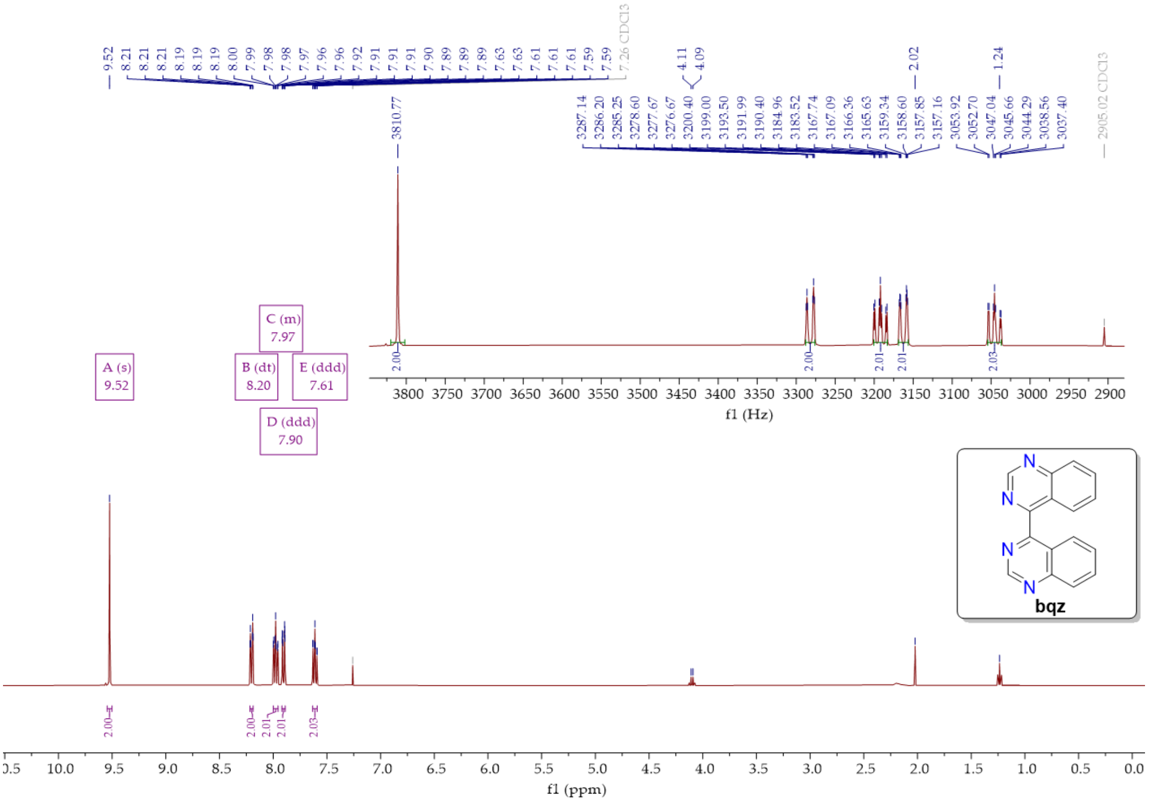

Figure S4: ^{1}H-NMR spectrum (400 MHz, RT, $CDCl_3$) of 4,4′-biquinazoline (**bqz**).

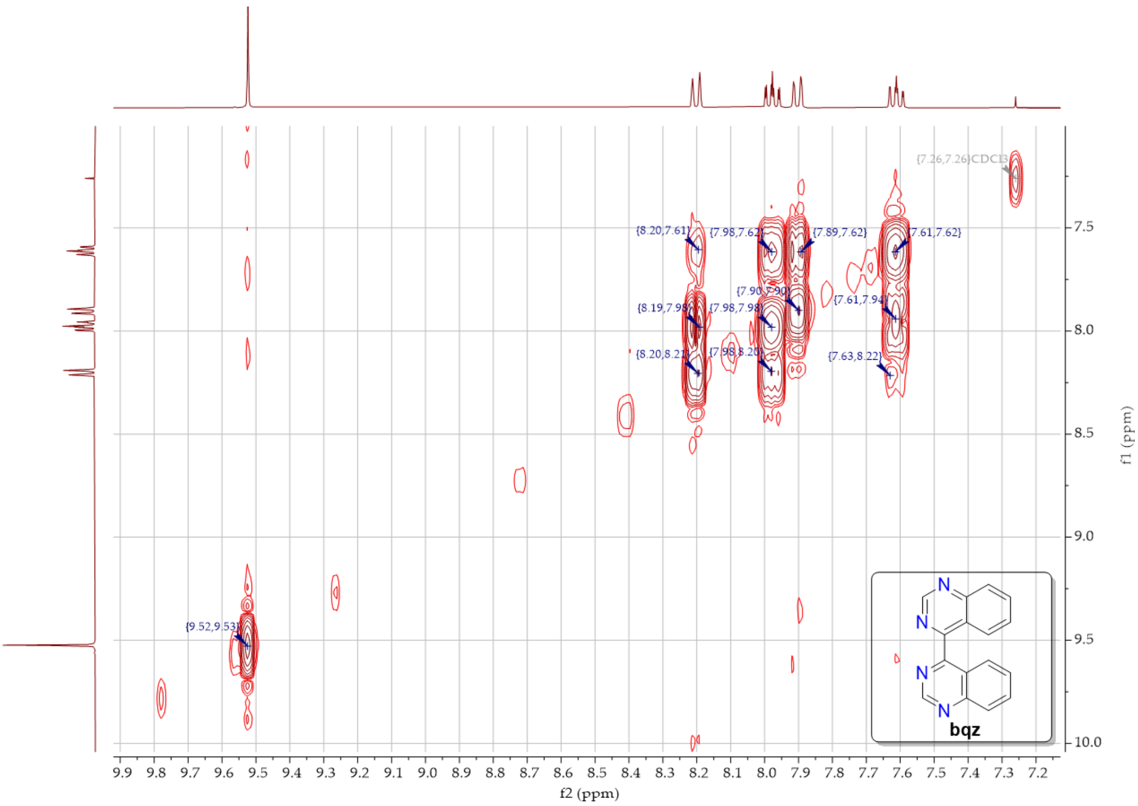

Figure S5: H,H-COSY45 NMR spectrum (400 MHz, RT, $CDCl_3$) of 4,4′-biquinazoline (**bqz**).

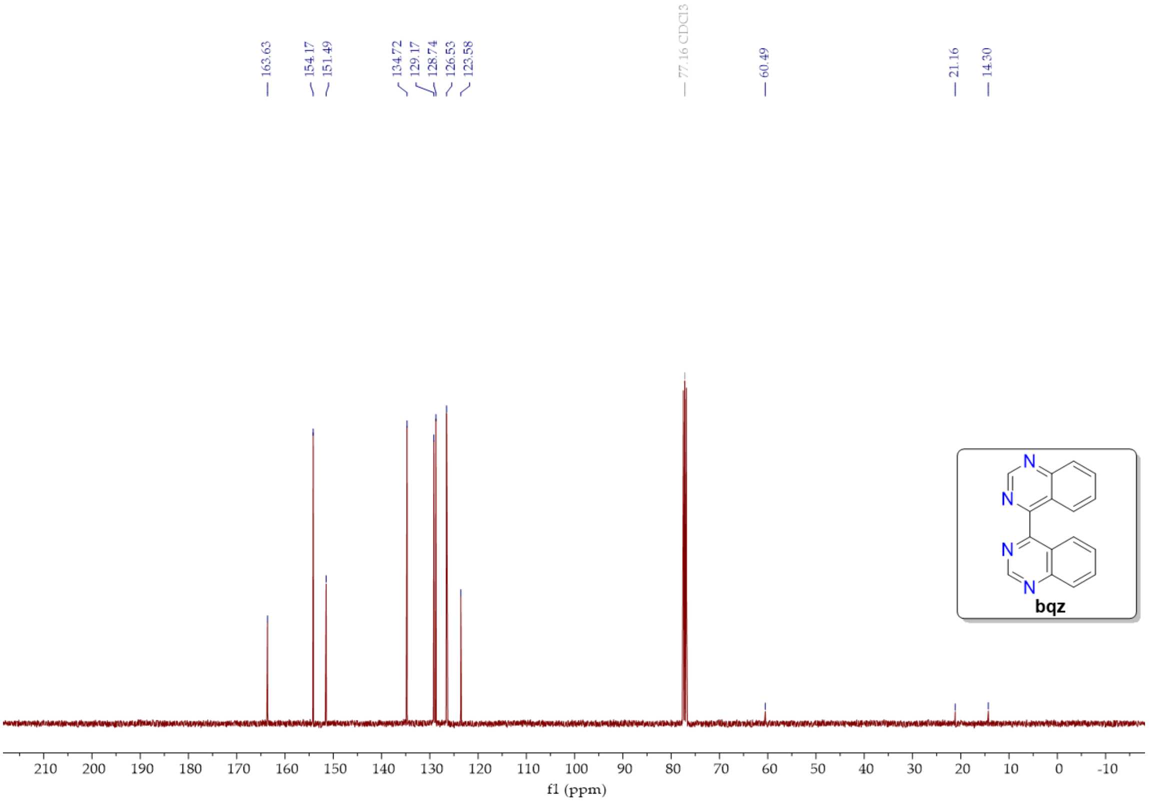

Figure S6: ^{13}C-NMR spectrum (101 MHz, RT, $CDCl_3$) of 4,4′-biquinazoline (**bqz**).

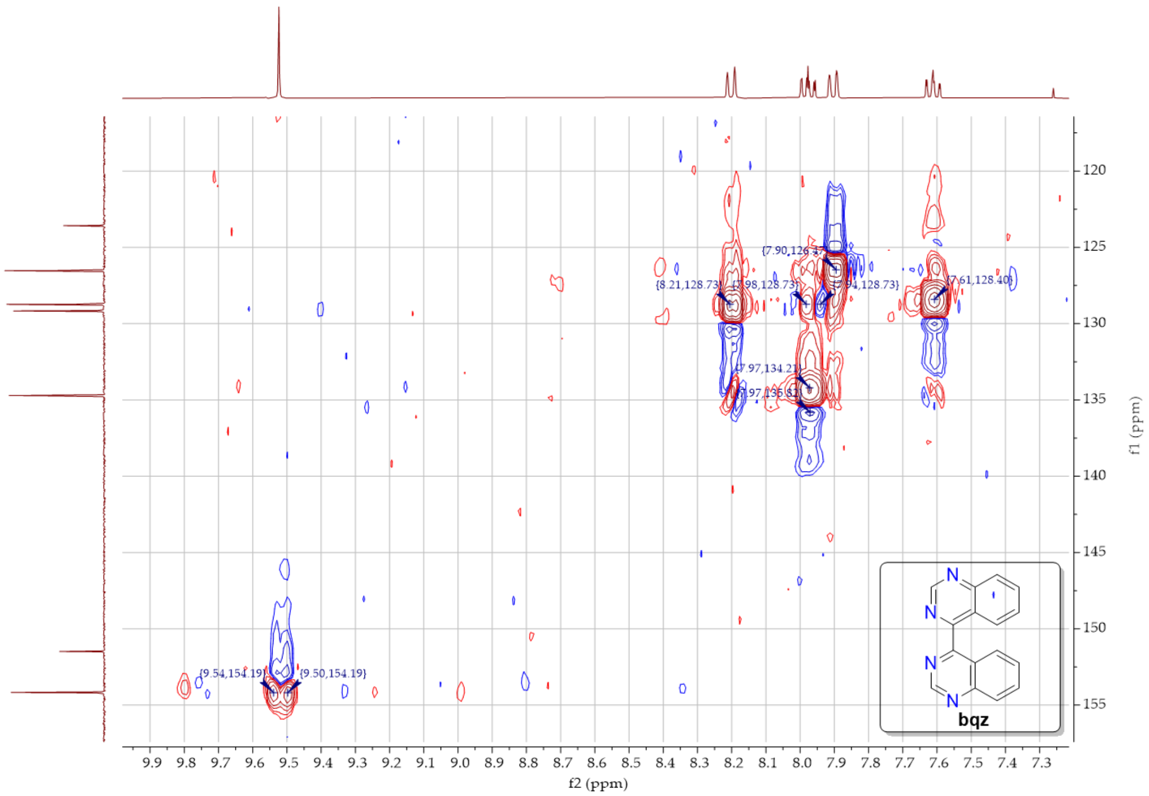

Figure S7: C,H-HSQC NMR spectrum (400/101 MHz, RT, $CDCl_3$) of 4,4′-biquinazoline (**bqz**).

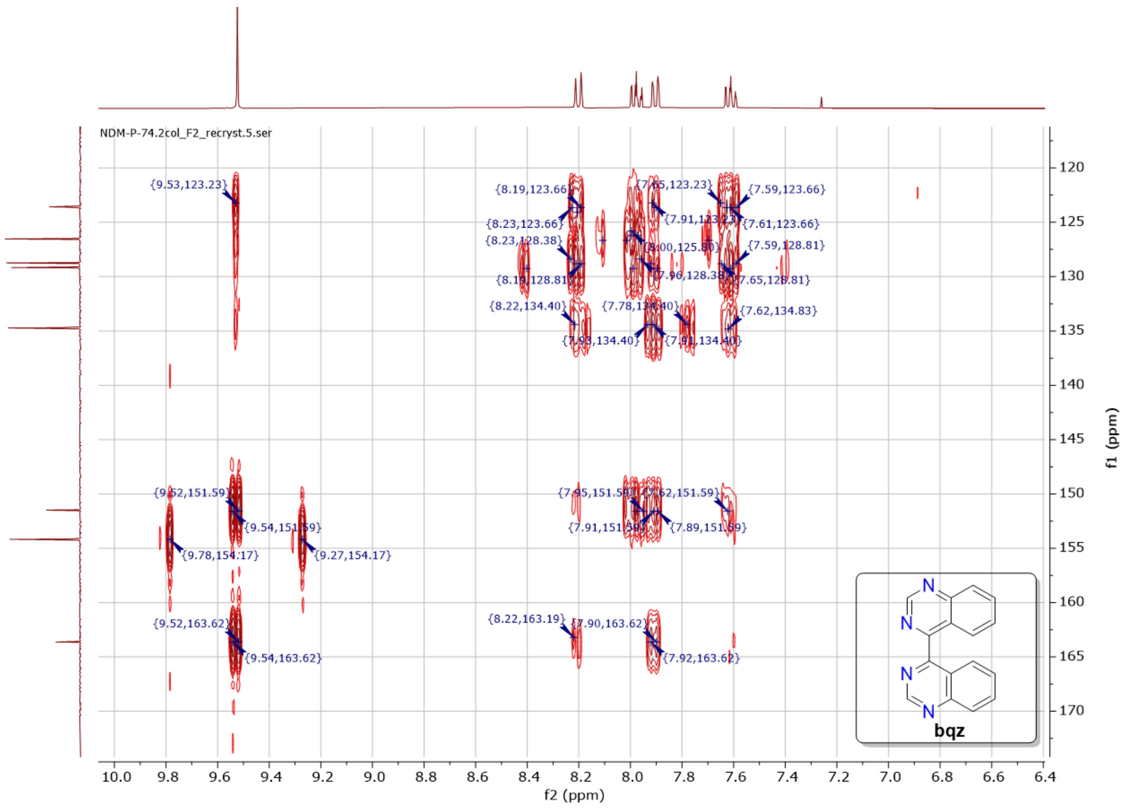

Figure S8: C,H-HMBC NMR spectrum (400/101 MHz, RT, $CDCl_3$) of 4,4′-biquinazoline (**bqz**).

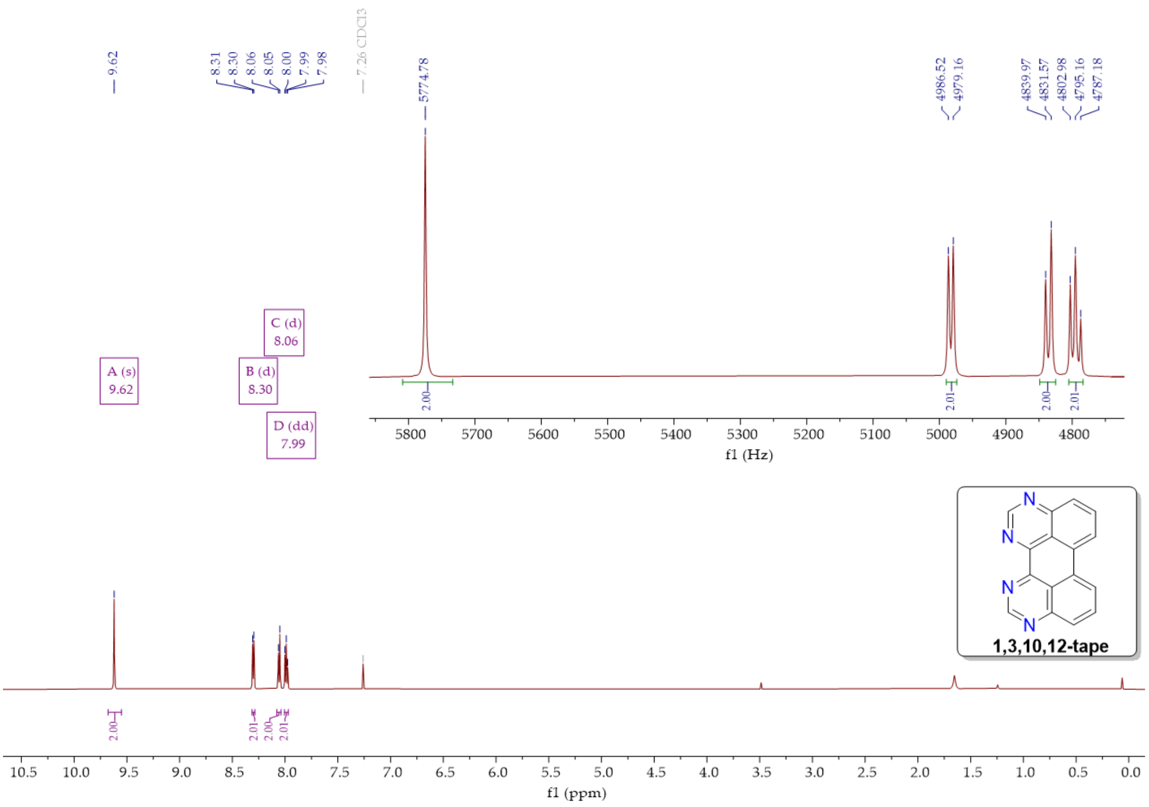

Figure S9: ^{1}H-NMR spectrum (600 MHz, RT, $CDCl_3$) of **1,3,10,12-tape**.

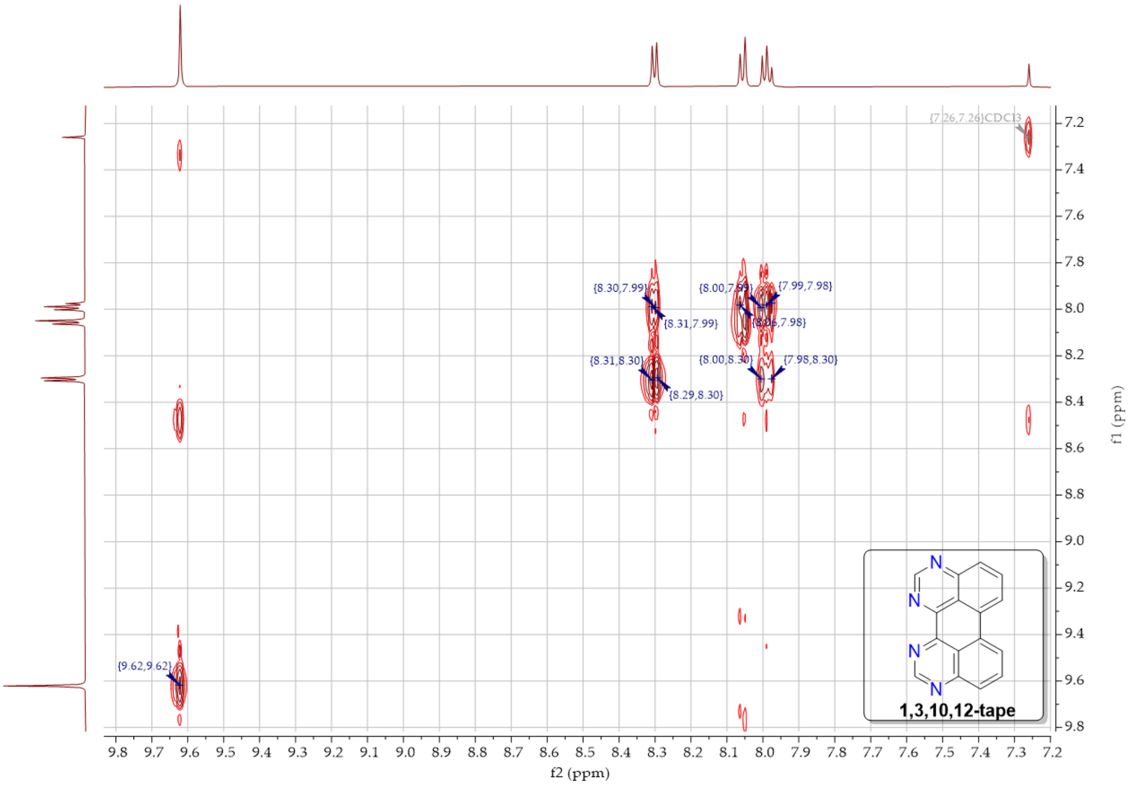

Figure S10: Selective H,H-COSY45 NMR spectrum (600 MHz, RT, $CDCl_3$) of **1,3,10,12-tape**.

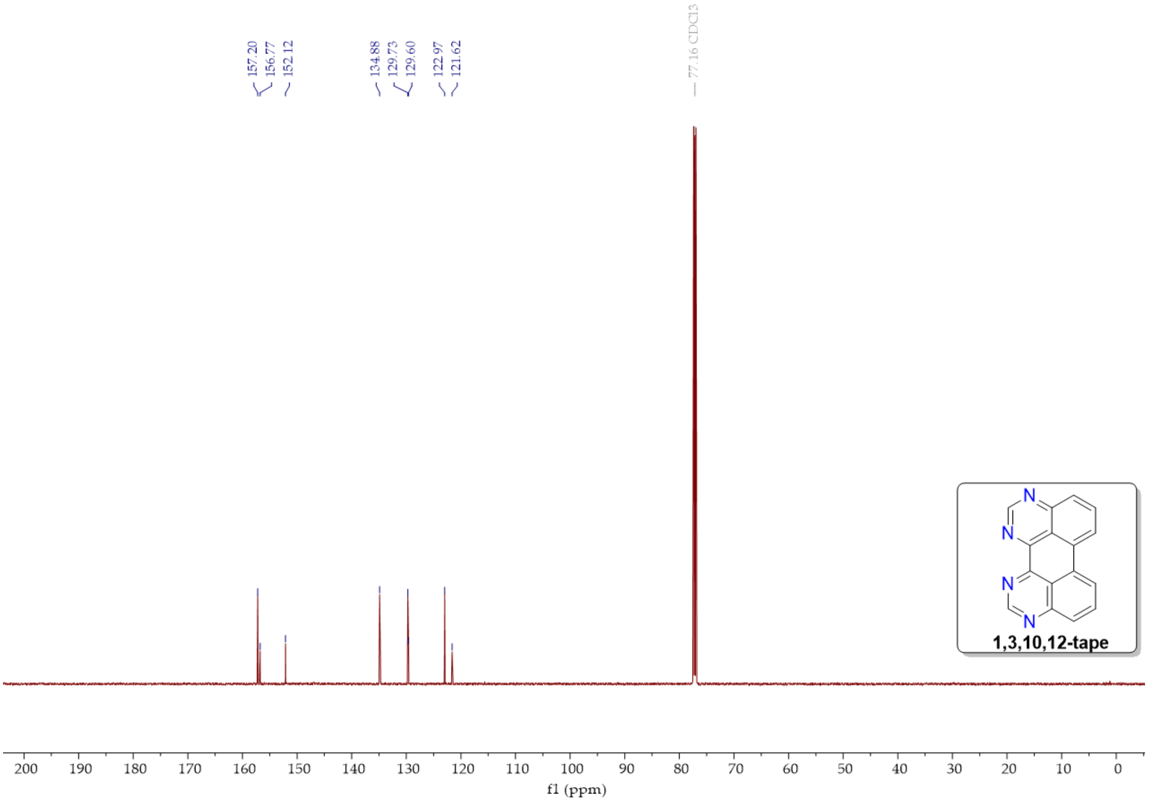

Figure S11: ^{13}C-NMR spectrum (151 MHz, RT, $CDCl_3$) of **1,3,10,12-tape**.

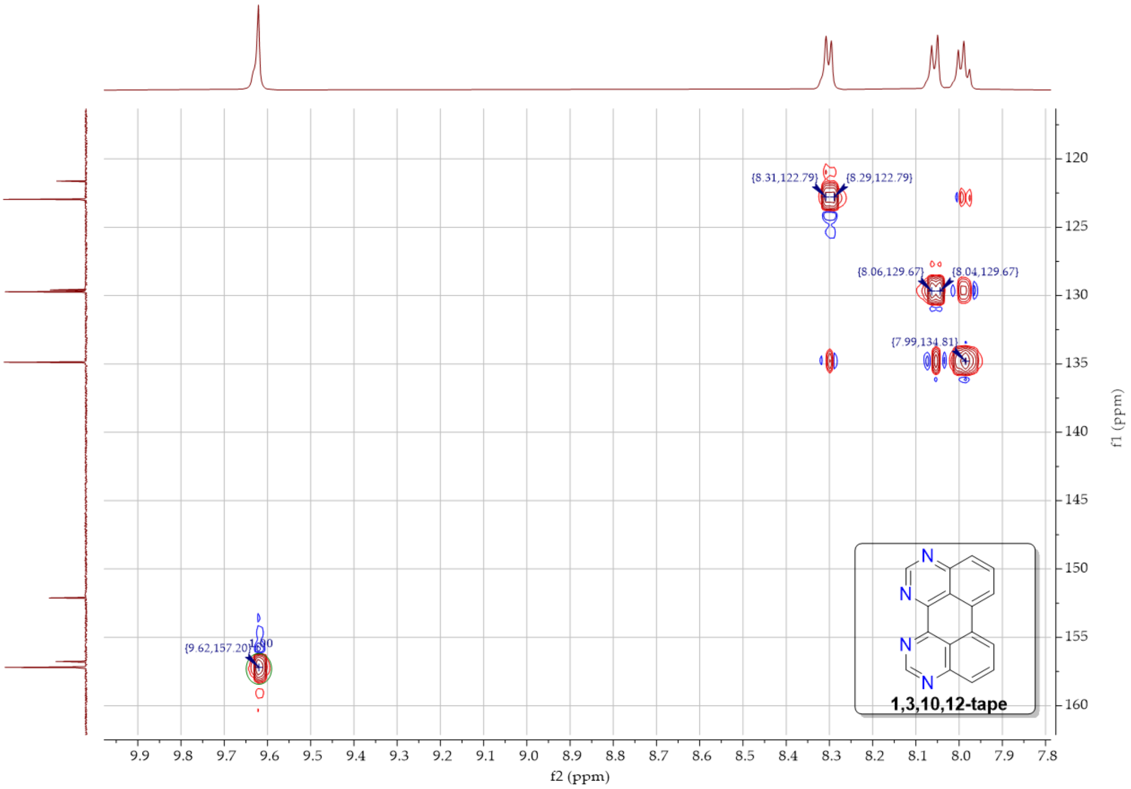

Figure S12: Selective C,H-HSQC NMR spectrum (600/151 MHz, RT, $CDCl_3$) of **1,3,10,12-tape**.

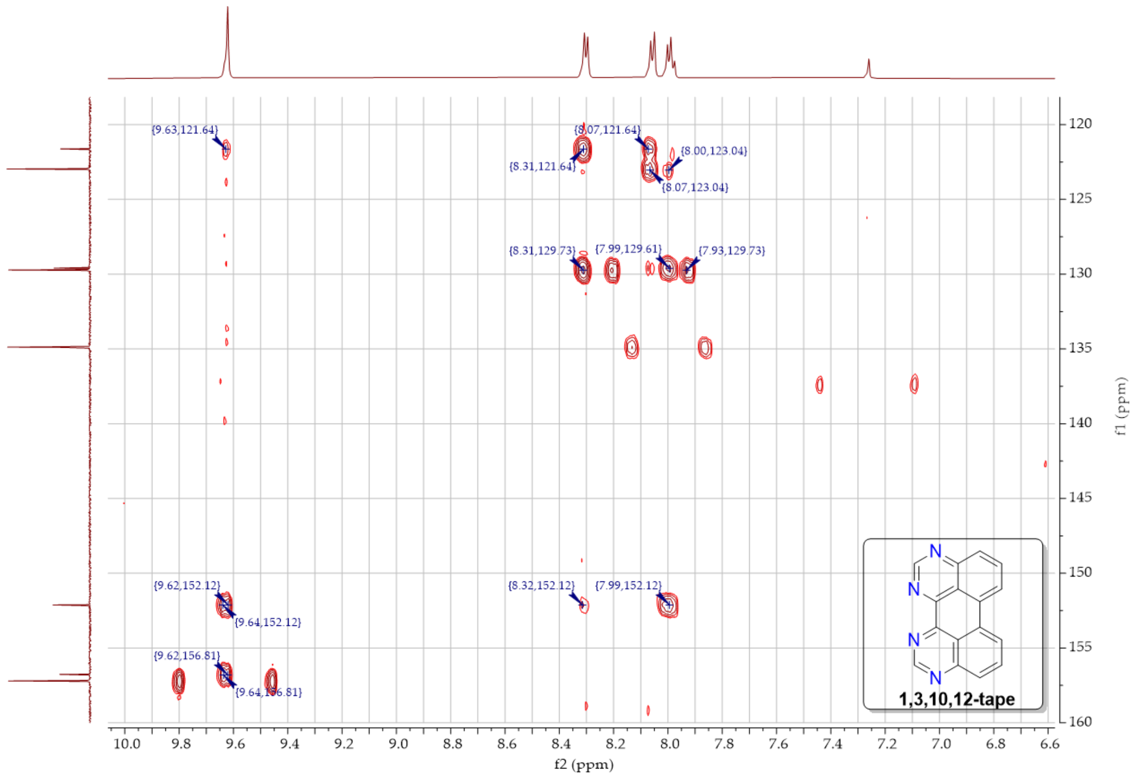

Figure S13: Selective C,H-HMBC NMR spectrum (600/151 MHz, RT, $CDCl_3$) of **1,3,10,12-tape**.

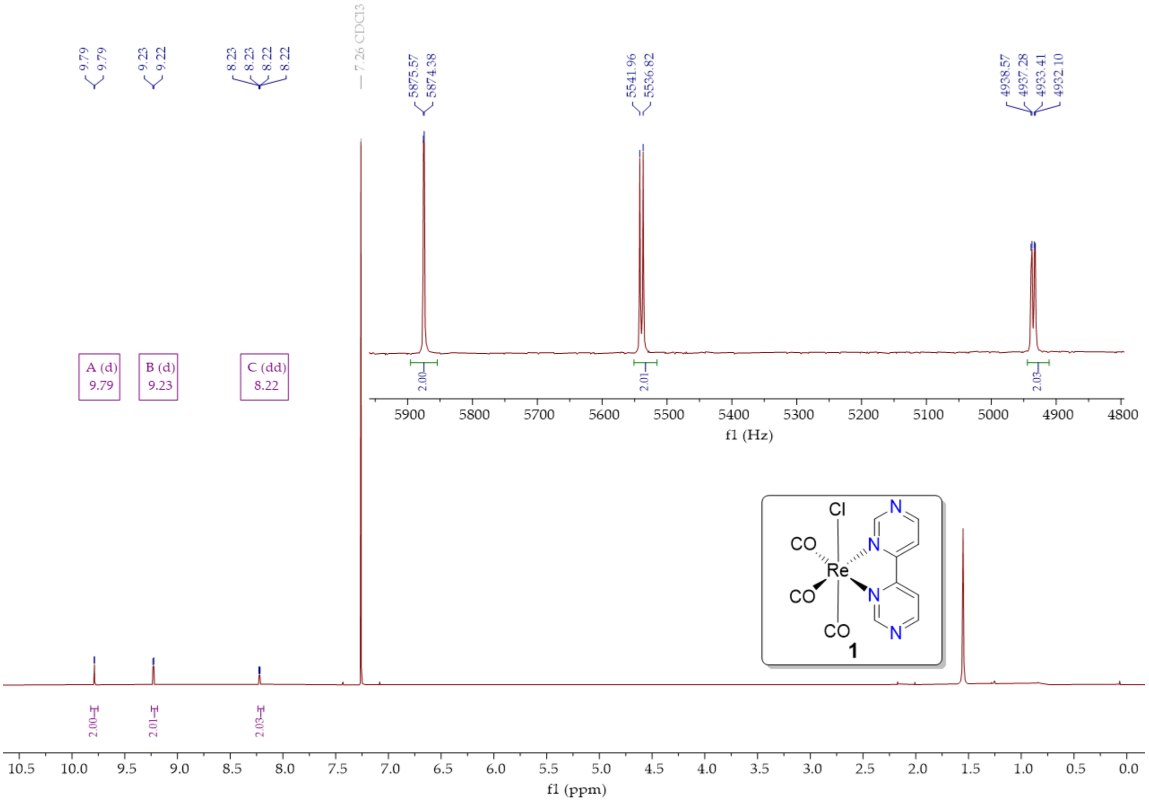

Figure S14: ^{1}H-NMR spectrum (600 MHz, RT, $CDCl_3$) of $[Re(CO)_3(bpm)Cl]$ (**1**).

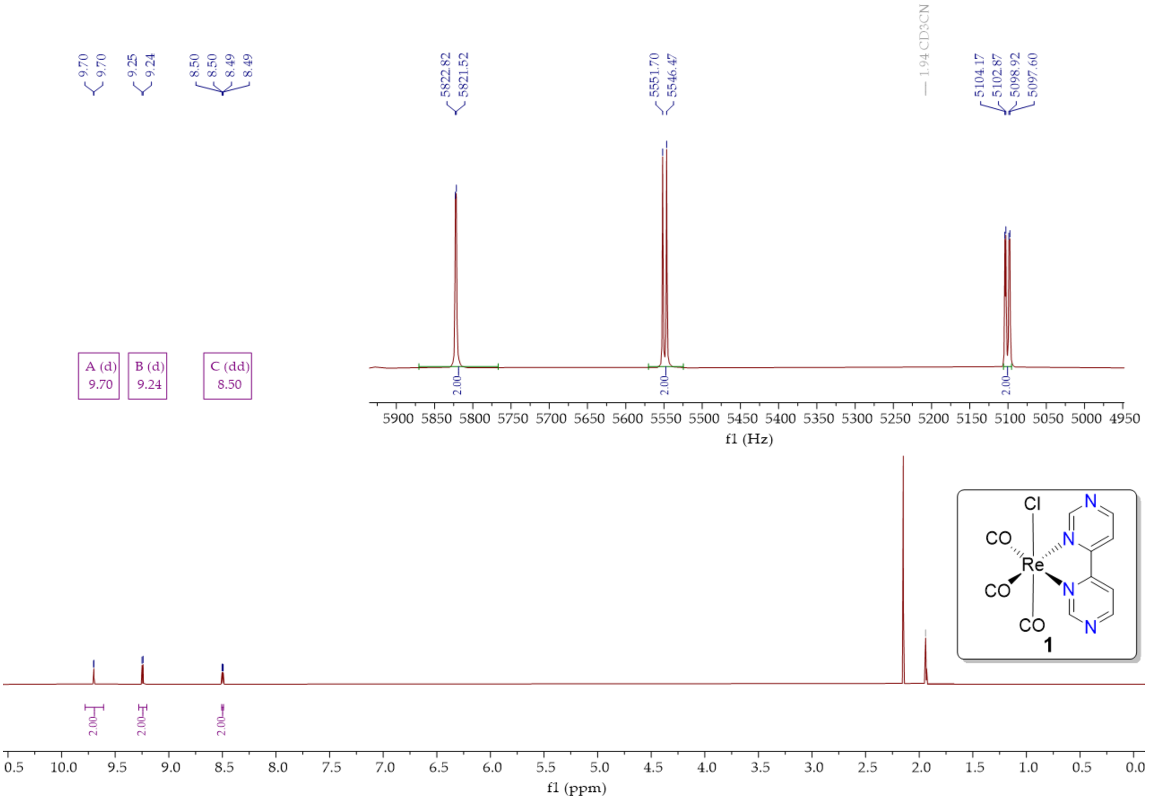

Figure S15: ^{1}H-NMR spectrum (600 MHz, RT, CD_3CN) of $[Re(CO)_3(bpm)Cl]$ (**1**).

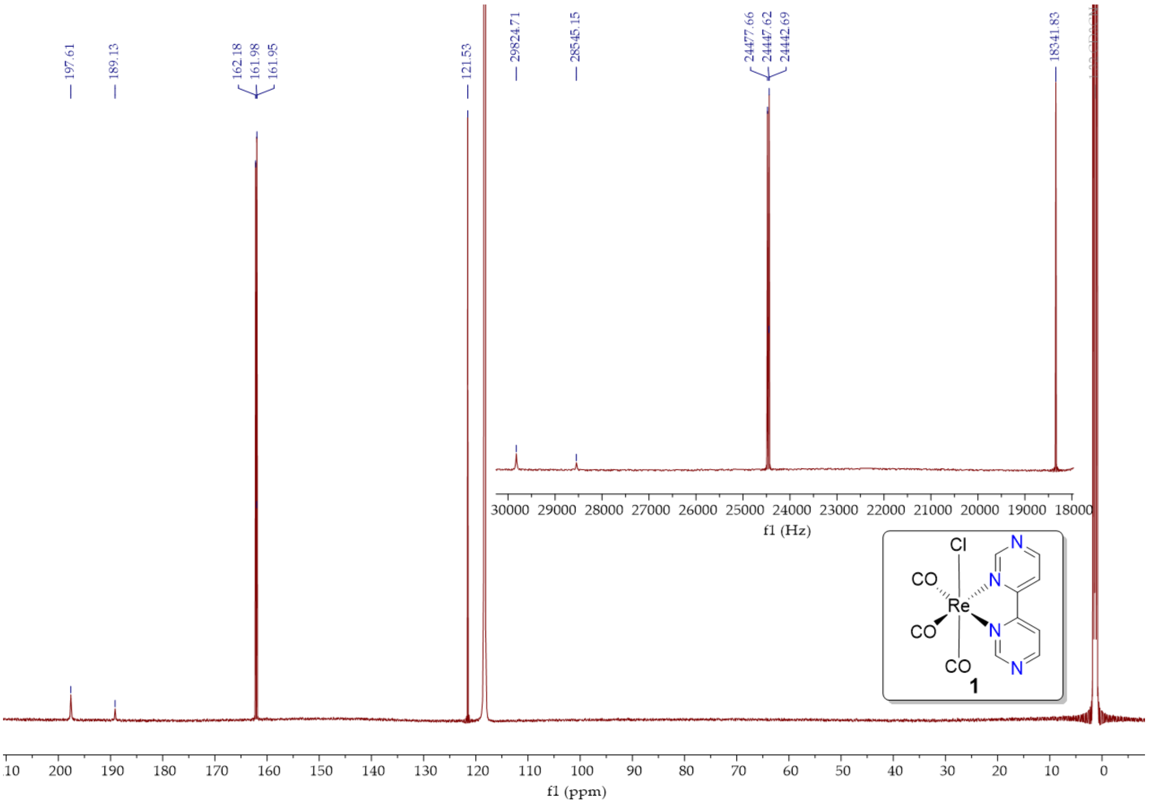

Figure S16: ^{13}C-UDEFT-NMR spectrum (151 MHz, RT, CD_3CN) of $[Re(CO)_3(bpm)Cl]$ (**1**).

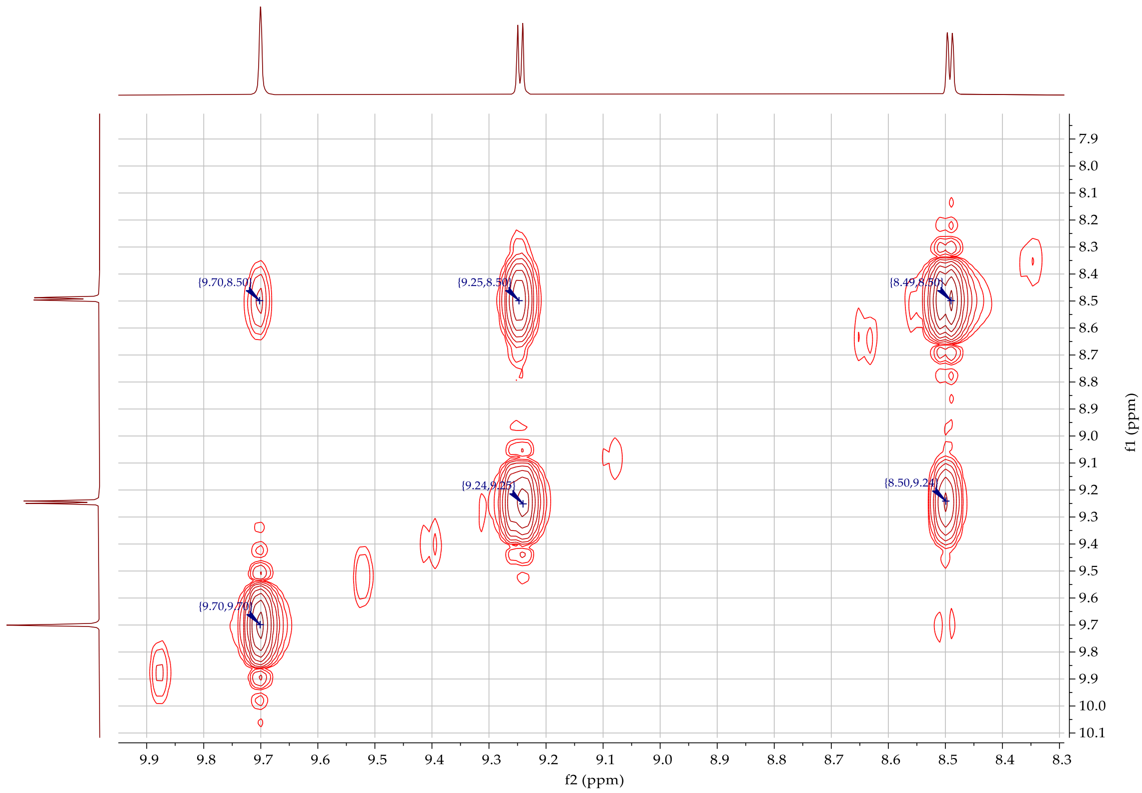

Figure S17: H,H-COSY45 NMR spectrum (400 MHz, RT, CD_3CN) of $[Re(CO)_3(bpm)Cl]$ (**1**).

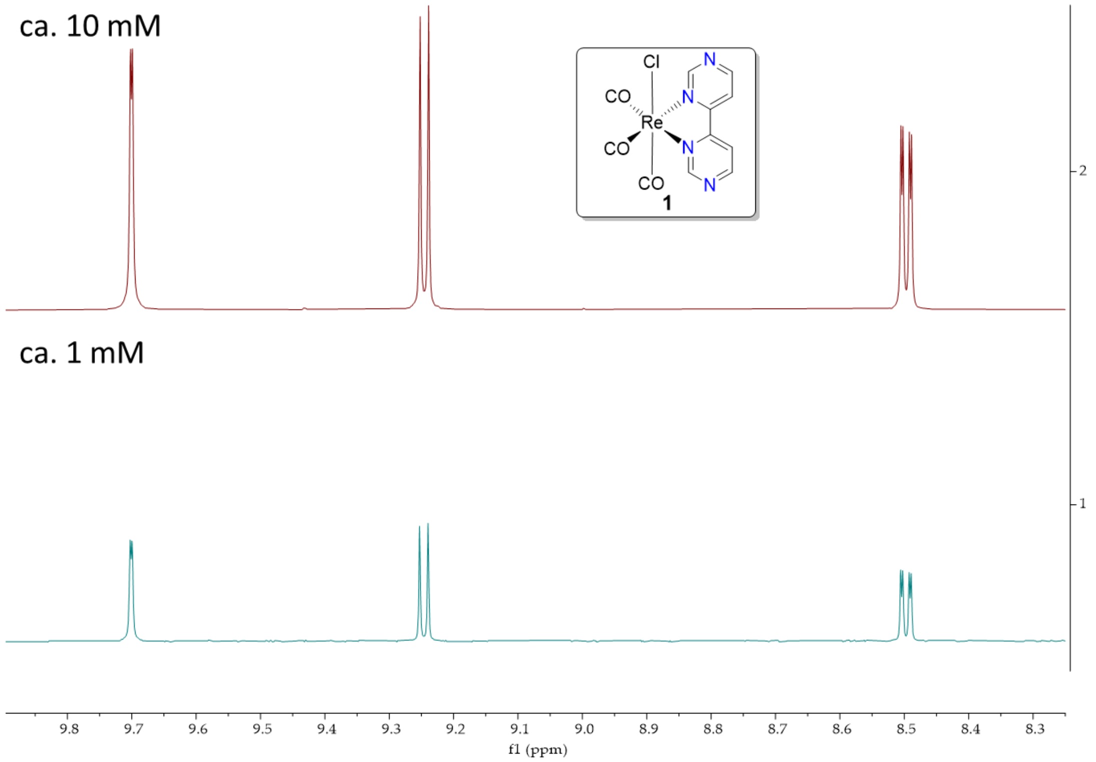

Figure S18: ^{1}H-NMR spectra (400 MHz, RT, CD_3CN) of $[Re(CO)_3(bpm)Cl]$ (**1**) (1-10 mM).

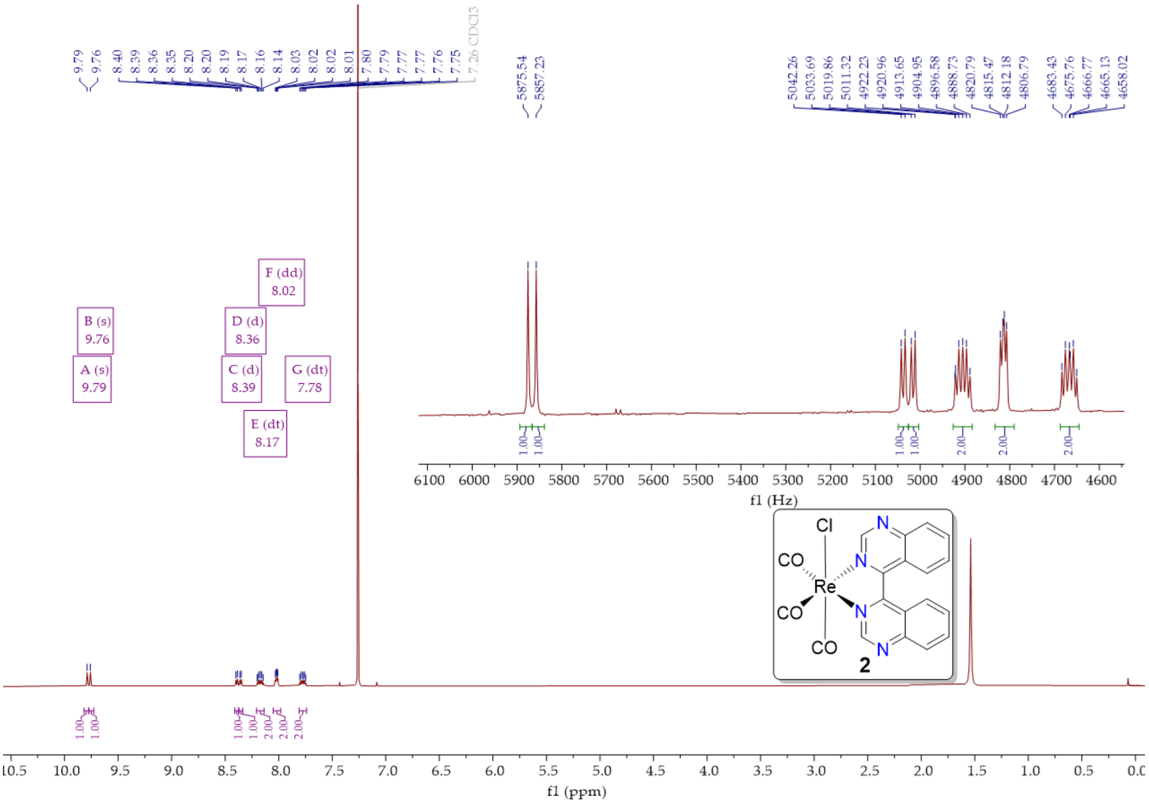

Figure S19: ^{1}H-NMR spectrum (600 MHz, RT, $CDCl_3$) of $[Re(CO)_3(bqz)Cl]$ (**2**).

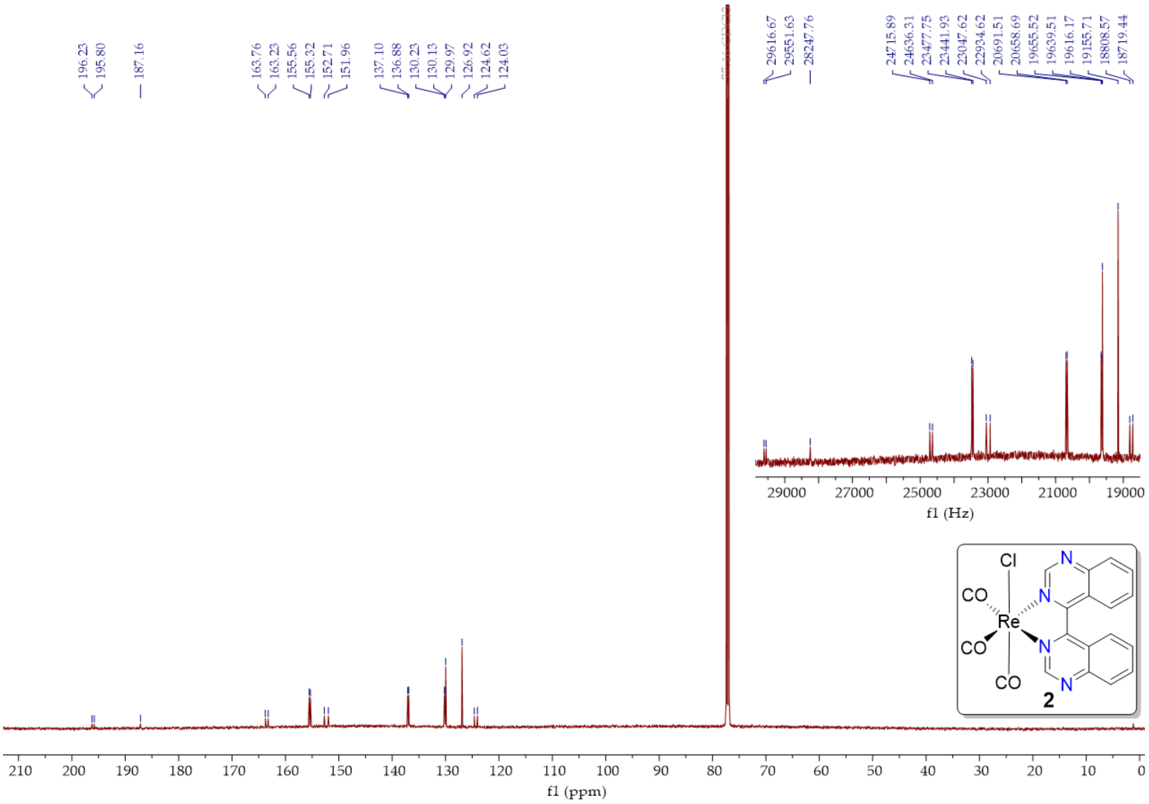

Figure S20: ^{13}C-UDEFT-NMR spectrum (151 MHz, RT, $CDCl_3$) of $[Re(CO)_3(bqz)Cl]$ (**2**).

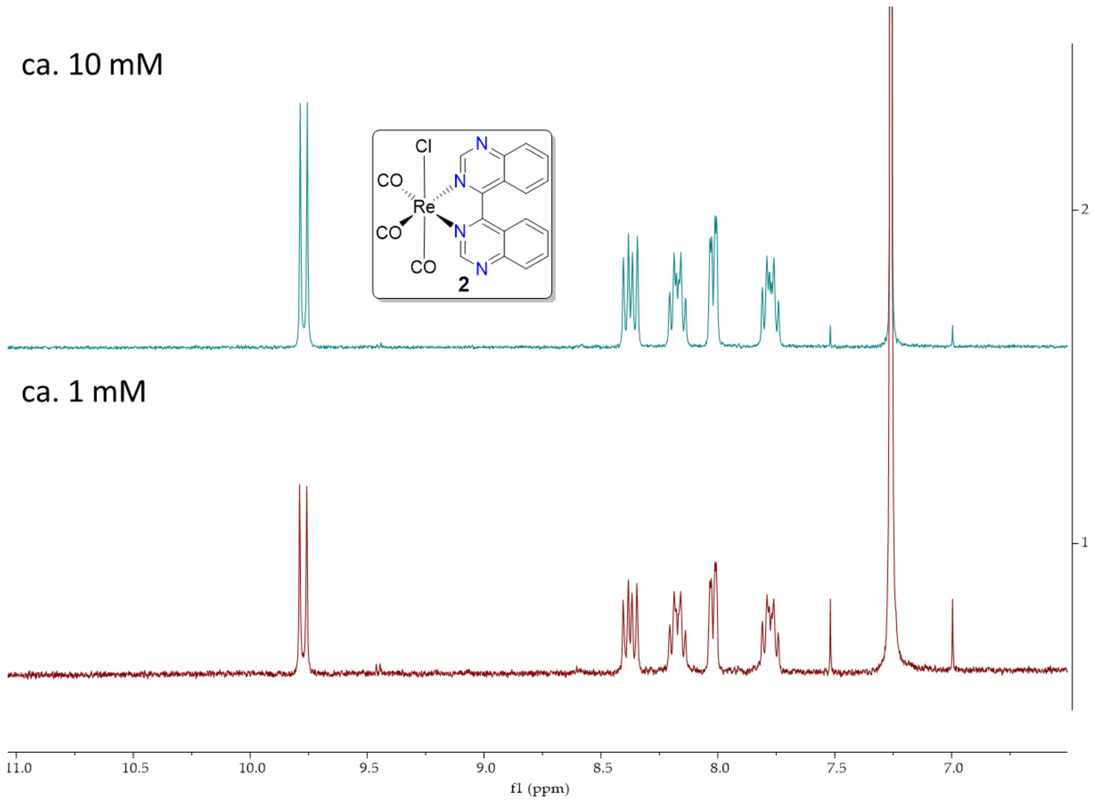

Figure S21: ^{1}H-NMR spectra (400 MHz, RT, $CDCl_3$) of $[Re(CO)_3(bqz)Cl]$ (**2**) (1-10 mM).

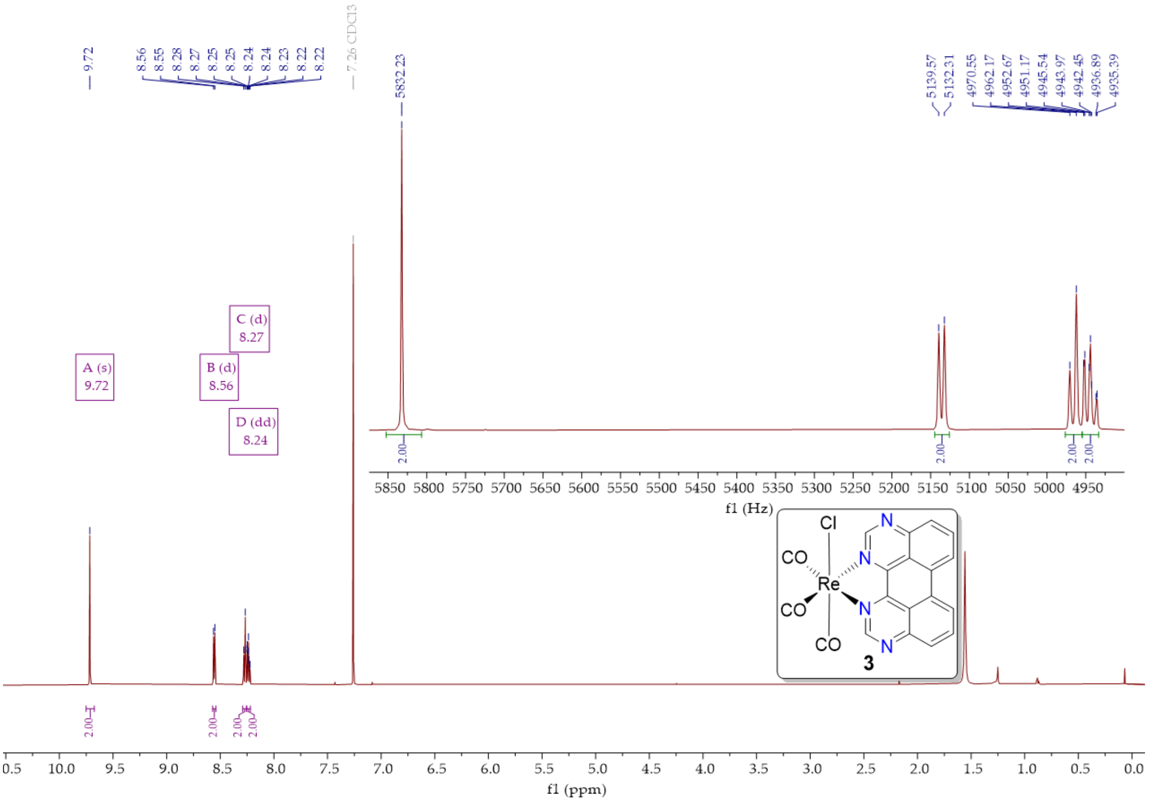

Figure S22: ^{1}H-NMR spectrum (600 MHz, RT, $CDCl_3$) of $[Re(CO)_3(1,3,10,12\text{-tape})Cl]$ (**3**).

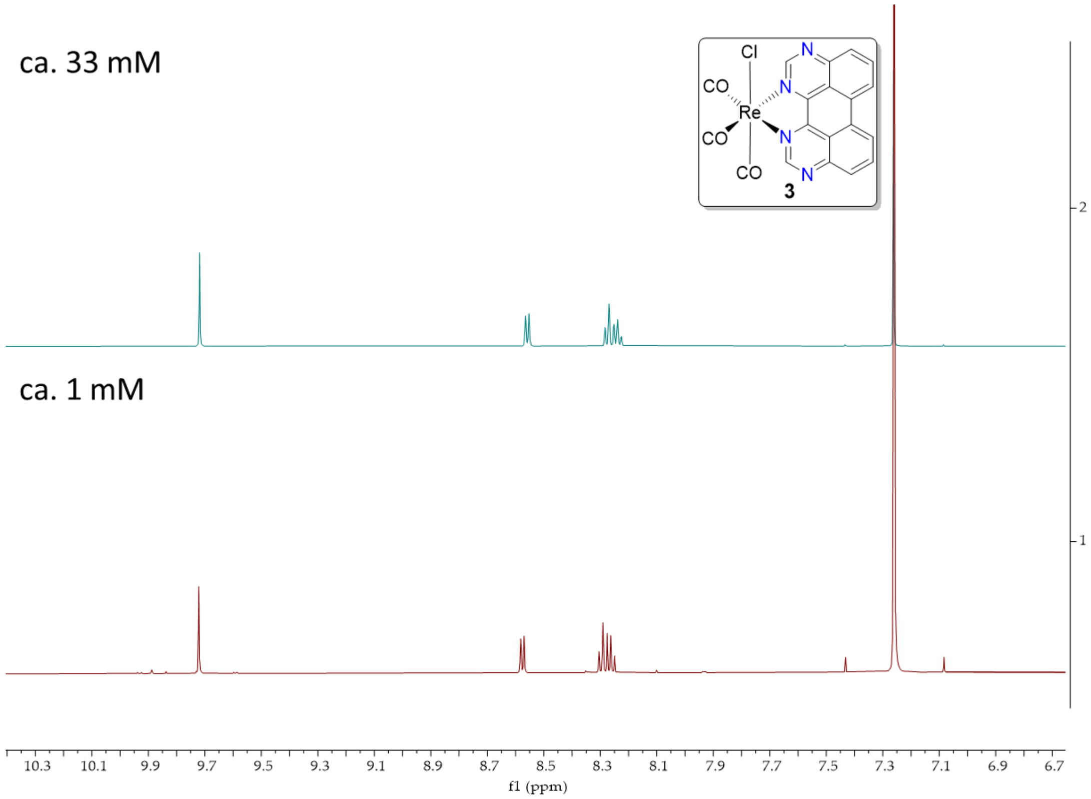

Figure S23: ^{1}H-NMR spectra (600 MHz, RT, $CDCl_3$) of [Re(CO)$_3$(1,3,10,12-tape)Cl] (**3**) (1-33 mM).

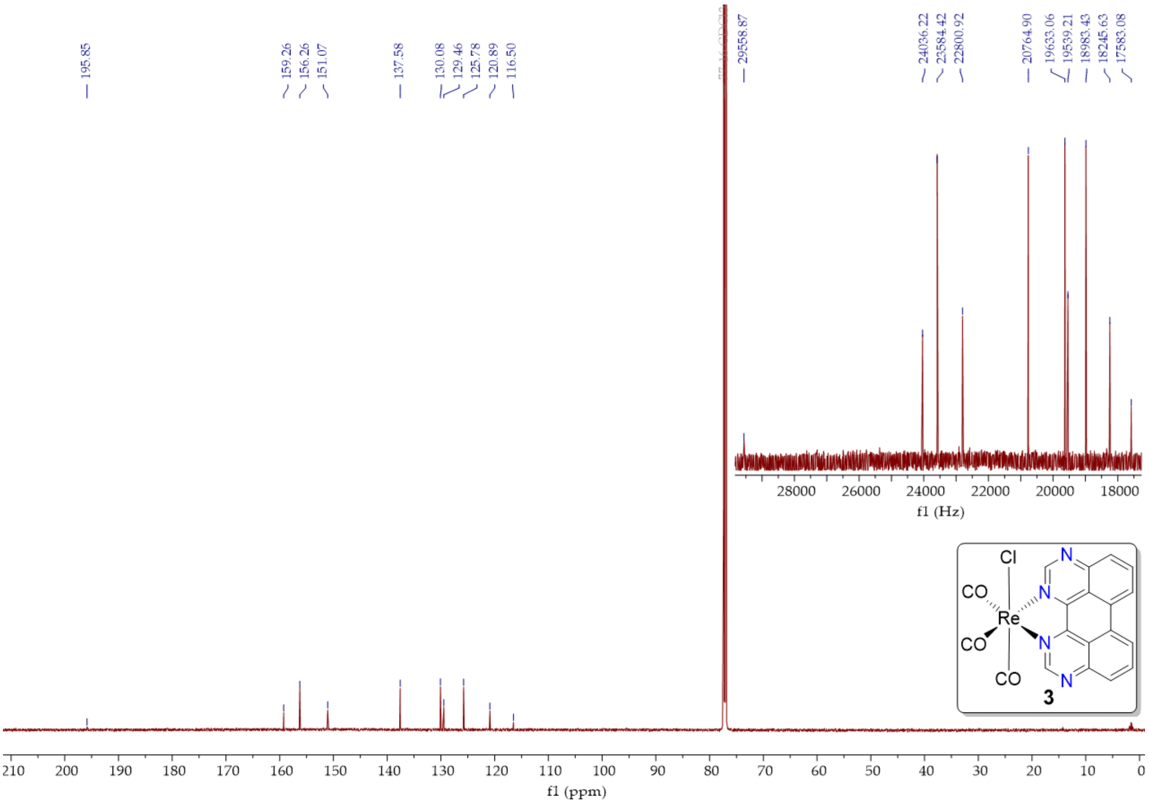

Figure S24: ^{13}C-NMR spectrum (151 MHz, RT, $CDCl_3$) of [Re(CO)$_3$(1,3,10,12-tape)Cl] (**3**).

2. High resolution mass spectra

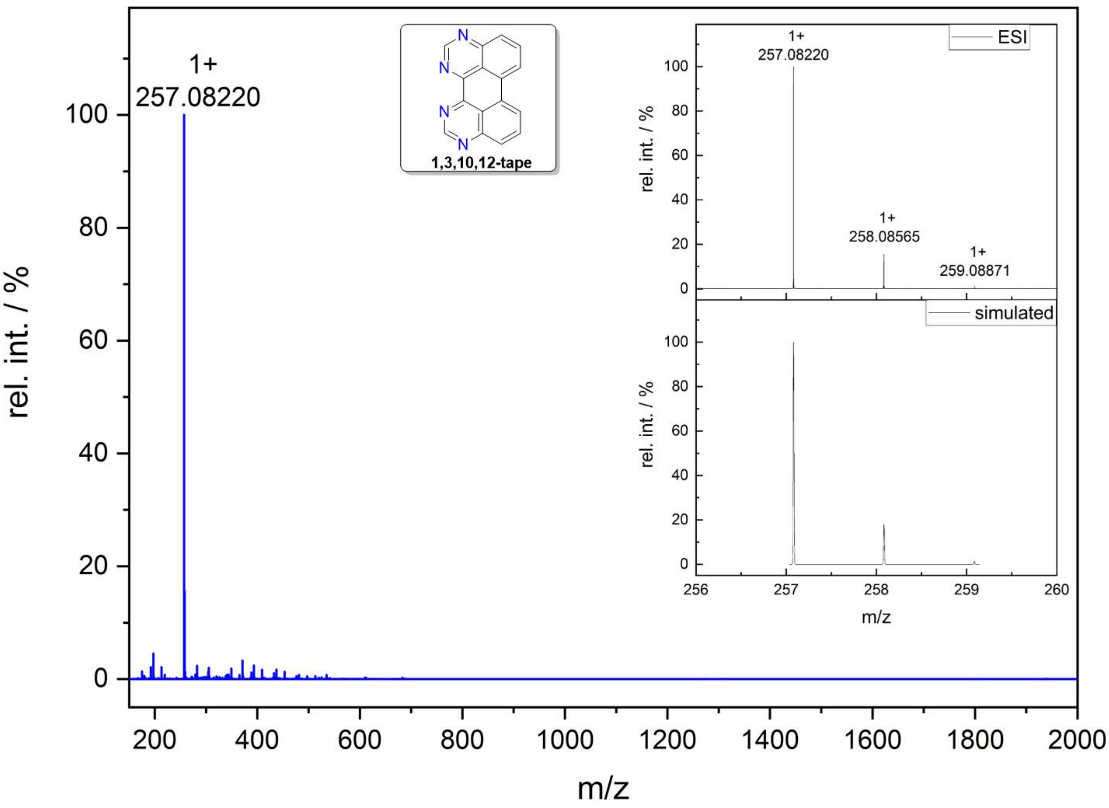

Figure S25: High-resolution ESI mass spectrum of ligand **1,3,10,12-tape** (measured from chloroform solution; M = [$C_{16}H_8N_4$]; *calc. for* [M + H^+]$^+$ $^m/_z$ = 257.08217), the inset showing the zoomed experimental as well as the simulated isotopic pattern.

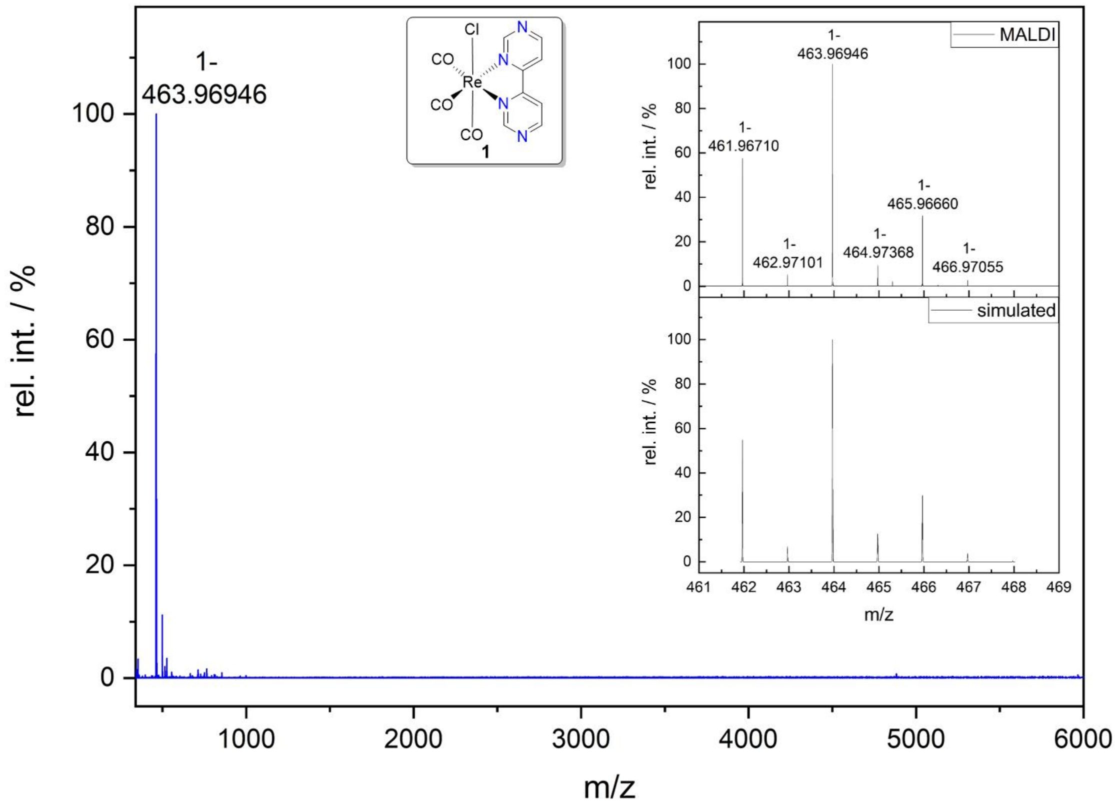

Figure S26: High-resolution MALDI mass spectrum of complex **1** (neg. mode, DCTB matrix; M = [$C_{11}H_6ClN_4O_3Re$]; *calc. for* [M + e⁻]⁻ $^{m}/_{z}$ = 463.96859), the inset showing the zoomed experimental as well as the simulated isotopic pattern.

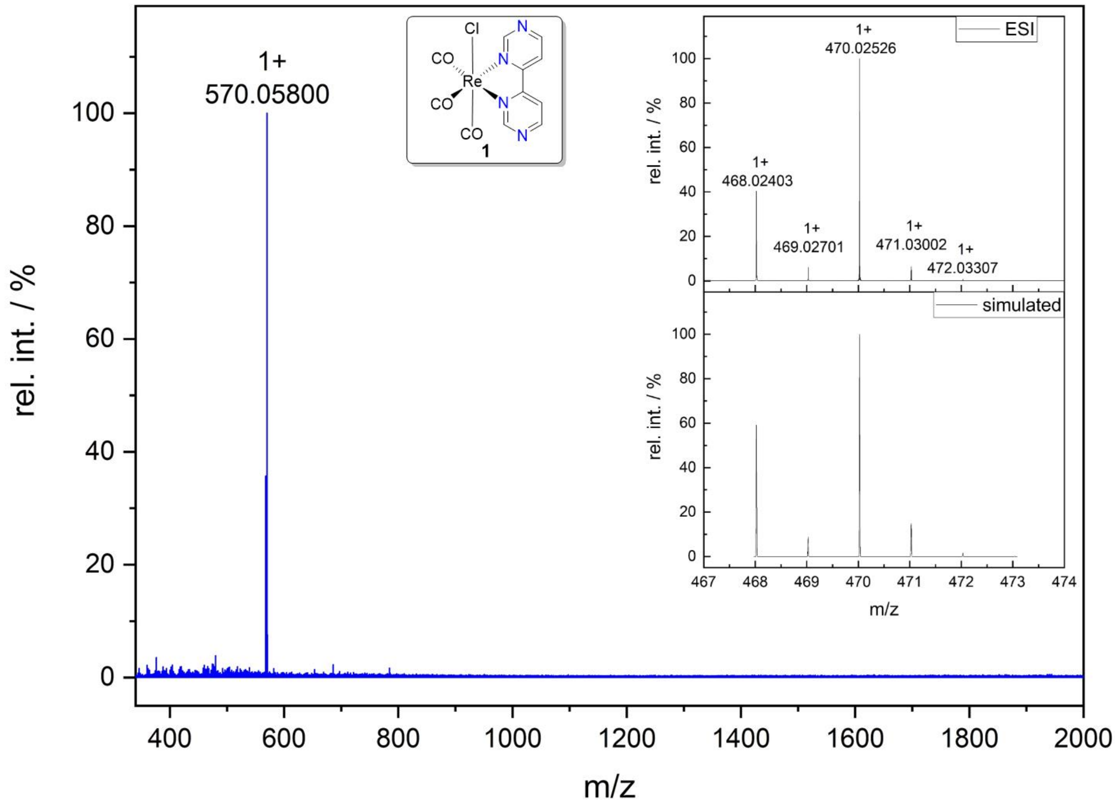

Figure S27: High-resolution ESI mass spectrum of complex **1** (measured from an acetonitrile solution; M = [$C_{11}H_6ClN_4O_3Re$]; *calc. for* [M - Cl^- + MeCN]$^+$ $^m/_z$ = 470.02629), the inset showing the zoomed experimental as well as the simulated isotopic pattern.

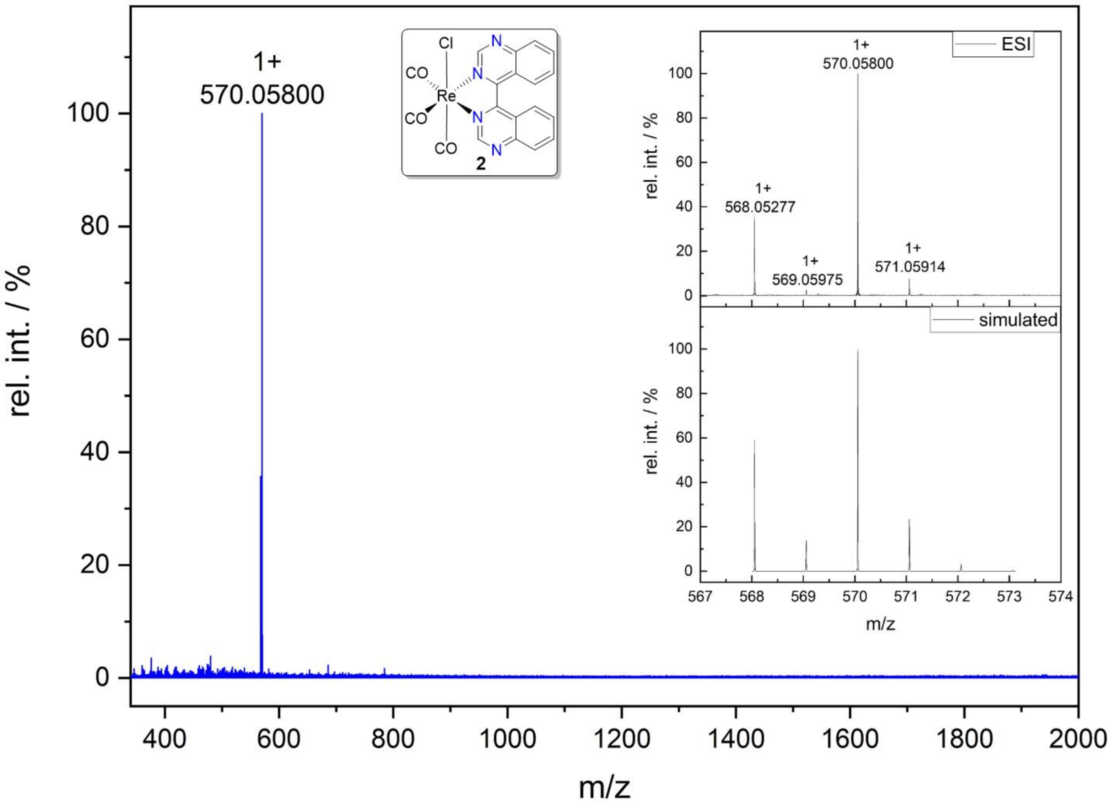

Figure S28: High-resolution ESI mass spectrum of complex **2** (measured from an acetonitrile solution; M = [$C_{19}H_{10}ClN_4O_3Re$]; *calc. for* [M - Cl^- + MeCN]$^+$ $^m/_z$ = 570.05759), the inset showing the zoomed experimental as well as the simulated isotopic pattern.

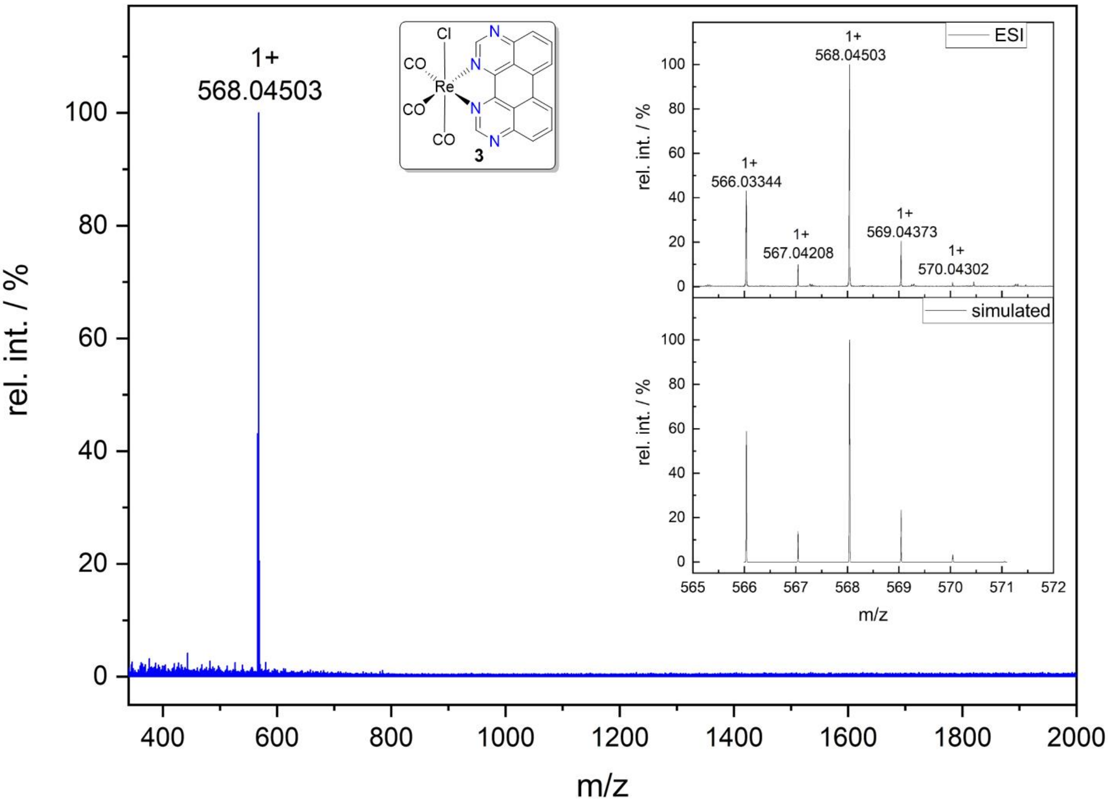

Figure S29: High-resolution ESI mass spectrum of complex **3** (measured from an acetonitrile solution; M = [$C_{19}H_8ClN_4O_3Re$]; *calc. for* [M - Cl^- + MeCN]$^+$ $^m/_z$ = 568.04194), the inset showing the zoomed experimental as well as the simulated isotopic pattern.

3. ATR-IR transmission spectra

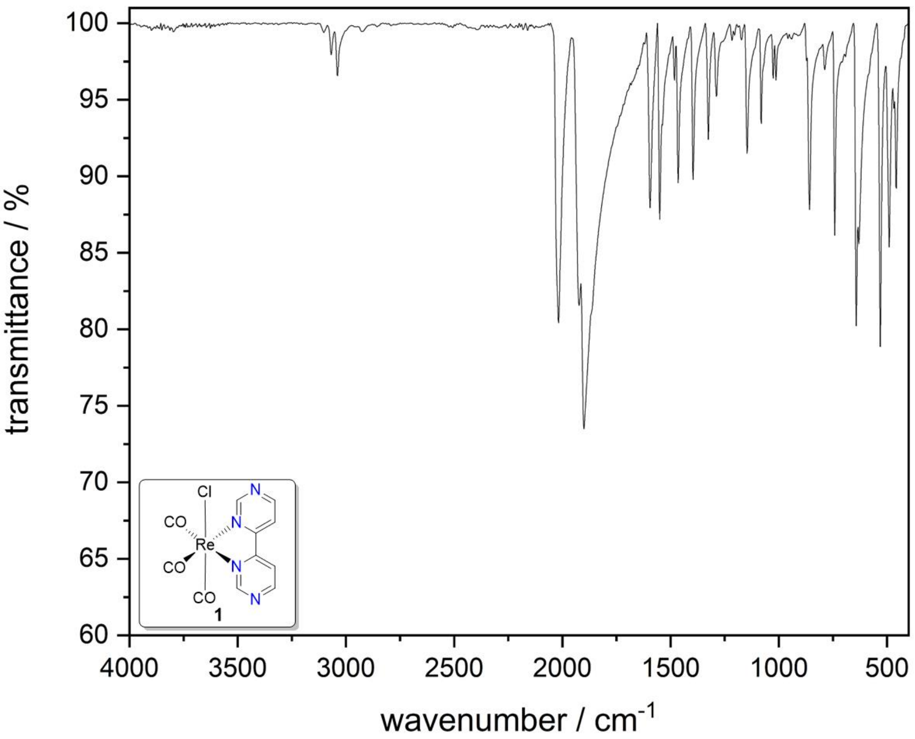

Figure S30: Solid-state ATR-IR transmission spectrum recorded for complex **1** in a range of 4000-400 cm^{-1} with a resolution of 4 cm^{-1}.

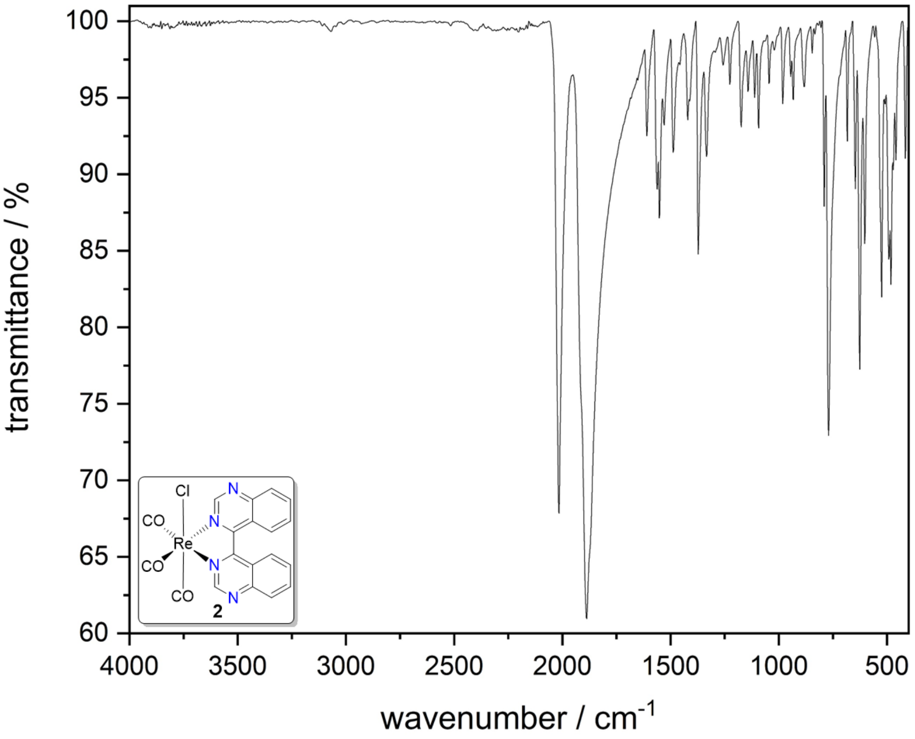

Figure S31: Solid-state ATR-IR transmission spectrum recorded for complex **2** in a range of 4000-400 cm^{-1} with a resolution of 4 cm^{-1}.

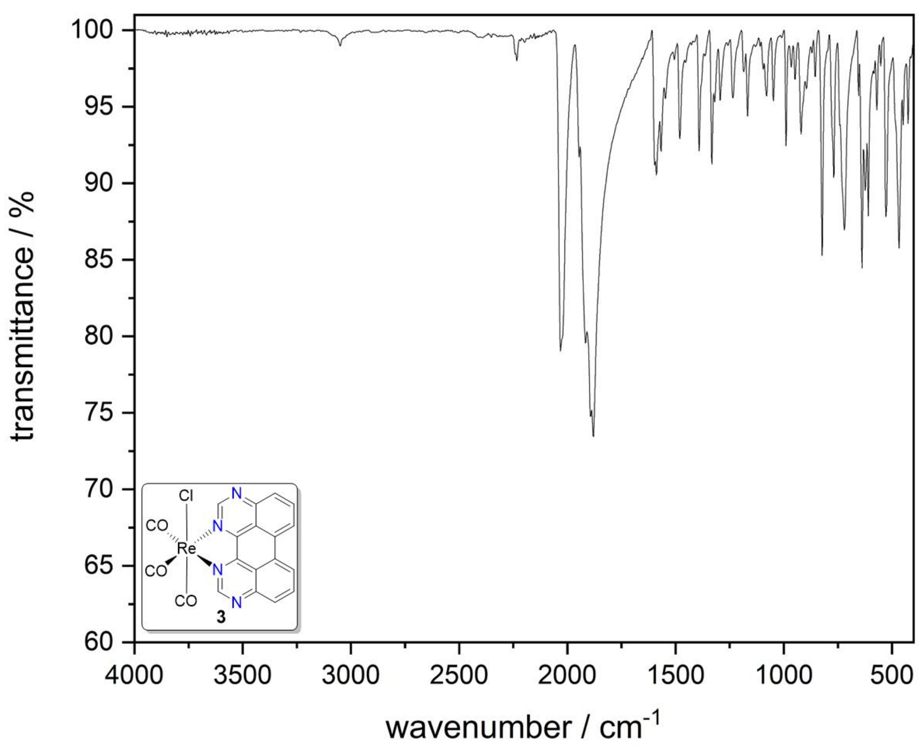

Figure S32: Solid-state ATR-IR transmission spectrum recorded for complex **3** in a range of 4000-400 cm^{-1} with a resolution of 4 cm^{-1}.

4. Crystallographic information

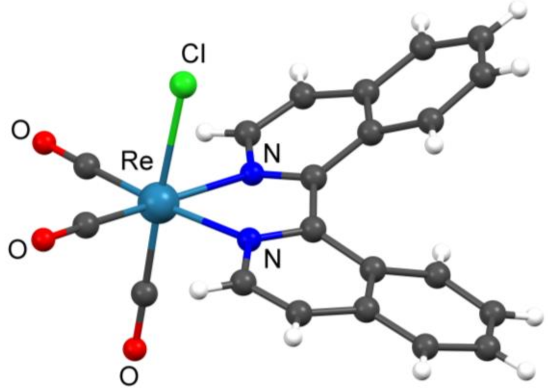

Figure S33:: Molecular structure of the chloride tris(carbonyl) biquinoline rhenium(I) complex reported by Holdt *et al.*, reproduced from CSD entry 631826; ball-and-stick depiction created with Mercury 2020.2.0 (Build 290188).

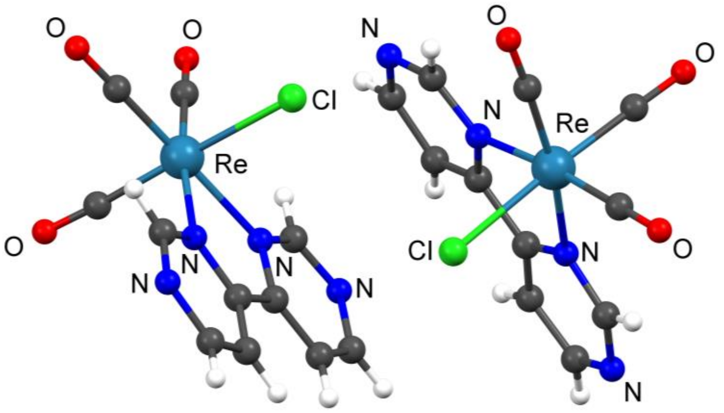

Figure S34: Molecular structure of **1** as determined by scXRD analysis; ball-and-stick depiction showing the asymmetric unit along the a-axis; created with Mercury 2020.2.0 (Build 290188).

Table S1: Angles characterizing the distortion of the bipyrimidine unit in Re complexes **1** and **3**.

	1	3
∢(N-C-N) [°]	126.2(5)	127.2(8)
	125.8(5)	126.0(8)
	125.1(5)	
	125.9(5)	
∢(C-N-C) [°]	116.5(5)	114.8(7)
	117.1(4)	115.2(7)
	117.3(4)	117.2(7)
	117.4(4)	119.0(7)
	115.7(6)	
	116.1(5)	
	116.9(5)	
	116.5(5)	

Table S2: Bond distances in the coordination sphere of Re complexes **1** and **3** in comparison those of the published structure of [Re(CO)$_3$(1,1′-bisisoquinoline)Cl] by Holdt (ref. [6]).

	1	CSD entry 631826	3
d(Re-Cl) [Å]	2.469(1)	2.503(2)	2.475(2)
	2.475(1)		
d(Re-CO) [Å]	1.914(6)	1.921(7)	1.923(8)
	1.915(6)	1.924(8)	1.912(9)
	1.913(5)		
	1.900(6)		
	1.908(4)	1.907(7)	1.901(9)
	1.923(6)		
d(Re-N) [Å]	2.181(4)	2.200(5)	2.180(7)
	2.176(4)	2.180(5)	2.158(6)

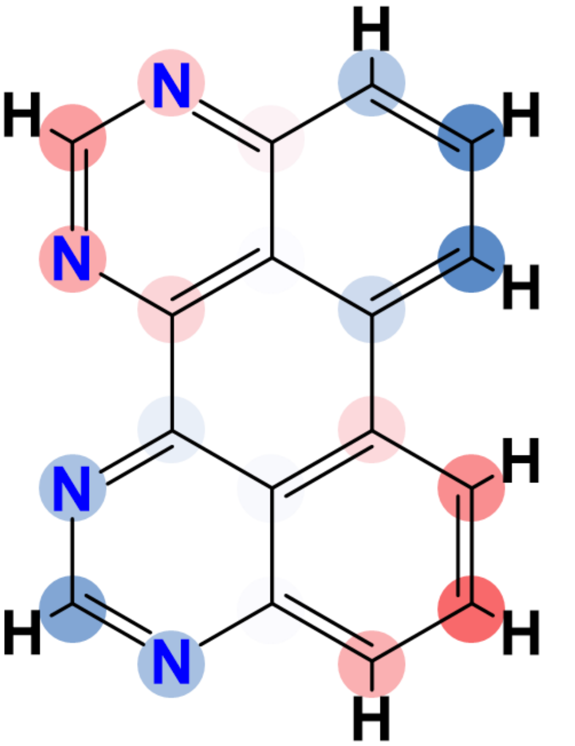

d [Å]
-0.1294
-0.1291
-0.099
-0.0705
-0.0681
-0.0619
-0.0412
-0.0202
-0.0094
-0.0069
-0.0059
0.0054
0.0337
0.0377
0.0553
0.0806
0.0872
0.1007
0.119
0.1597

Figure S35: Illustration of deviations of individual atoms of the **1,3,10,12-tape** ligand in **3** from the respective calculated mean plane; values determined with the Diamond Version 4.6.8 Software.

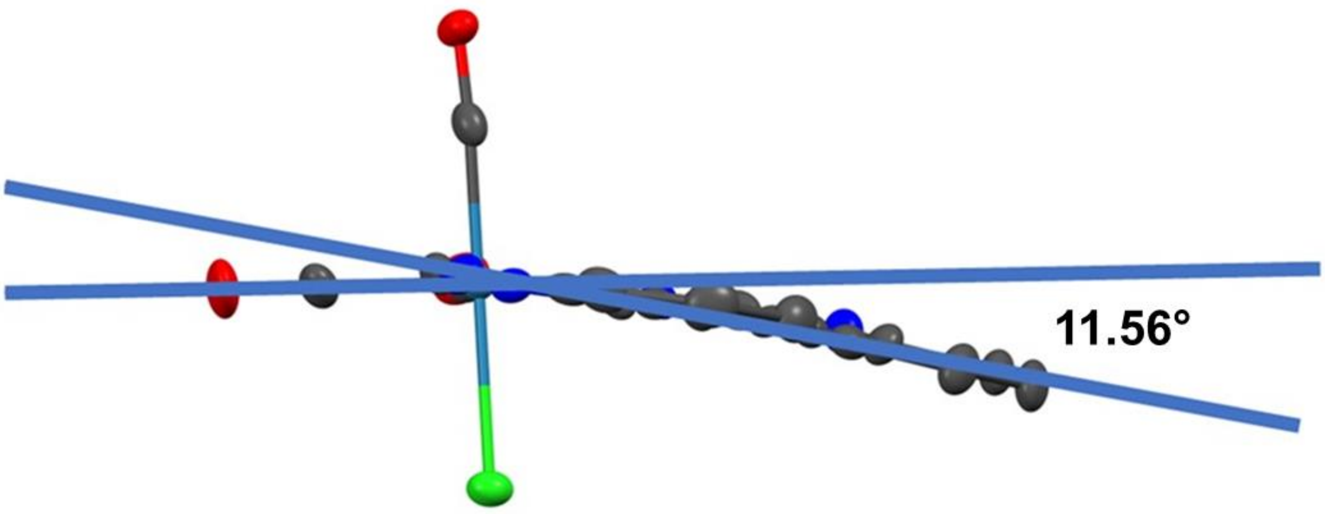

Figure S36: Ball-and-stick depiction of **3**·$CHCl_3$ as determined by scXRD, highlighting the 11.56° tilt observed in the crystal structure of **3** as defined by the plane of the **1,3,10,12-tape** ligand *vs.* the plane of the Re center and the two CO-ligands *trans* to **1,3,10,12-tape**; protons and solvent molecule omitted for clarity; created with Mercury 2020.2.0 (Build 290188).

Table S3: Crystal data and structure refinement for **1** (CCDC #2225447). Crystals were obtained by slow diffusion of *n*-hexane into a concentrated chloroform solution.

Identification code	**1**, CCDC #2225447
Empirical formula	$C_{11}H_6ClN_4O_3Re$
Formula weight	463.85
Temperature/K	150.0
Crystal system	triclinic
Space group	P-1
a/Å	7.8191(3)
b/Å	7.9719(3)
c/Å	21.5105(9)
α/°	80.661(2)
β/°	84.045(2)
γ/°	89.498(2)
Volume/Å^3	1315.86(9)
Z	4
ρ_{calc}g/cm^3	2.341
μ/mm^{-1}	9.449
F(000)	864.0
Crystal size/mm^3	0.309 × 0.224 × 0.22
Radiation	MoKα (λ = 0.71073)
2Θ range for data collection/°	3.858 to 55.002
Index ranges	$-10 \leq h \leq 10$, $-10 \leq k \leq 10$, $-27 \leq l \leq 27$
Reflections collected	27494
Independent reflections	6037 [R_{int} = 0.0740, R_{sigma} = 0.0577]
Data/restraints/parameters	6037/0/361
Goodness-of-fit on F^2	1.095
Final R indexes [I>=2σ (I)]	R_1 = 0.0343, wR_2 = 0.0878
Final R indexes [all data]	R_1 = 0.0370, wR_2 = 0.0893
Largest diff. peak/hole / e Å^{-3}	2.61/-2.28

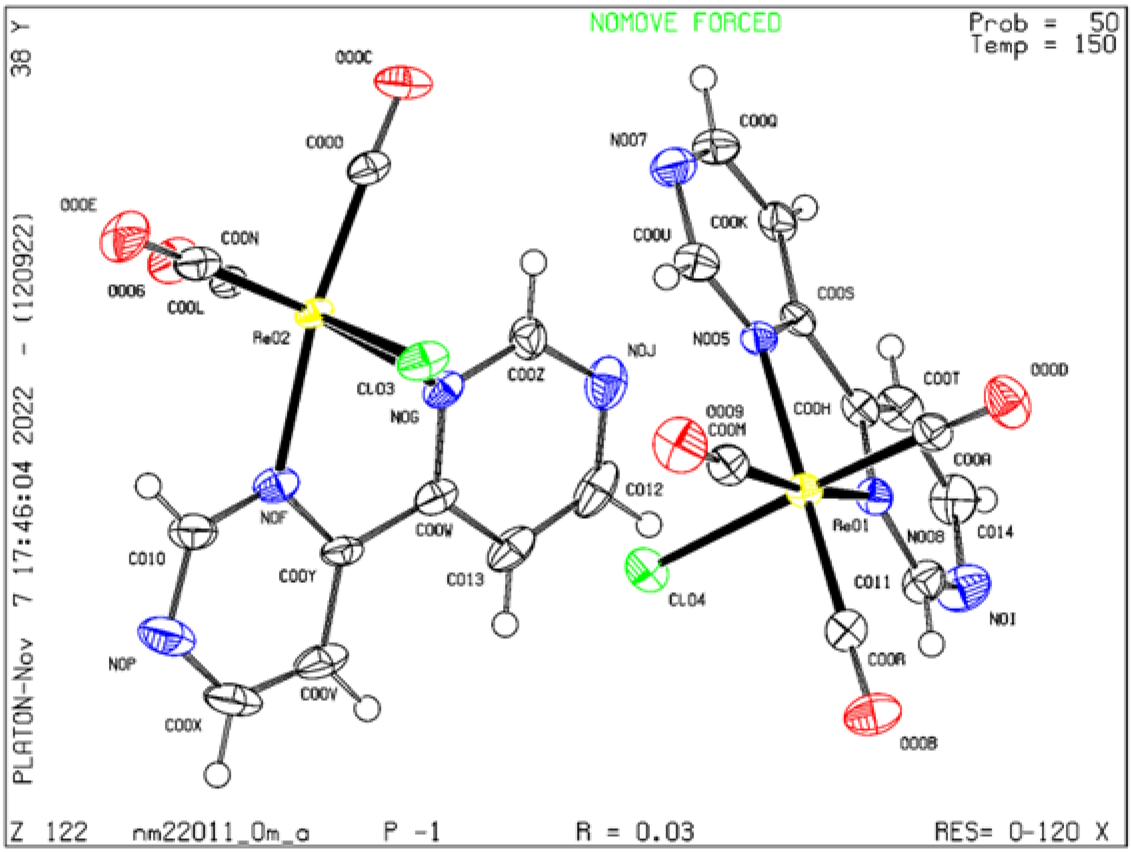

Figure S37: ORTEP depiction of **1**; the ellipsoids are drawn at the 50% probability level. H-atoms were added at fixed positions.

Table S4: Crystal data and structure refinement for **3** (CCDC #2225446). Crystals were obtained by slow diffusion of *n*-hexane into a concentrated chloroform solution.

Identification code	**3**, CCDC #2225446
Empirical formula	$C_{20}H_9Cl_4N_4O_3Re$
Formula weight	681.31
Temperature/K	150.0
Crystal system	monoclinic
Space group	$P2_1/c$
a/Å	6.2686(4)
b/Å	18.5609(13)
c/Å	18.7584(13)
α/°	90
β/°	99.606(3)
γ/°	90
Volume/Å^3	2152.0(3)
Z	4
ρ_{calc}g/cm^3	2.103
μ/mm^{-1}	6.176
F(000)	1296.0
Crystal size/mm^3	0.103 × 0.079 × 0.078
Radiation	MoKα (λ = 0.71073)
2Θ range for data collection/°	4.388 to 55.248
Index ranges	-8 ≤ h ≤ 8, -24 ≤ k ≤ 23, -24 ≤ l ≤ 24
Reflections collected	33758
Independent reflections	4971 [R_{int} = 0.1610, R_{sigma} = 0.0897]
Data/restraints/parameters	4971/0/289
Goodness-of-fit on F^2	1.033
Final R indexes [I>=2σ (I)]	R_1 = 0.0503, wR_2 = 0.1224
Final R indexes [all data]	R_1 = 0.0726, wR_2 = 0.1363
Largest diff. peak/hole / e Å^{-3}	2.01/-1.54

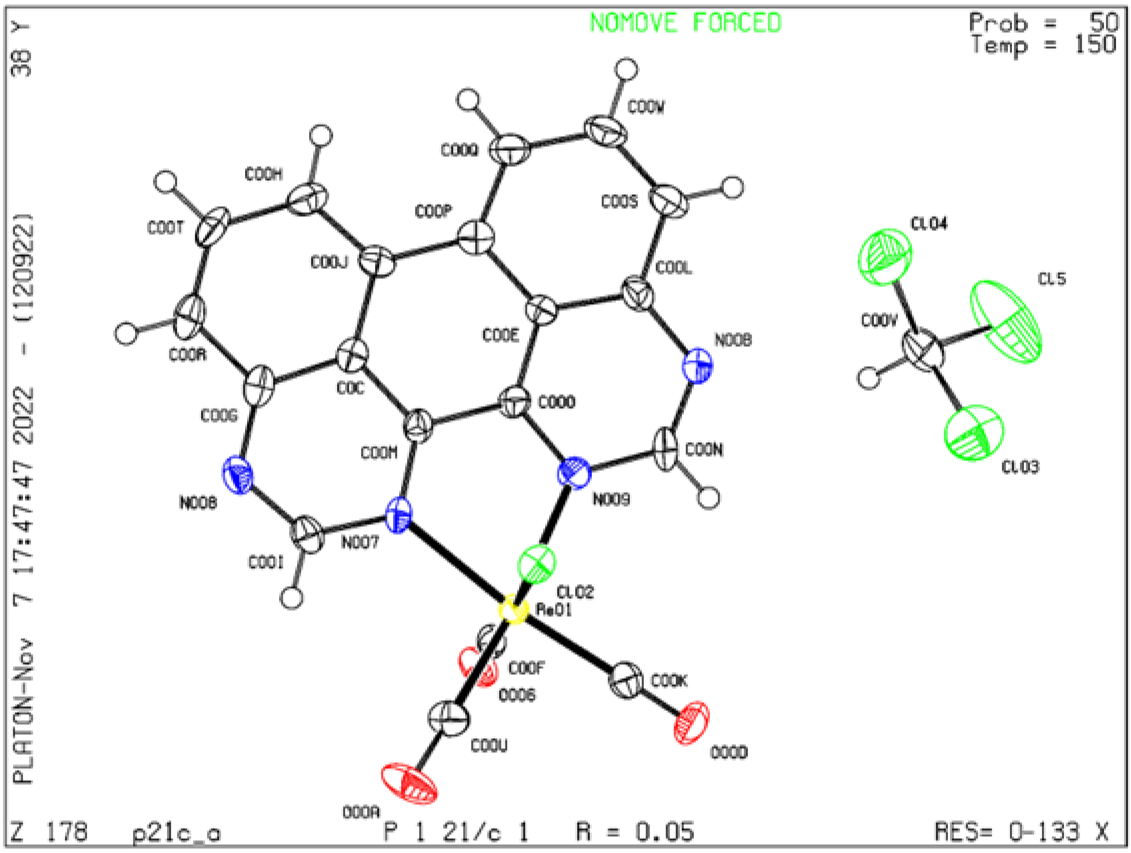

Figure S38: ORTEP depiction of **3**; the ellipsoids are drawn at the 50% probability level. H-atoms were added at fixed positions.

5. Electrochemical characterization

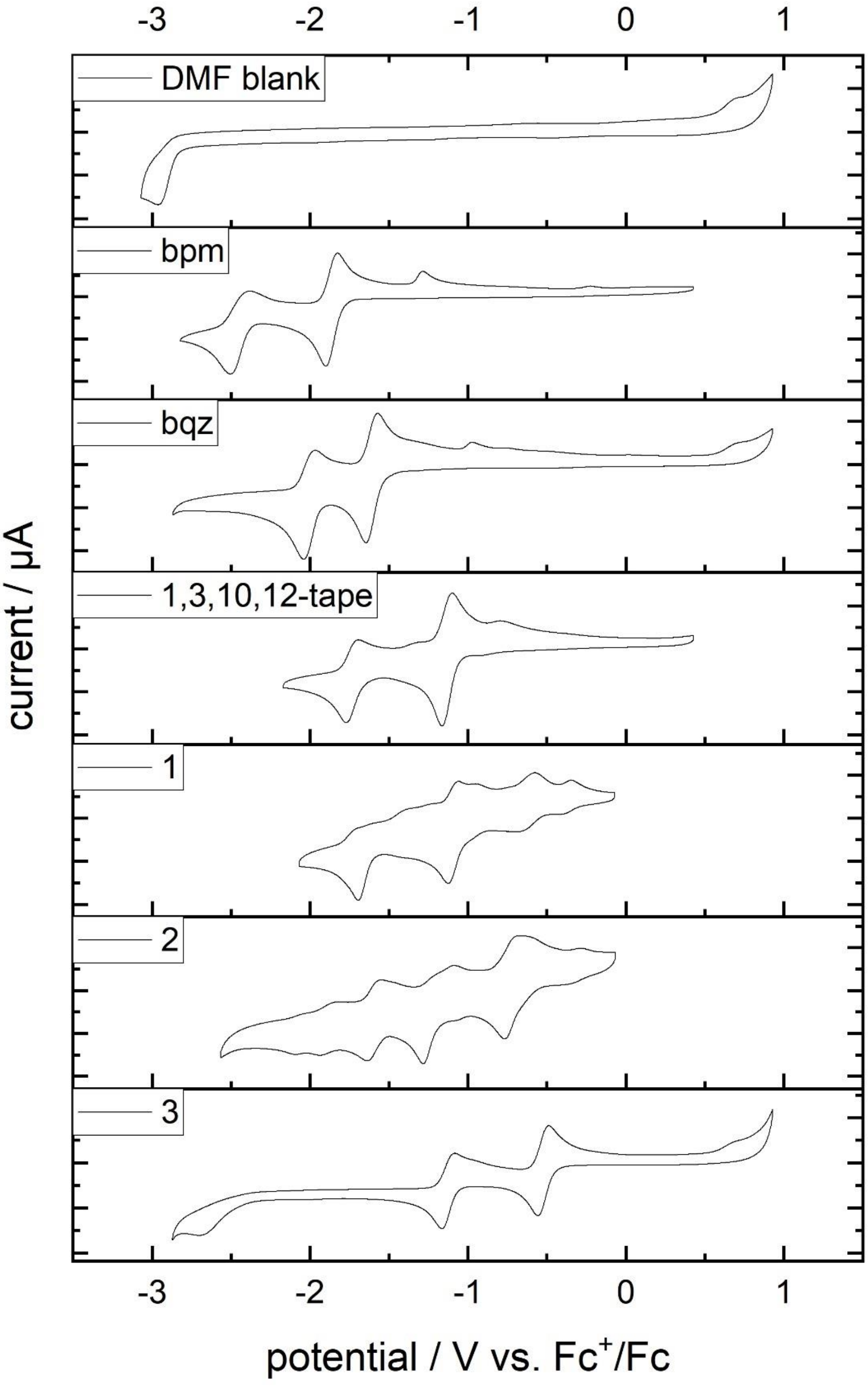

Figure S39: Cyclic voltammograms of ligands and complexes showing all observed reductions. (DMF, 0.1 M *n*Bu_4NPF_6 supporting electrolyte, 1 mM analyte, 100 mV s^{-1}, GC-WE, Pt-wire CE, non-aqueous $AgNO_3$/Ag-RE (0.1 M nBu_4NPF_6 / 0.01 mM $AgNO_3$ in MeCN), referenced against Fc^+/Fc).

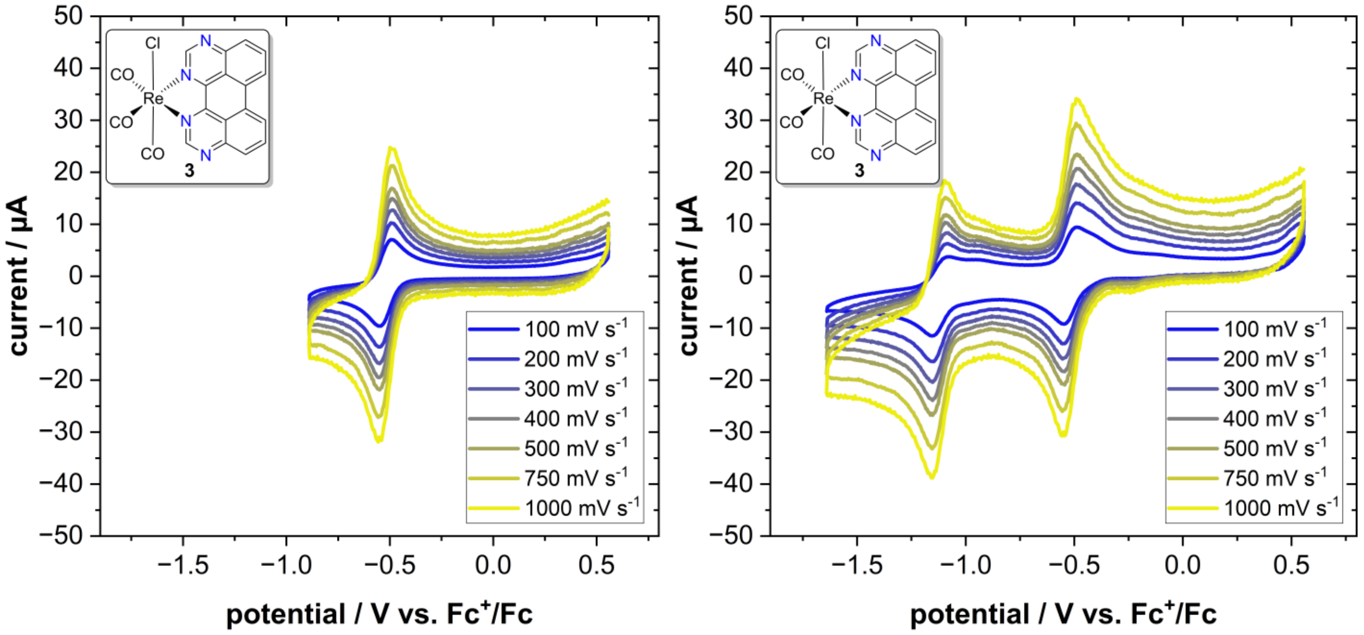

Figure S40: Cyclic voltammograms of complex **3** showing left) the first and right) the first and second reductions at variable scan rates. (DMF, 0.1 M nBu$_4$NPF$_6$ supporting electrolyte, 1 mM **3**, GC-WE, Pt-wire CE, non-aqueous Ag-wire pseudo-RE (0.1 M nBu$_4$NPF$_6$ in DMF), referenced against Fc$^+$/Fc).

6. UV/Vis-NIR absorption and emission spectra

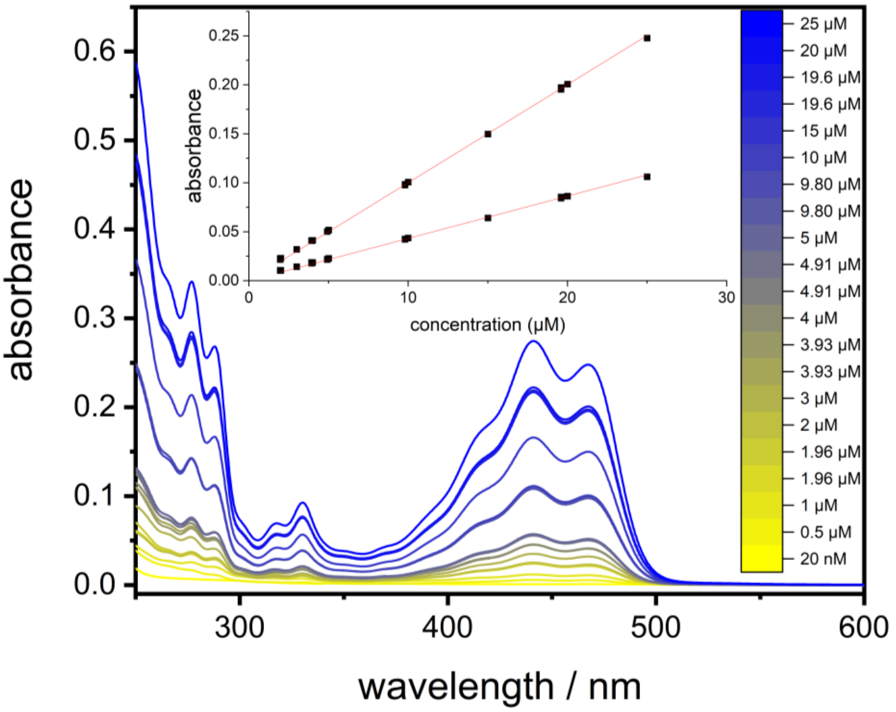

Figure S41: UV-vis absorption spectra of 1,3,10,12-tape in CHCl$_3$ at concentrations from 20 nM to 25 µM. Inset shows absorbance vs. concentration plot and least-squares best-fit lines at wavelengths of 440.5 and 468.0 nm, respectively.

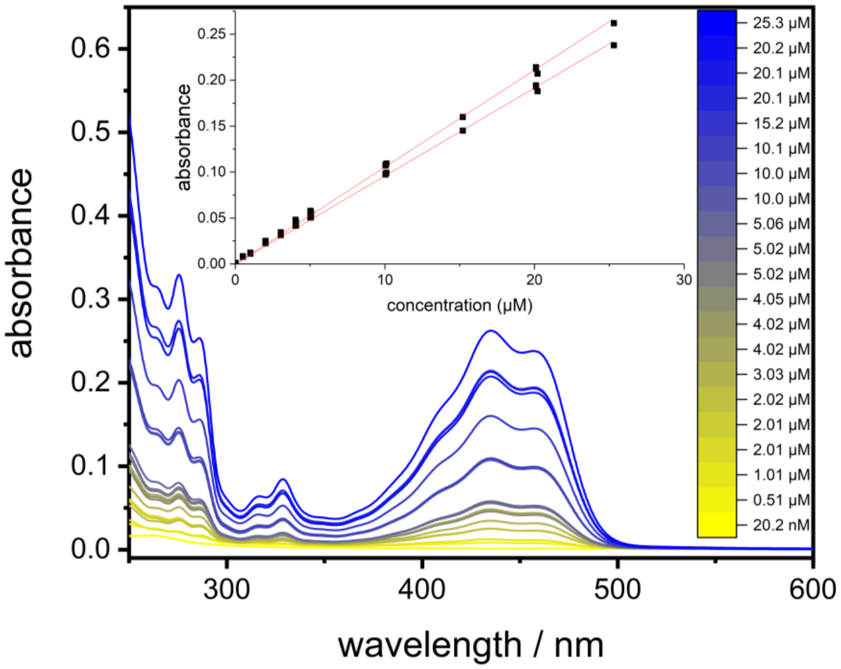

Figure S42: UV-vis absorption spectra of 1,3,10,12-tape in CH3CN at concentrations from 20 nM to 25 μM. Inset shows absorbance vs. concentration plot and least-squares best-fit lines at wavelengths of 434.0 and 457.5 nm, respectively.

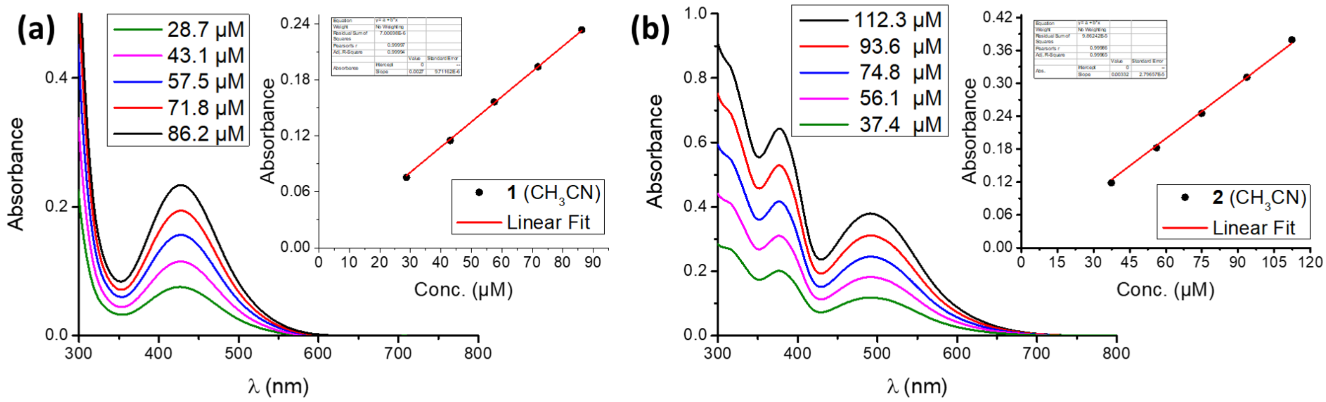

Figure S43: UV-vis absorption spectra of complex (a) **1** and (b) **2** in CH3CN at their various concentrations. Inset shows absorbance *vs* concentration plots and least-squares best-fit lines at wavelengths of 427 nm, and 491 nm, respectively.

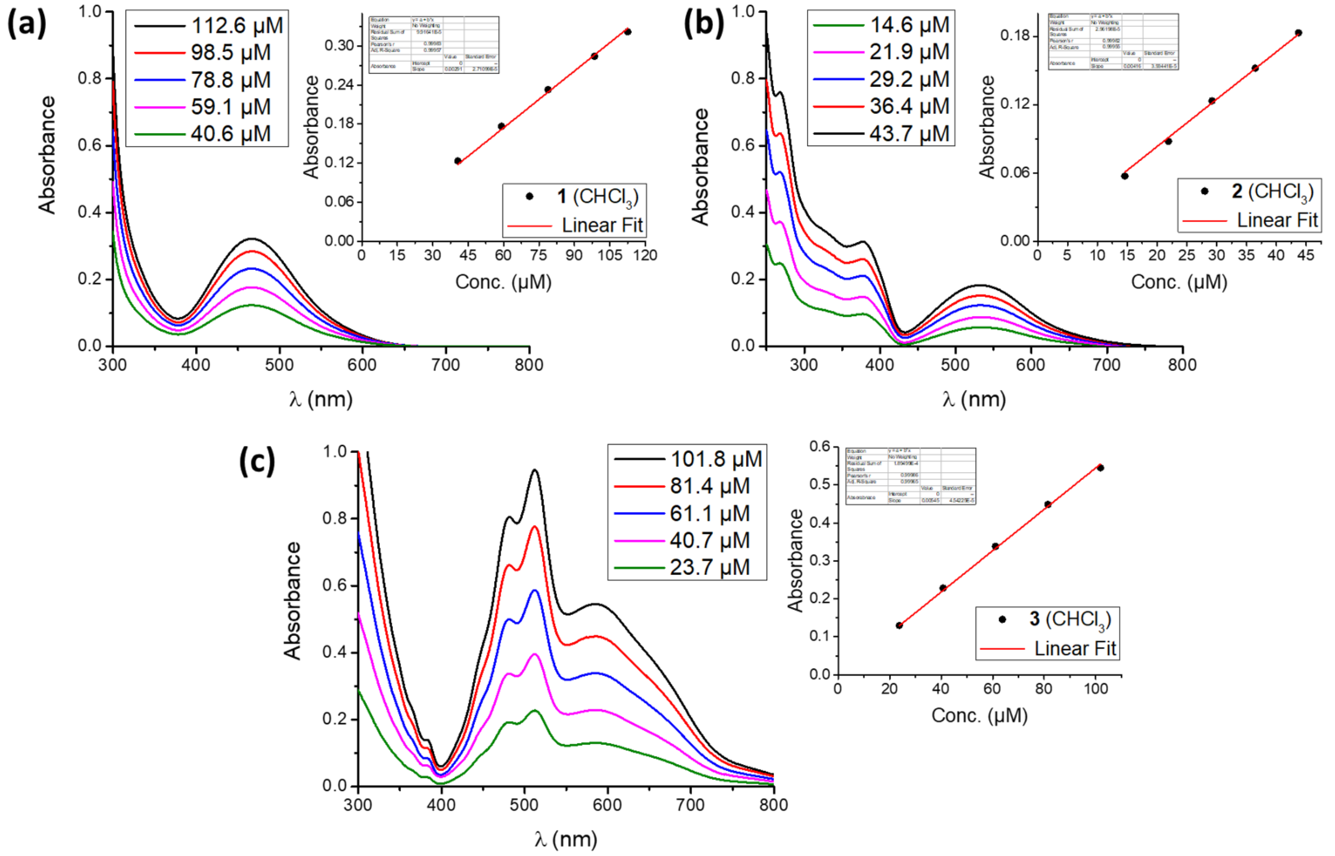

Figure S44: UV-vis absorption spectra of complex (a) **1**, (b) **2** and (c) **3** in $CHCl_3$ at their various concentrations. Inset shows absorbance *vs* concentration plots and least-squares best-fit lines at wavelengths of 467 nm, 532 nm, and 586 nm, respectively.

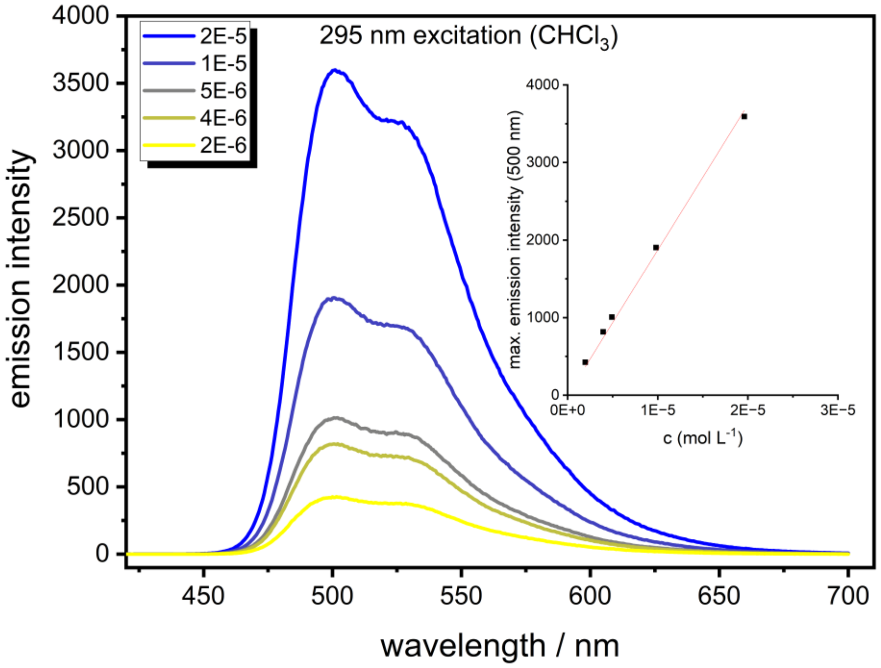

Figure S45: Luminescence spectra of **1,3,10,12-tape** in chloroform solution at different concentrations (see legend) upon excitation at 295 nm with the inset showing the maximum emission intensity *vs*. concentration and least-square best-fit line.

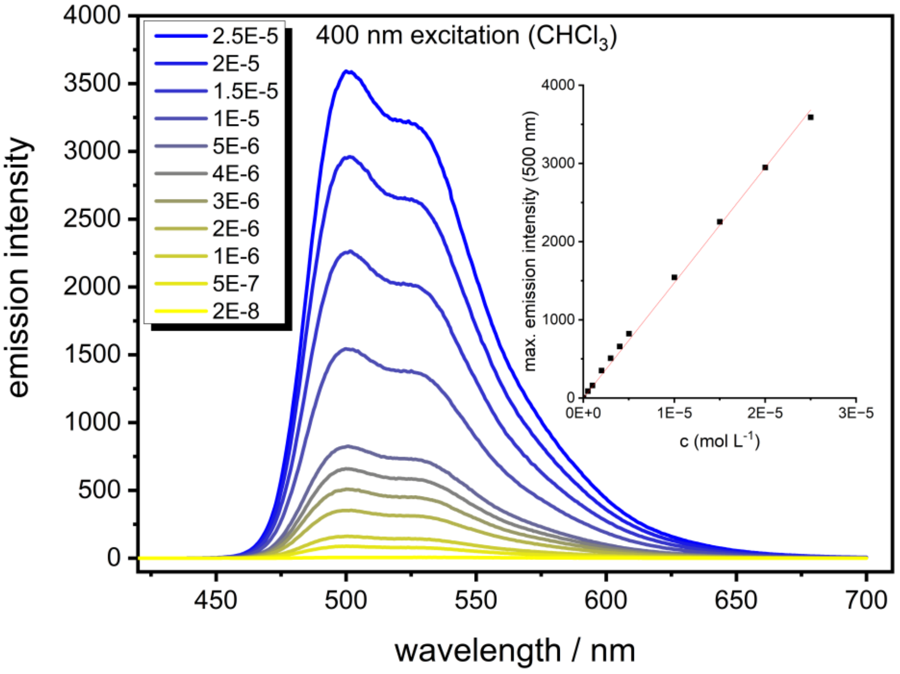

Figure S46: Luminescence spectra of **1,3,10,12-tape** in chloroform solution at different concentrations (see legend) upon excitation at 400 nm with the inset showing the maximum emission intensity *vs.* concentration and least-square best-fit line.

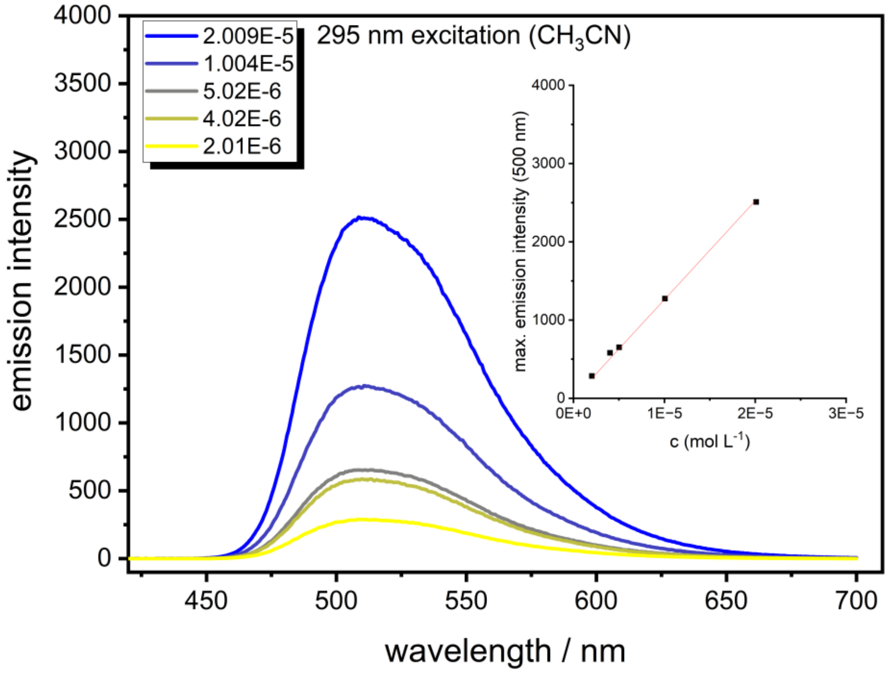

Figure S47: Luminescence spectra of **1,3,10,12-tape** in acetonitrile solution at different concentrations (see legend) upon excitation at 295 nm with the inset showing the maximum emission intensity *vs.* concentration and least-square best-fit line.

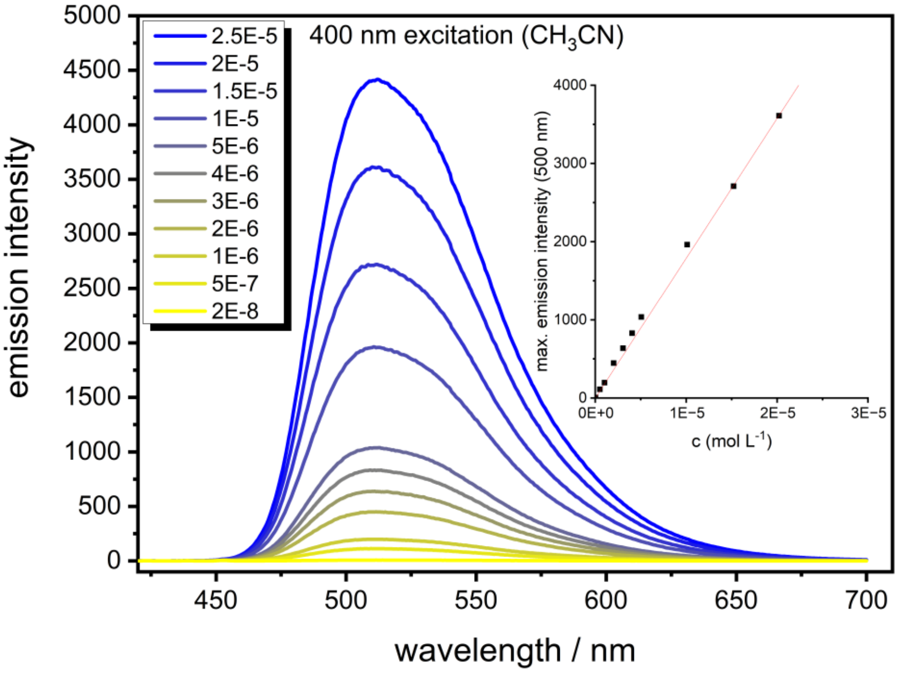

Figure S48: Luminescence spectra of **1,3,10,12-tape** in acetonitrile solution at different concentrations (see legend) upon excitation at 400 nm with the inset showing the maximum emission intensity *vs.* concentration and least-square best-fit line.

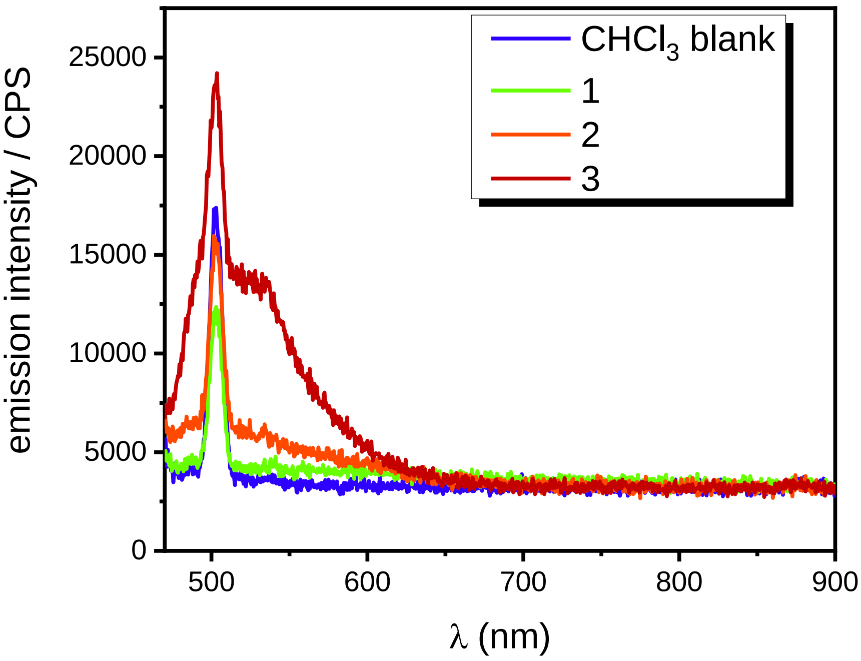

Figure S49: Uncorrected luminescence spectra of [Re(CO)$_3$(NN)Cl] complexes **1**-**3** in air-saturated chloroform with optical densities of 0.1 at the excitation wavelength of 436 nm. No complex emission was observed, however for complex **3** traces of the free azaperylene ligand are observed in emission spectroscopy. Neither a wider bandpass, removal of oxygen, change of solvent to THF, nor increasing the integration time led to the observation of complex emission at room temperature.

6. UV-Vis-NIR spectroelectrochemical measurements

In the following, the UV-Vis-NIR spectral progressions of ligand **1,3,10,12-tape** and complexes **1-3** are shown. Note that the cyclic voltammograms given as inset were measured using a glassy carbon WE, a Pt-wire CE and a non-aqueous $AgNO_3$/Ag pseudo-RE, whereas spectroelectrochemical measurements were performed using a Pine honeycomb screen-printed platinum electrode as WE and CE and an Ag-wire pseudo-RE as described in detail in the experimental part. The CVs shown were taken from the last scan (segments 8/9), where additional peaks had appeared.

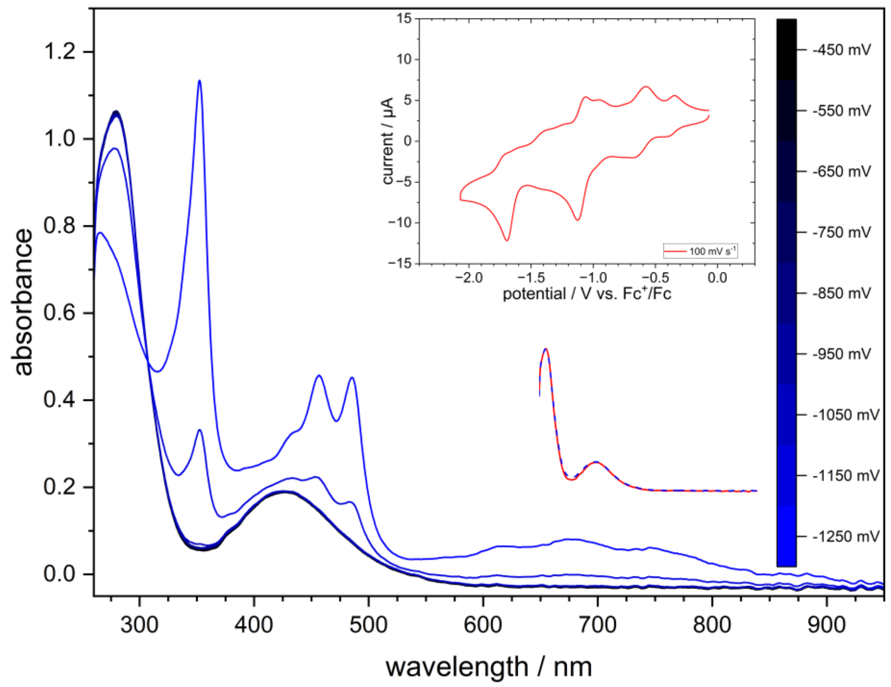

Figure S50: UV-Vis-NIR spectra obtained during spectroelectrochemical <u>first</u> reduction of complex **1** (0.5 mM) in dry, deaerated DMF containing 0.1 M $n$$Bu_4PF_6$, 90-180 s equilibrated potentials are given in V *vs.* Fc^+/Fc, the insets showing the cyclic voltammogram and the spectra obtained before electrolysis and after reversed electrolysis (-450 mV).

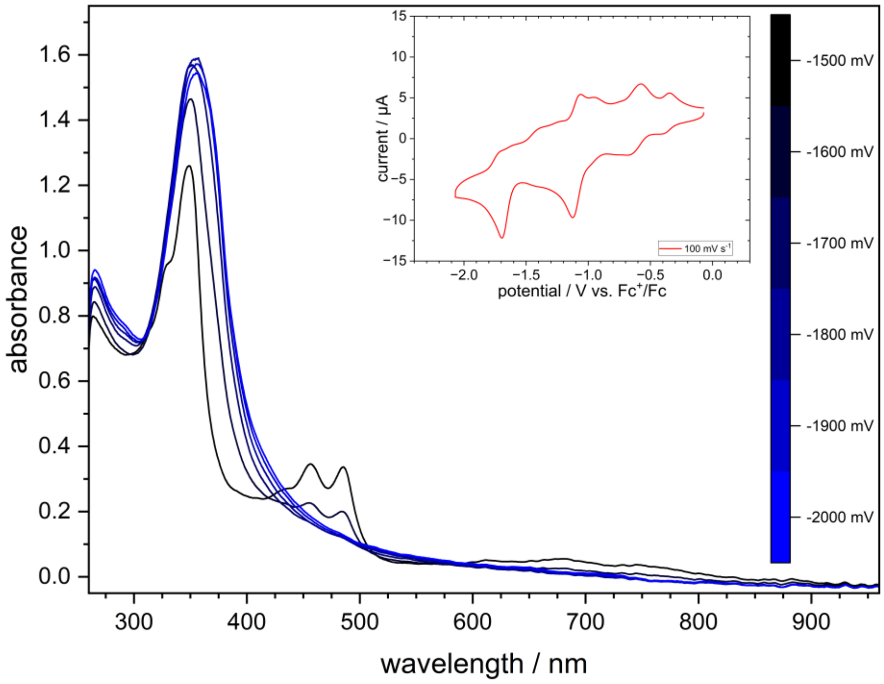

Figure S51: UV-Vis-NIR spectra obtained during spectroelectrochemical second reduction of complex **1** (0.5 mM) in dry, deaerated DMF containing 0.1 M nBu$_4$PF$_6$, 90-180 s equilibrated potentials are given in V *vs.* Fc$^+$/Fc, the inset showing the cyclic voltammogram. The original spectrum could not be regained after reversed electrolysis.

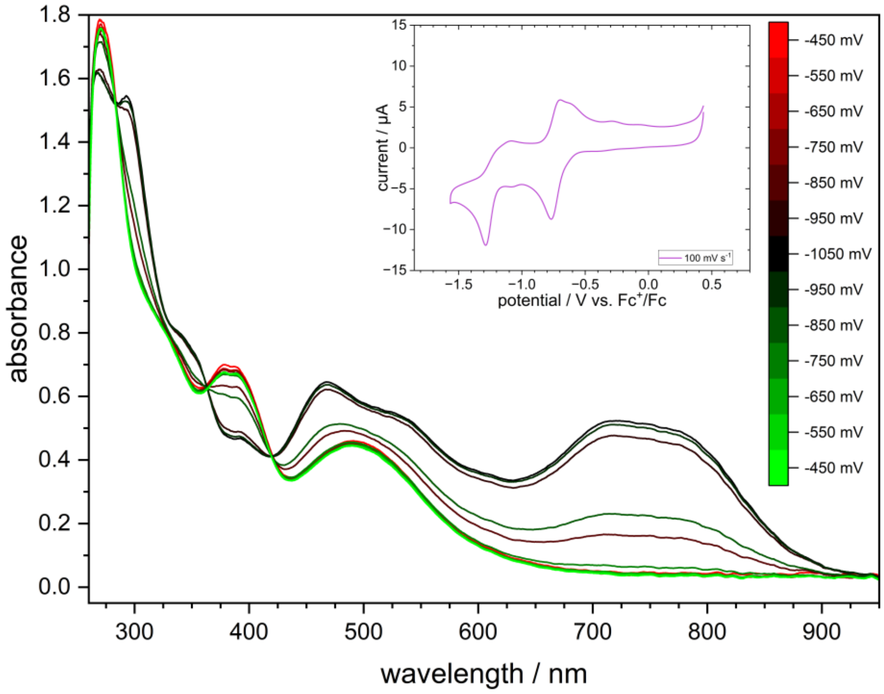

Figure S52: UV-Vis-NIR spectra obtained during spectroelectrochemical first reduction of complex **2** (0.5 mM) in dry, deaerated DMF containing 0.1 M nBu$_4$PF$_6$, 90-180 s equilibrated potentials are given in V *vs.* Fc$^+$/Fc, the inset showing the cyclic voltammogram. The original spectrum was regained after reversed electrolysis (-450 mV).

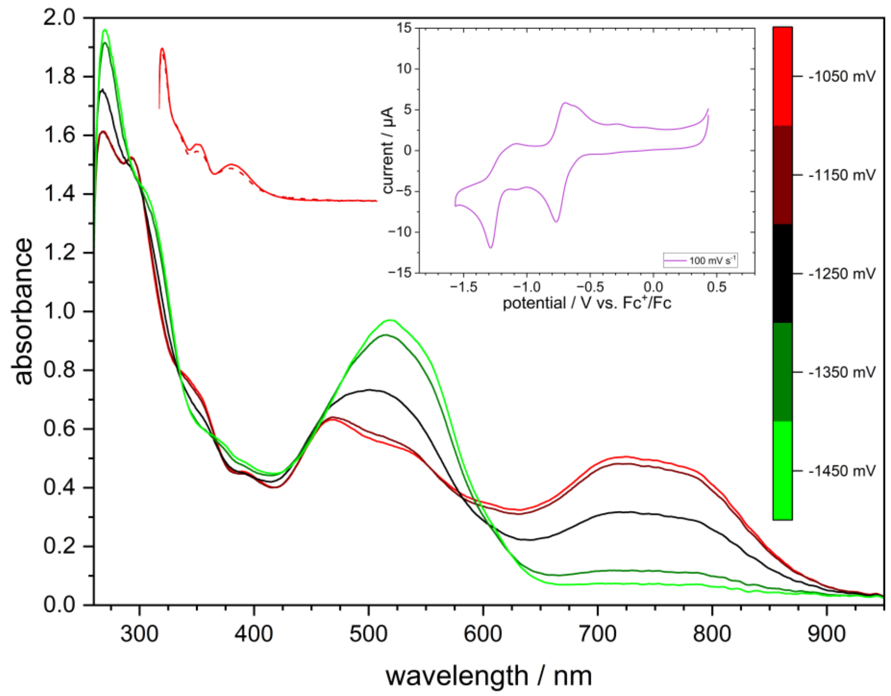

Figure S53: UV-Vis-NIR spectra obtained during spectroelectrochemical <u>second</u> reduction of complex **2** (0.5 mM) in dry, deaerated DMF containing 0.1 M *n*Bu_4PF_6, 90-180 s equilibrated potentials are given in V *vs.* Fc^+/Fc, the insets showing the cyclic voltammogram and the spectra obtained before electrolysis and after reversed electrolysis (-450 mV).

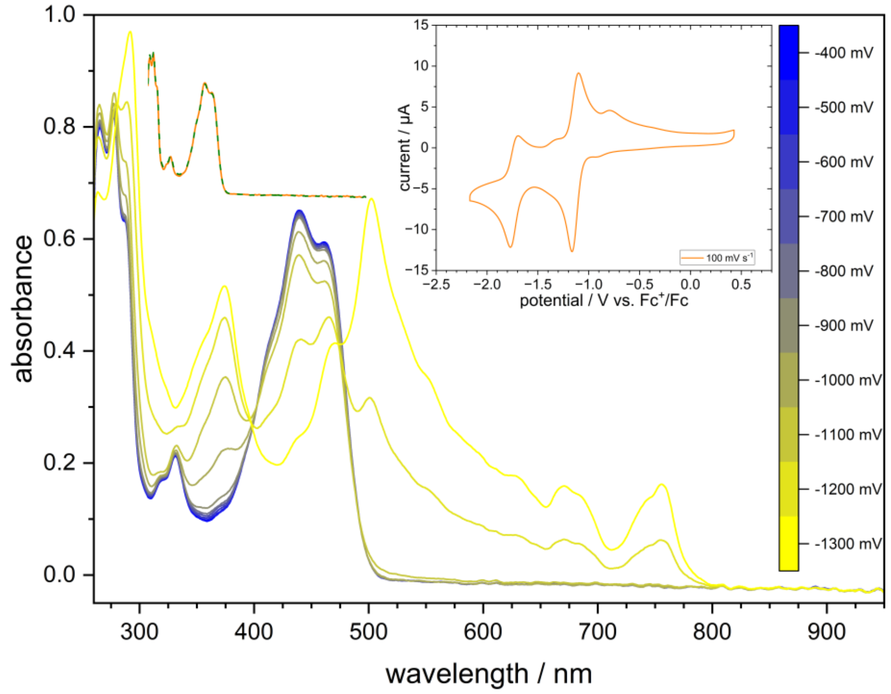

Figure S54: UV-Vis-NIR spectra obtained during spectroelectrochemical <u>first</u> reduction of ligand **1,3,10,12-tape** (0.5 mM) in dry, deaerated DMF containing 0.1 M *n*Bu_4PF_6, 90-180 s equilibrated potentials are given in V *vs.* Fc^+/Fc, the insets showing the cyclic voltammogram and the spectra obtained before electrolysis and after reversed electrolysis (-400 mV).

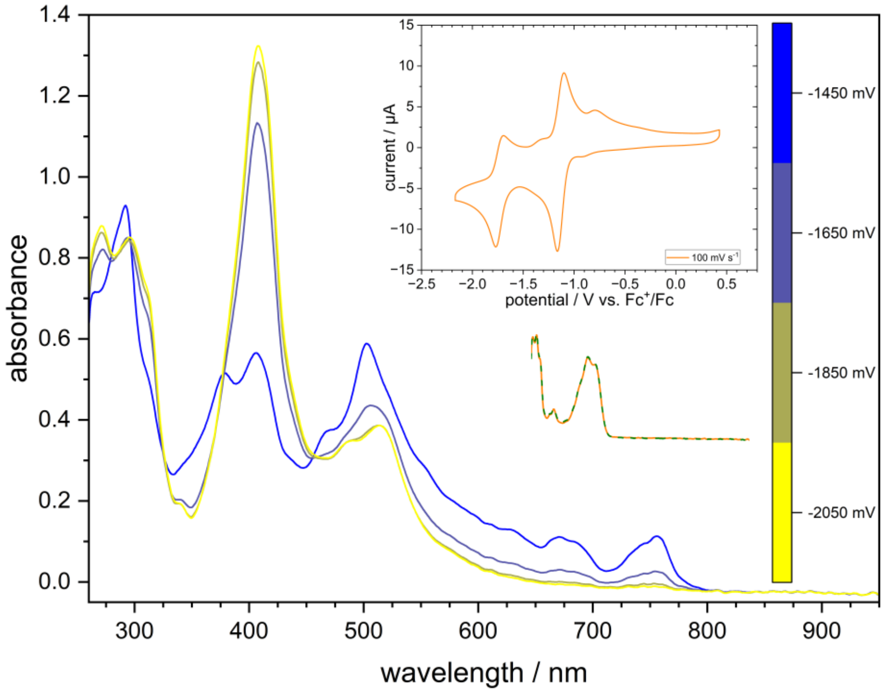

Figure S55: UV-Vis-NIR spectra obtained during spectroelectrochemical <u>second</u> reduction of ligand **1,3,10,12-tape** (0.5 mM) in dry, deaerated DMF containing 0.1 M *n*Bu_4PF_6, 90-180 s equilibrated potentials are given in V *vs.* Fc^+/Fc, the insets showing the cyclic voltammogram and the spectra obtained before electrolysis and after reversed electrolysis (-450 mV).

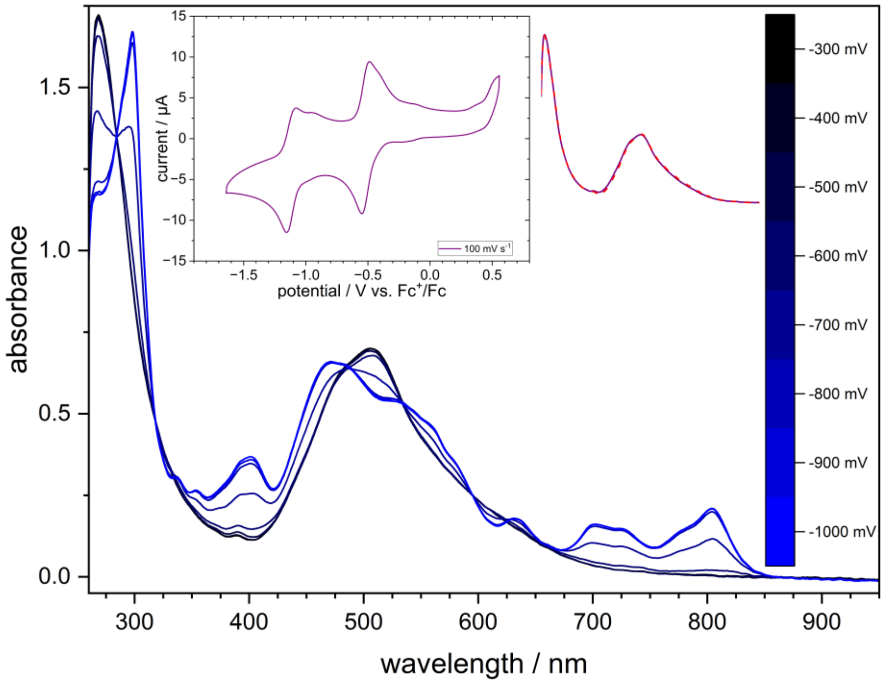

Figure S56: UV-Vis-NIR spectra obtained during spectroelectrochemical <u>first</u> reduction of complex **3** (0.5 mM) in dry, deaerated DMF containing 0.1 M *n*Bu_4PF_6, 90-180 s equilibrated potentials are given in V *vs.* Fc^+/Fc, the insets showing the cyclic voltammogram and the spectra obtained before electrolysis and after reversed electrolysis (-300 mV).

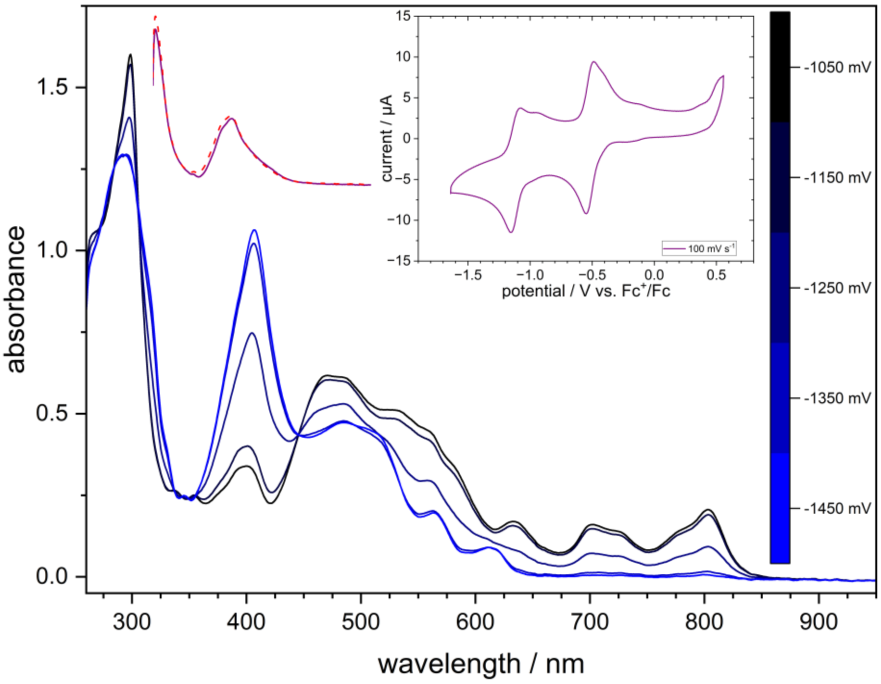

Figure S57: UV-Vis-NIR spectra obtained during spectroelectrochemical <u>second</u> reduction of complex **3** (0.5 mM) in dry, deaerated DMF containing 0.1 M *n*Bu_4PF_6, 90-180 s equilibrated potentials are given in V *vs.* Fc^+/Fc, the insets showing the cyclic voltammogram and the spectra obtained before electrolysis and after reversed electrolysis (-250 mV).

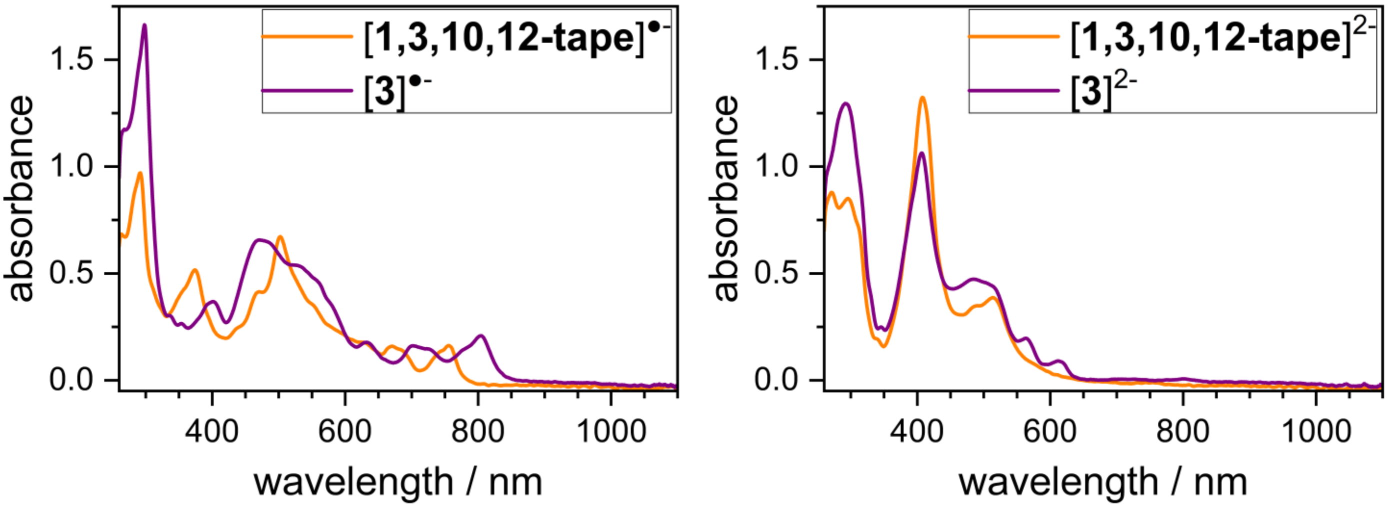

Figure S58: Direct comparison of the UV-Vis-NIR spectra obtained during spectroelectrochemical reduction of ligand **1,3,10,12-tape** and complex **3** (0.5 mM) in dry, deaerated DMF containing 0.1 M *n*Bu_4PF_6, in their mono- and dianionic states.

7. Nanosecond transient absorption spectra

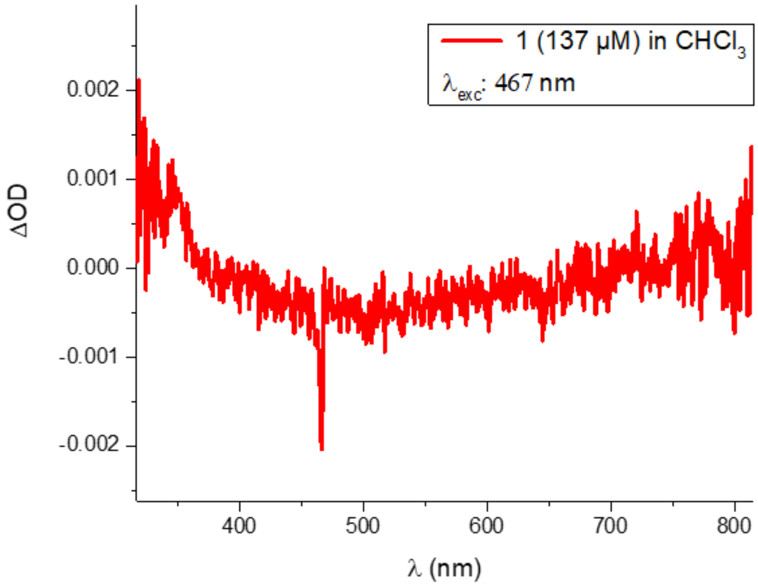

Figure S59: Nanosecond-transient absorption spectrum of [Re(CO)3(bpm)Cl] (**1**) dissolved in deaerated chloroform (137 μM) upon 467 nm pump excitation.

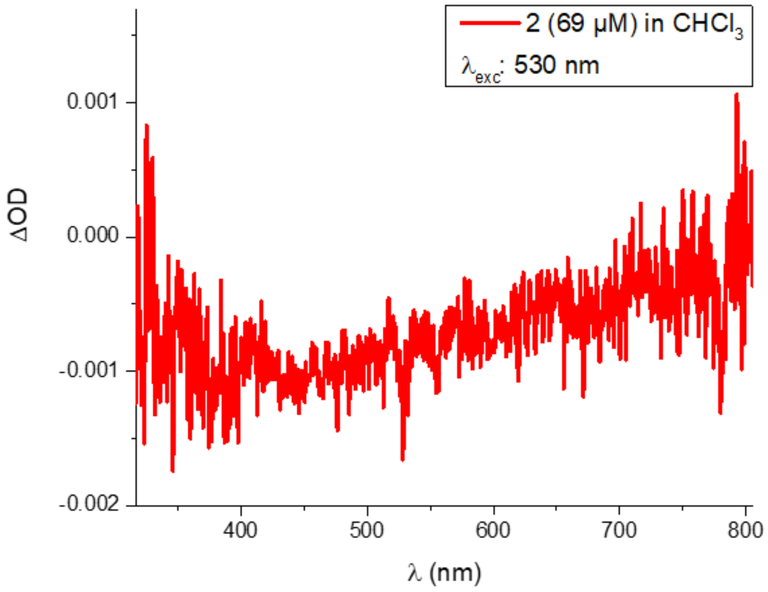

Figure S60: Nanosecond-transient absorption spectrum of [Re(CO)3(bqz)Cl] (**2**) dissolved in deaerated chloroform (69 μM) upon 530 nm pump excitation.

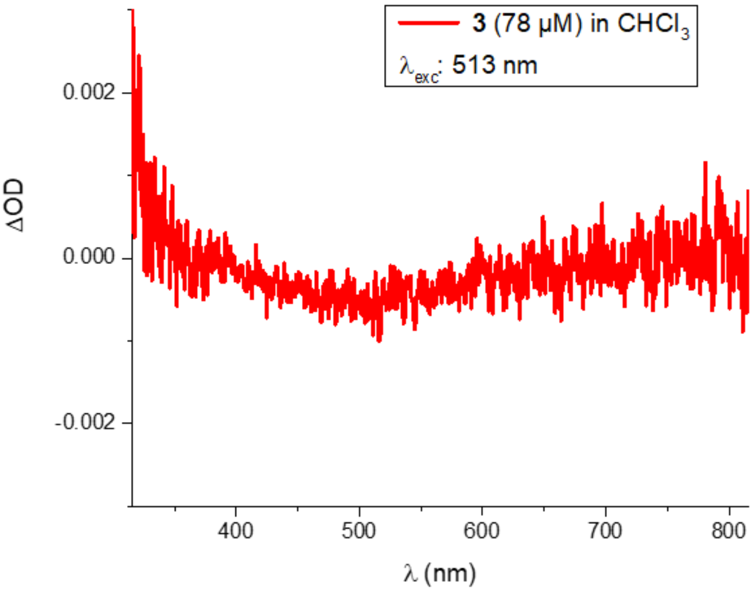

Figure S61: Nanosecond-transient absorption spectrum of [Re(CO)$_3$(1,3,10,12-tape)Cl] (**3**) dissolved in deaerated chloroform (78 μM) upon 513 nm pump excitation.
